# Comprehensively Surveying Structure and Function of RING Domains from *Drosophila melanogaster*

**DOI:** 10.1371/journal.pone.0023863

**Published:** 2011-09-02

**Authors:** Muying Ying, Xiaotian Huang, Haijun Zhao, Yuehao Wu, Fusheng Wan, Chunhong Huang, Kemin Jie

**Affiliations:** 1 Department of Molecular Biology and Biochemistry, Basic Medical College of Nanchang University, Nanchang, People's Republic of China; 2 Department of Medical Microbiology, Basic Medical College of Nanchang University, Nanchang, People's Republic of China; 3 Department of Gynecology and Obstetrics, ZhongDa Hospital, Southeast University, Nanjing, People's Republic of China; 4 Academy of Agricultural Sciences of Jiangxi, Nanchang, People's Republic of China; Institute of Molecular and Cell Biology, Singapore

## Abstract

Using a complete set of RING domains from *Drosophila melanogaster*, all the solved RING domains and cocrystal structures of RING-containing ubiquitin-ligases (RING-E3) and ubiquitin-conjugating enzyme (E2) pairs, we analyzed RING domains structures from their primary to quarternary structures. The results showed that: i) putative orthologs of RING domains between *Drosophila melanogaster* and the human largely occur (118/139, 84.9%); ii) of the 118 orthologous pairs from *Drosophila melanogaster* and the human, 117 pairs (117/118, 99.2%) were found to retain entirely uniform domain architectures, only Iap2/Diap2 experienced evolutionary expansion of domain architecture; iii) 4 evolutionary structurally conserved regions (SCRs) are responsible for homologous folding of RING domains at the superfamily level; iv) besides the conserved *Cys/His* chelating zinc ions, 6 equivalent residues (4 hydrophobic and 2 polar residues) in the SCRs possess good-consensus and conservation- these 4 SCRs function in the structural positioning of 6 equivalent residues as determinants for RING-E3 catalysis; v) members of these RING proteins located nucleus, multiple subcellular compartments, membrane protein and mitochondrion are respectively 42 (42/139, 30.2%), 71 (71/139, 51.1%), 22 (22/139, 15.8%) and 4 (4/139, 2.9%); vi) CG15104 (Topors) and CG1134 (Mul1) in C3HC4, and CG3929 (Deltex) in C3H2C3 seem to display broader E2s binding profiles than other RING-E3s; vii) analyzing intermolecular interfaces of E2/RING-E3 complexes indicate that residues directly interacting with E2s are all from the SCRs in RING domains. Of the 6 residues, 2 hydrophobic ones contribute to constructing the conserved hydrophobic core, while the 2 hydrophobic and 2 polar residues directly participate in E2/RING-E3 interactions. Based on sequence and structural data, SCRs, conserved equivalent residues and features of intermolecular interfaces were extracted, highlighting the presence of a nucleus for RING domain fold and formation of catalytic core in which related residues and regions exhibit preferential evolutionary conservation.

## Introduction

Almost all eukaryotic organisms possess numerous RING proteins. E3 ubiquitin-ligase (E3) activity is intrinsic to RING domains of c-Cbl, AO7, and seven other randomly selected RING proteins, and is likely to be a general function of the domain. Numerous RING proteins are likely to belong to RING-containing domain ubiquitin-ligases (RING-E3s) [1]. RING-E3s, collectively representing the large majority of E3s, have been linked to control many cellular processes such as DNA repair, cell cycle and division, and host defense. Their dysregulation has been implicated in many pathophysiological disease states such as hypoxia, cancer, and liver fibrogenesis [2]. These observations along with the fact that RING domains determine specificity of ubiquitination by recognizing substrate and mediating transfer of ubiquitin from ubiquitin-conjugating enzyme (E2) to substrate, inspired investigators to design pharamacologic agents specific for them. Although our knowledge of E3s as therapeutic targets is still limited [3], [4], [5], [6], [7], [8], [9], [10], [11] (Table 1), several RING-E3s, such as the APC11, the SCF complex, and the MDM2 protein have been well-established as ideal targets for drug discovery and development [12], [13].

**Table 1:** The target proteins and the related disease for the known RING-E3s and the potential drug for therapy.

Gene name	Target	Disease	Potential Drug	Reference
Hdm2/Mdm2	P53	Apoptosis, tumor	HLI98, Nutlin, RITA and MI-17	[3]
Apc11	Cyclin B, Securin	Tumor	Hydrogen peroxide	[4]
Cbl	PI3K/Akt signaling	Apoptosis	Arsenic	[5]
Smurf1	Smad1 and Smad5	Pancreatic cancer; Osteosarcoma	Bortezomib	[6]
Rnf4	Pml	APL	Arsenic; ATRA	[7]
BirC2,3, 4	Traf1/Traf2	Tumor	Bortezomib	[8]
Parkin	Pink1	Parkinson	Levodopa	[9]
Murf1, Mafbx	eIF3-f, MyoD, troponin I	Skeletal muscle atrophy	Des-acyl ghrelin	[10]
Traf1/Traf2	IAPs	Apoptosis	EBV	[11]

Previously, genome-wide functional analysis of RING proteins have been performed in the human and *Arabidopsis thaliana* [14], [15]. Mutational experiments on RING domains have been conducted by combining bioinformatic analysis of structure and computation, which provided us the first example of the altered specificity of RING-E3 and E2 pairs and insight into how this specificity is obtained [16], [17]. Despite the availability of structural and functional data about RING-E3s and E2/RING-E3 pairs, little progress has been made in understanding the molecular basis and principles responsible for RING domain functional similarity and structural diversity, and the specificity of E2/RING-E3 interactions. The data became increasingly intractable due to: i) a single RING-E3 functioning with a set of E2s, and vice versa; and ii) extensive cross-talk of the ubiquitin system with others [18].

Rapid advances in evolutionary genomics and structural bioinformatics, together with the availability of an ever-growing number of genome sequences and previously solved three-dimensional (3D) structures of RING domains, it is now possible for us to extract sequence, structural, and functional information from the evolutionary history of RING protein superfamily by comparative and structural genomic approaches. To better understand the molecular basis and principally responsible factors for the similarity and diversity of RING-E3 functions and E2/RING-E3 interactions, we comprehensively analyzed a complete set of RING domains of *Drosophila melanogaster* and all the solved RING domains from primary to tertiary structure, compared domain architecture and subcellular localization of RING proteins of *Drosophila melanogaster*, mapped interolog interactions of RING-E3 and E2 pairs, and pinpointed the intermolecular interface features of 3D complexes of E2/RING-E3 pairs, and the results showed that:

1. By comparative genomic approaches, a complete set of 139 nonredundant RING proteins from *Drosophila melanogaster* were identified and classified based on the shared sequence patterns of the conserved *Cys/His* residues.
2. Based on the notions: a) one-on-one mapping of protein functionality across species is a critical component of comparative genomic analysis [19]; and b) orthologs provide useful information in identification of protein function, we defined 118 putative orthologs of RING domains between the human and *Drosophila melanogaster* by Reciprocal Best Blast Hits [20], which accounts for 84.9% (118/139, 84.9%).
3. Analyzing sequence and structural elements based on multiple alignments indicated that the large majority of RING domains have a similar second structural arrangement of ββα motif, which appears to be highly efficient for structural stabilization of RING domains.
4. Of the 118 orthologous pairs from *Drosophila melanogaster* and the human, 117 pairs (117/118, 99.2%) were found to retain entirely uniform domain architectures, only Diap2/CG8293 of *Drosophila melanogaster* IAP family experienced evolutionary expansion of domain architecture. Additional domain analysis showed that several zinc-binding domains (ZnF, BBOX, Sina/Siah, IBR and PHD) widespread occurred in these RING proteins.
5. Data from all the solved RING domains showed that 4 regions (N-loop, the first β-sheet region, βα-region, and C-loop) are responsible for the homologous folding of RING domains at the superfamily level across long evolutionary periods, and belong to evolutionary structurally conserved regions (SCRs) (average RMSD values of RING/non-U-box = 0.9, 1.64, 1.56, and 0.8; RMSD: root-mean-square deviation).
6. Using sequence consensus levels and conservation indices, we defined consensus and conservation of 4 hydrophobic residues and 2 polar residues located at the SCRs of RING domains. The 4 SCRs function in the appropriate positioning of the 6 equivalent residues as structural determinants for RING-E3 catalysis.
7. Surveying spatial distribution of residues in RING domain 3D structures showed that the 4 hydrophobic residues promote the formation of the conserved core of solvent inaccessibility and consolidate within a large hydrophobic patch flanked by the 2 polar residues.
8. Subcellular localization analysis showed that members of RING proteins located nucleus, multiple subcellular compartments, membrane protein and mitochondrion are respectively 42 (42/139, 30.2%), 71 (71/139, 51.1%), 22 (22/139, 15.8%) and 4 (4/139, 2.9%). Of C3HC4 type, 34 members (34/68 = 50%) are located nucleus, while 21 of C3H2C3 (21/29 = 72.4%) are located multiple subcellular compartments.
9. Mapping interolog interactions of E2/RING-E3 pairs showed that CG15104 (Topors) and CG1134 (Mul1) in C3HC4, and CG3929 (Deltex) in C3H2C3 seem to display broader E2s binding profiles than other RING-E3s in *Drosophila melanogaster* ubiquitination system.
10. Analyzing all the solved and modeled 3D complexes indicated that their intermolecular interfaces form conserved hydrophobic contacts (CHCs) with E2s. Residues of RING domains directly interacting with E2s are all from the SCRs of RING domains. Of the 6 residues in RING domains, 2 hydrophobic ones contribute to constructing the conserved hydrophobic core of the solvent inaccessibility, and 2 hydrophobic residues and 2 polar residues directly participate in E2/RING-E3 interactions by hydrophobic and electrostatic interactions.

By analyzing RING domains across primary through tertiary structures, pinpointing the intermolecular interface features of 3D complexes, comparing domain architecture and detecting subcellular localization of RING proteins from *Drosophila melanogaster*, the study offered a new perspective for better understanding molecular link between structural conservation and diversification, and a functional similarity and specificity of RING domains, and E2/RING-E3 interactions involved in RING-E3 catalysis.

## Results and Discussion

### Identification and Classification of RING Proteins from *Drosophila melanogaster*

A complete set of 139 RING proteins from *Drosophila melanogaster* were identified with extensive database searches and followed by manual curation to remove truncated and/or redundant sequences. Based on the shared sequence conserved patterns of the corresponding site residue-binding zinc ions, RING proteins identified here were subdivided into eight types: C3HC4, C3H2C3 (RING-H2), C3HC3D, C4HC3 (RINGv), C3HGC3 (RING-G), C4C4 (RING-C2), C6H3C2D, and U-box (Table S1 and Figure S1). Two types of RING-D and RING-S/T (with *Ser* or *Thr* substitutions at one or both metal ligand positions 2 and 6) detected in *Arabidopsis thaliana* could not be identified in *Drosophila melanogaster* [15]. Both the RING-D and C3HC3D types have an *Asp* substitution at a metal ligand position, but their metal ligand positions are different (the former at position 5 and the latter at position 8). Members of the C3HGC3 type have a *Gly* substitution at metal ligand position 5. One or 2 members (such as CG3639, CG2681, and CG3231) with substitutions at a different metal ligand position (Figure S1, shaded yellow) were not subdivided into an independent type due to an inadequate number of members.

Of the 139 RING proteins, 118 (118/139, 84.9%) were found to be putative orthologs from the human (Tables 2, S1). With high percentages of alternatively spliced transcript (45/118, 38.1%), the 118 orthologs from *Drosophila melanogaster* encode 220 mRNA of RING proteins. Alternative splicing can generate more transcripts from a single gene than the number of genes in an entire genome. Previous studies showed that duplicated genes have fewer alternative splicing isoforms than single-copy genes, and that recent duplicates usually lose alternative splicing isoforms, while the ancient duplicates could evolve new alternative splicing isoforms during evolutionary process [21]. We proposed that the ancient RING proteins experienced evolutionary expansions at transcriptional level by alternative splicing. The 21 RING proteins of *Drosophila melanogaster* (139–118, 21) without the putative orthologs from the human independently occurred in C3HC4 (9), C3H2C3 (4), C4HC3 (3) and C3HGC3 (5) of *Drosophila melanogaster*. With less alternatively spliced transcript, the 21 RING proteins encode 25 mRNA of RING proteins, of which 16 lack themselves alternative splicing isoforms. As basic unit of genes, and footprints of origin, exons in genes are highly suitable for studying origin and evolution of genes [22]. To trace the origin of the 21 RING Proteins of *Drosophila melanogaster*, we performed BLAST search using each exon of the 21 RING proteins against *Drosophila melanogaster* genome reference sequence, and also compared the annotation of Transportable Elements available in RepeatMasker. Two long interspersed element (LINE), and one DNA element were identified to be involved in the exonization of the 21 RING proteins of *Drosophila melanogaster* (Figure S2 and Table 3). Without homologous sequences in the databases, 63 (63/67, 94.0%) obtained only one hit. Parsimoniously, those unmatched exons were mostly derived from unique intronic sequences. Only one (CG31053) had several matches, which may be originated from exon duplication (Figure S2). Therefore, exonization of intronic sequences, together with exonization of Transportable elements and exon duplication contributed to taxonomical independent evolutionary processes of *Drosophila melanogaster* RING proteins.

**Table 2:** Summary of different type RING domains from *Drosophila melanogaster*.

Type	Number (subcellular)	Orthologs	Percentage(%)	Accuracy	ID (%)
C3HC4	68 (34, 27, 4, 3)	59	48.9 (68/139)	75	27.2
C3H2C3	29 (1, 21, 7, 0)	25	20.9 (29/139)	79	28.9
C3HC3D	5 (1, 4, 0, 0)	5	3.6 (5/139)	84	36.0
C4HC3	17 (5, 4, 8, 0)	14	12.2 (17/139)	78	29.4
C3HGC3	6 (0, 5, 0, 1)	1	4.3 (6/139)	99	53.7
C4C4	3 (0, 1, 2, 0)	3	2.2 (3/139)	83	38.8
C6H3C2D	4 (0, 4, 0, 0)	4	2.9 (4/139)	96	53.5
U-box	7 (1, 5, 1, 0)	7	5.0 (7/139)	89	26.0
Total	139 (42, 71, 22, 4)	118	84.9 (118/139)	—	—
All the solved RING domains available currently					27.7
The solved C6H3C2D-type (C6H2C4-type) and RING/U-box					22.4

Note: Number, the number of different type RING domains of fruit fly, and subcellular means the number of RING proteins respectively located at nucleus, multiple subcellular compartments, membrane proteins and mitochondrion; Orthologs, the number of putative orthologs of different type RING domains between fruit fly and human; Percentage, the percentage of different type RING domains; Accuracy: the accuracy score of sequence alignments of different type RING domains evaluated using structural information; ID, the average percentage of sequence identity within different type RING domains of fruit fly and the solved RING domains.

**Table 3:** Hit number of exons of 21 RING proteins of fruit fly without putative orthologs from the human in BLAST search and RepeatMasker check.

Hits to LINE	Hits to DNA elements	1 hits	≥2 hits	Total exon
2	1	63	1	67

The occurrence of large numbers of orthologs between the human and *Drosophila melanogaster* along with a large number of orthologs between the human and *Saccharomyces cerevisiae* offer direct evidence in favor of the notion that RING proteins have experienced strong selective pressure for conservation throughout eukaryotic evolution [14]. This conclusion is also strengthened by the fact that there are similar frequencies of RING/U-box proteins between the human (8) and *Drosophila melanogaster* (7). Because the record of NP_689741 was removed as a result of standard genome annotation processing in the current version, there are 8 RING/U-box domain proteins in the human, while there were 9 RING/U-box domain proteins in the previous genome annotation. Human NP_001121684 is provided with a typical RING/U-box domain (E-value: 1.02e-23); however, its putative ortholog (NP_649969) from *Drosophila melanogaster* lacks the corresponding domain, resulting in RING/U-box proteins from *Drosophila melanogaster* having one less than the human (Table S1). With a similar percentage of assignments, RING/non-U-box and RING/U-box domain proteins are respectively encoded by 309 and 8 (97.5% and 2.5%) of 317 genes in the human, 132 and 7 (95% and 5.0%) of 139 in *Drosophila melanogaster*, and 47 and 2 (95.9% and 4.1%) of 49 in *Saccharomyces cerevisiae* [14]. Evidently, RING/non-U-box domains experienced evolutionary expansion with the increase of species complexity, and represent the large majority of the total RING proteins from various organisms [23]. With 68 members, RING/C3HC4 types are the largest followed by RING/C3H2C3 types in *Drosophila melanogaster* and the percent distributions are similar to that of the human. While bioinformatic analysis has implicated that *Arabidopsis thaliana* C3H2C3-type RING domains (241/469, 51.4%) represent the largest, followed by the C3HC4 type [15]. Independent and recent expansions of the C3HGC3 type within *Drosophila melanogaster*, 6 members of C3HGC3 type all lack themselves alternative splicing isoforms, and only one (NP_648919) could be defined as its putative ortholog (NP_060594) in the human (Table S1). BLAST searches using all members of the C3HGC3 type also retrieved the only ortholog from any other genome, including animals such as the rat and mouse, and plants such as *Arabidopsis thaliana* and *Oryza sativa*. The observations may indicate that expansion of C3HGC3-type RING domain was taxonomically more restricted.

### Analyzing Sequence and Structural Elements of RING Domains

Subsequently, we collected a nonredundant set of the solved 57 RING domains with experimental structural data, and performed multiple sequence and structural alignments for different type RING domains of *Drosophila melanogaster* (Figure S1) and the solved RING domains (Figure S3). Alignment accuracy evaluated using structural information was provided with reliable scores, ranging from the minimum 75 of the C3HC4 type to the maximum 99 of the C3HGC3 type (Table 2). A total of the 4312 pairwise sequence comparisons exhibit a large difference at the level of sequence identity within different types, ranging from 1% to 99% (Table S2). The average percentage identity of pairwise sequence comparison within different types are shown in Table 2, ranging from the minimum 22.4% (the solved U-box) to the maximum 53.7% (C3HGC3-type). With an average of 53.7% sequence identity, independent and recent expansions of the C3HGC3 type within *Drosophila melanogaster* are the most conserved. While the C3HC4, C3H2C3, C4HC3, and U-box types are characterized by poor conservation (sequence identity <30%), the values are comparable with the average percentage identity of the solved RING-E3s (Table 2). Owing to the facts that calculating percentage identity is influenced by: 1) RING domain 40–60 amino acids in size; 2) sequence length (the shorter a pair of sequences, the higher percent identity might be expected by chance); 3) particular structural important residues (e.g., *Cys/His*) in RING domains are conserved; the values of sequence identity are actually low. If we calculated percent identity of sequence pairs by merging all alignments (10) into a comprehensive multiple alignments of 196 RING domain sequences, the percent sequence identity would be very low. Some of them may possess almost undetectable sequence identity but still converge on a common tertiary structure of the RING domain.

The second structural arrangements within different types were represented according to structural data of the solved RING domains (Figure S3). Second structures of RING domains from *Drosophila melanogaster* without experimental structural data were predicted by the Advanced Protein Secondary Structure Prediction (APSSP) program, which indicated that most members have a similar second structural arrangement of the ββα motif (data not shown). Of the solved RING domains, except 1BORA and 2CSZA, all others have a similar secondary structural arrangement of the ββα motif. Antiparallel β-strands are linked by a short loop of 2 to 5 residues, one of which is frequently *Gly/Pro*, which may be attributed to the fact that they both can assume the unusual dihedral-angle conformation required for a tight turn. The central helix connecting the first and second coordination sites of the zinc ions varies in size from several to some ten residues. Considering the obvious differences of secondary structure between 1BORA, 2CSZA, and others, we further analyzed their secondary structures using the nearest neighbor and neural network approach by the APSSP program, indicating that they both have a similar secondary structural arrangement of ββα motif to others (data not shown). Therefore, the secondary structural differences between 1BORA, 2CSZA, and others may result from the experimental conditions used when solving their 3D structures.

Beyond the basic core of the ββα motif, all RING domains have typical long N- and C-terminal loops. In addition, C6H3C2D/C6H2C4 and RING/U-box types are provided with structural extension at the N- and C-terminals. Except in the C6H3C2D type (C6H2C4-type) and RING/U-box, the 8 metal-chelating residues are respectively located at the N-loop, short loop between antiparallel β-strands, α-helix, and C-loop. The positions and properties of the 4th and the 5th metal-chelating residues in these members are changeable depending on the types of RING domains. With a number of additional *Cys/His* residues, members from the C3HC3D and C6H3C2D/C types have been shown to form a third zinc ion-binding site. Most of them have an *Asp* substitution at metal ligand positions 8 or 12 instead of *Cys* residues (Figure S1). Because of factors beyond RING domains and more variability of metal-chelating residue positions and zinc ion-coordinating residue pairs, the third zinc ion of the C3HC3D type was not represented. Unlike RING/non-U-box domains, which are stabilized by zinc ions coordinated by the conserved *Cys/His* residues, RING/U-box scaffold, without the full complement of metal-chelating residues, is probably stabilized by a system of salt-bridges and hydrogen bonds [24]. Based on the previous structural evidence from 2BAY [25], the residues involved in stabilizing the U-box were inferred (shaded grey, Figure S1). LIM and PHD domains also share a similar pattern of *Cys/His* residues, but they fold differently and have not been implicated in ubiquitination [26]. By binding UBC domains or smaller peptide motifs, the RING domain may constitute structural and functional units of fold recognition required for E2-dependent ubiquitination [1].

### Evolutionary Expansion of Domain Architecture of Orthologous RING Proteins

Orthologous proteins are expected to retain function more often than other homologs.

**Figure 1:**
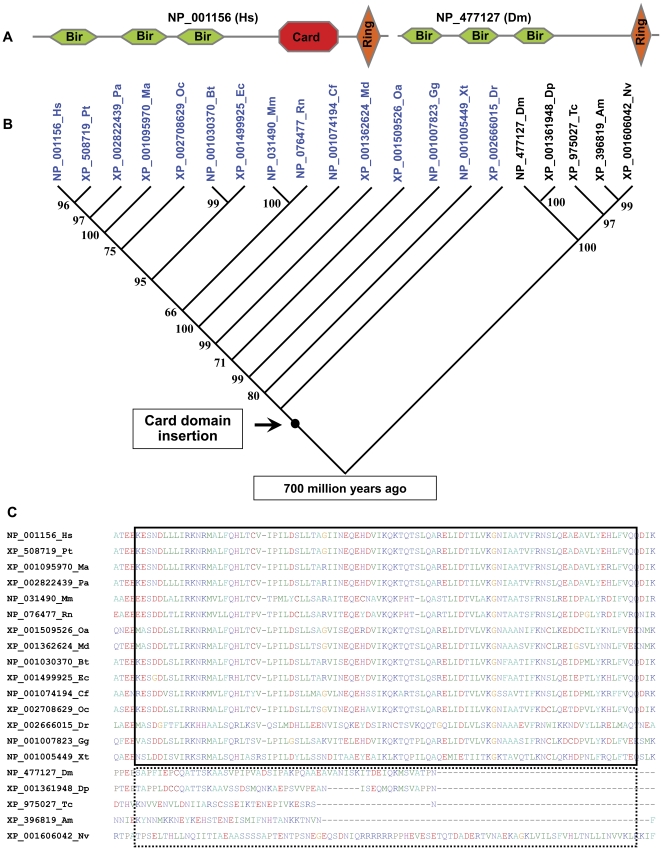
Evolutionary expansion of domain architecture of orthologous Diap2 RING protein. (**A**) Domain architectures of orthologous Iap2/Diap2 from vertebrates and invertebrates were respectively indicated by RING proteins from human (NP_001156) and *Drosophila melanogaster* (NP_477127). Card domains insertion of orthologous Iap2/Diap2 from vertebrates was indicated by red octagon. Hexagons and diamond respectively represented BIR and RING domains. (**B**) Phylogenetic mapping of Card domain insertion/fixation of Iap2/Diap2 orthologs from vertebrates. A black dot marked the time of insertion/fixation in vertebrates. Iap2/Diap2 orthologs from vertebrates were indicated by blue letters. Iap2/Diap2 orthologs from arthropods (invertebrates) were indicated by black letters for easy identification. (**C**) Sequence alignments of Card domain. Sequence alignments of Card domains from vertebrates were indicated by real line rectangle. Alignment of non-Card domains from arthropods (invertebrates) were indicated by dot line rectangle. Hs, *Homo sapiens*; Pt, *Pan troglodytes*; Pa, *Pongo abelii*; Ma, *Macaca mulatta*; Oc, *Oryctolagus cuniculus*; Ec, *Equus caballus*; Bt, *Bos taurus*; Cf, *Canis familiaris*; Mm, *Mus musculus*; Rn, *Rattus norvegicus*; Oa, *Ornithorhynchus anatinus*; Md, *Monodelphis domestica*; Gg, *Gallus gallus*; Xt, *Xenopus tropicalis*; Dr, *Danio rerio*; Dm, *Drosophila melanogaster*; Dp, *Drosophila pseudoobscura*; Am, *Apis mellifera*; Nv, *Nasonia vitripennis*; Tc, *Tribolium castaneum*.

As basic functional units, conserved domain architecture are required for proteins to perform their conserved function. Ari-2/Triad1 contains a N-terminal and a C-terminal RING domains (Table S3), both of them are crucial for binding UbcH7 and Ubc13 to inhibit myeloid cell proliferation [27]. To observe whether a large expansion in domain architecture complexity had arisen among the RING proteins pairs during evolution, we detected such events in orthologous RING protein pairs from *Drosophila melanogaster* and the human, and the results showed that: 1) of the 21 *Drosophila melanogaster* RING proteins without putative orthologs from the human, 15 have the only one RING domain. Considering the data of exonization of Transportable elements, exon duplication and exonization of intronic sequences, we proposed that exonization of intronic sequences contributed mainly to the origin of the RING proteins without putative orthologs from the human after the divergence time of 700 million years ago (Ma) for the vertebrates-arthropods split [28]; 2) of the 118 putative orthologous pairs, 117 pairs (117/118, 99.2%) were found to retain entirely uniform domain architectures. Only domain architectures of Diap2/CG8293 in IAP family experienced evolutionary expansion of domain architecture in the human orthologs (NP_892007) (Figure 1, S4 and Table S3).

*Drosophila melanogaster* IAP family contains 4 members (CG12265, CG12284, CG8293 and CG6303). Of them, two (CG12284 and CG8293) containing RING domain possess E3 activity. Their substrates include molecules involved in apoptosis and signaling, and function in apoptotic and nonapoptotic processes. As building blocks of protein structure, domains can be utilized to recombine in different arrangements to create proteins with different functions. To assess the evolutionary dynamics behind domain architecture of orthologous Iap2/Diap2 from the human, we obtained all the possible orthologs of Iap2/Diap2 from distant phylogenetic lineages, and performed phylogenetic analysis for these orthologous Iap2/Diap2 (Figure 1, S4). Domain architecture comparison of orthologous Iap2/Diap2 showed that: 1) domains of orthologous Iap2/Diap2 from nematode (*Caenorhabditis elegans*) and fungi (*Schizosaccharomyces pombe* and *Magnaporthe oryzae*) are organized by a tandem repeat of 2 BIR domains. Without RING domain, the orthologs from nematode and fungi were omitted in our analysis; 2) all orthologous Iap2/Diap2 from arthropods possess a tandem repeat of 3 BIR domains and 1 RING domain; 3) apart from a tandem repeat of 3 BIR domains and 1 RING domain, all orthologous Iap2/Diap2 from vertebrates acquired an additional Card domain after the divergence time of 700 Ma for the vertebrates-arthropods split [28]. As multifunctional protein, BIR1 interacting with a diverse array of signaling intermediates, and BIR2 and -3 of Iap2 are involved in the binding of caspases and apoptosis-regulatory molecules, and RING domain function as E3 ligase [29]. In inhibiting caspase-9, the third BIR domain is the minimal region of Xiap that is needed for potent caspase-9 inhibition [30]. Card domain functions in apoptosis, cytokine processing, immune defense, and NF-kappaB activation. As to the function of Iap2 Card domain, it is currently unknown. BIR2 of Diap1 functions like BIR3 of Xiap and binds strongly to the IBM-containing *Drosophila melanogaster* molecules Reaper, Grim, Hid, Sickle, and the caspase Dronc [29]. The evolutionary conservation of domain architecture between orthologous pairs of RING proteins from *Drosophila melanogaster* and the human, and the occurrence of large numbers of orthologs between the human and *Drosophila melanogaster*, and the low sequence similarity based on pairwise sequence comparison implicated an ancient common origin of the 118 putative orthologous pairs, and also emphasized the notion that RING proteins have experienced strong selective pressure for conservation throughout eukaryotic evolution.

As structural basis and potential source of functional diversity of RING proteins, additional domains outside RING domain should play important roles in functional diversity, substrate specificity and usability of general RING protein function. Carp2 (NP_001017368), a negative regulator of TNF-induced NF-kappaB activation, by virtue of its phospholipid-binding FYVE/RING domain, Carp-2 localized to endocytic vesicles, where it interacted with internalized TNF-receptor complex, resulting in RIP ubiquitination and degradation [31]. Additional domain analysis of RING proteins showed that: 1) of the 139 RING proteins, members of the RING domains located at N-terminal, C-terminal and middle are respectively 56, 64 and 19. With high percentages of RING proteins with the only one RING domain, 56 RING proteins from *Drosophila melanogaster* (56/139, 40.3%) do not contain any other detectable, previously described domain. However, majority of genomic proteins, more than 80% in metazoa, are multiple domain proteins [32]; 2) 4 proteins (CG31721, CG12218, CG15105 and CG5206 in C3HC4) are TRIM/RBCC proteins defined by the presence of the tripartite motif composed of a RING domain, one or two B-box motifs and a coiled-coil region, and RING-E3 activity of their orthologs have been described in the human [33], [34], [35]. An intact coiled-coil region is necessary and sufficient for TRIM/RBCC protein homo- and hetero-dimerization [36]. While the coiled-coil region of RNF81 (NP_003132) of TRIM/RBCC protein is necessary for its cytoplasmic localization and mediating ubiquitination of cytoplasmic substrate through UBE2D1 [37]; 3) as low-molecular-weight RING proteins (∼100 amino acids in length), all members of C6H3C2D type (CG16982/Roc1a, CG16988/Roc1b, CG8998/Roc2, CG34440/Img) are characterized by possessing the only C-terminal RING domain, and function as assembly subunit of large E3 complexes. For example, the SCF (Skp1-Cullins-F box proteins) is the largest family of E3s that mediate ∼20% ubiquitinated protein substrates for 26S proteasome degradation. The RING component of SCF complex consists of Roc1 and Roc2, both of them are essential for the catalytic activity of SCF [38]; 4) the number of RING proteins with the same type of additional domain over two were shown in Table 4. Most of them (ZnF, BBOX, Sina/Siah, IBR and PHD) are Zinc-binding domains. The widespread occurrence of Zinc-binding domains in these RING proteins may reflect: i) a convenient mechanism of stabilization for small domains in a reducing environment where disulfide bonds do not form readily [39]. And also, as stable motif of a few residues ligating metal ions, Zinc-binding domains may be more favored than others; ii) Znf domains clustered together with RING domain, and may constitute a versatile modular structure required for their common biochemical function of RING proteins by cooperation or independence that contribute to diverse cellular processes. There are many superfamily of ZnF domains, varying in both sequence and structure. They can have different binding specificities, and display considerable versatility in binding modes, even between members of the same class (e.g. some bind DNA, others protein). Of seven ZnF_C3H1-containing RING proteins, six are from C3HC4 and one from C3HC3D (Table S3). ZnF_C3H1-containing Roquin RING-E3s (CG16807) of C3HC3D were found function as RNA-binding proteins, and localization of cytosolic RNA granules implicated in regulating messenger RNA translation and stability [40]. ZnF_UBR1 domain important for the targeting of N-end rule substrates were detected in Ubr1 (NP_777576) and Ubr3 (NP_742067) of C3H2C3 RING proteins. By Ubr1 RING-E3 and the HECT-type Ufd4 E3 interacting, both physically and functionally, and producing a longer substrate-linked polyubiquitin chain, the Ubr1-Ufd4 complexes are more processive in targeting to N-end rule substrates for substrate degradation [41]; 5) 5 Sina/Siah-containing RING proteins were distributed in C3HC4 (4) and C3HC3D (1). In Sina/Siah-containing RING proteins, N-terminal RING domain binds E2, and the remainder C-terminal part is substrate-binding domain. The substrate-binding domain of the Sina/Siah family is structurally highly similar to Traf domain, interacts with a number of proteins, and is involved in TNF-alpha-mediated NFkappaB activation [42]. Of 3 Pex2_Pex12-containing RING proteins in C3HC4, missense mutation in the C-terminal RING domain of PEX2 protein results in a complete defect in the peroxisome targeting signal 1 pathway [43]; 6) most of these additional domains are able to function as adaptor proteins for interactions of protein-protein and assembly of multiprotein complexes. For example, as an adaptor protein for receptor protein-tyrosine kinases, Cbl (NP_005179) positively regulates receptor tyrosine kinase ubiquitination in a manner dependent upon its SH2 and RING domains; 7) domains associated with ubiquitination, such as GIDE, USP8_interact and UBA domains, were also identified. In addition, WWE domain mediating specific protein-protein interactions in ubiquitin and ADP ribose conjugation systems were found in several RING proteins (Table S3).

**Table 4:** Information related to some other type domains in RING proteins.

Additional Domain	Number	Example	Zinc-binding domain	Domain Description
RING	56	CG15104(Topors)	Yes	protein-protein interactions; ubiquitin ligase domain
ZnF	21	CG5841(Mib1)	Yes	Binding DNA, RNA, protein and/or lipid
BBOX	7	CG31721(Trim9)	Yes	Binding DNA, RNA, protein and/or lipid substrates
Sina/Siah	5	CG9949(Sina)	Yes	Function within the sevenless pathway
WD40	5	CG18028(Lt)	No	Coordinating multi-protein complex assemblies
IBR	5	CG5659(Ari-1)	Yes	Occurs between pairs RING fingers
TPR	4	CG5203(Chip)	No	Mediate protein-protein interactions
PHD	4	CG5206(Bonus)	Yes	Protein-protein interacton
BBC	3	CG12218(Mei-P26)	No	Coiled coil region C-terminal to (some) B-Box domains
Pex2_Pex12	3	CG7864	No	Peroxisomal biogenesis

Note: Number means the number of RING proteins with the same type additional domain.

### Analysis of Main-Chain Conformation of the Solved RING Domains

**Figure 2:**
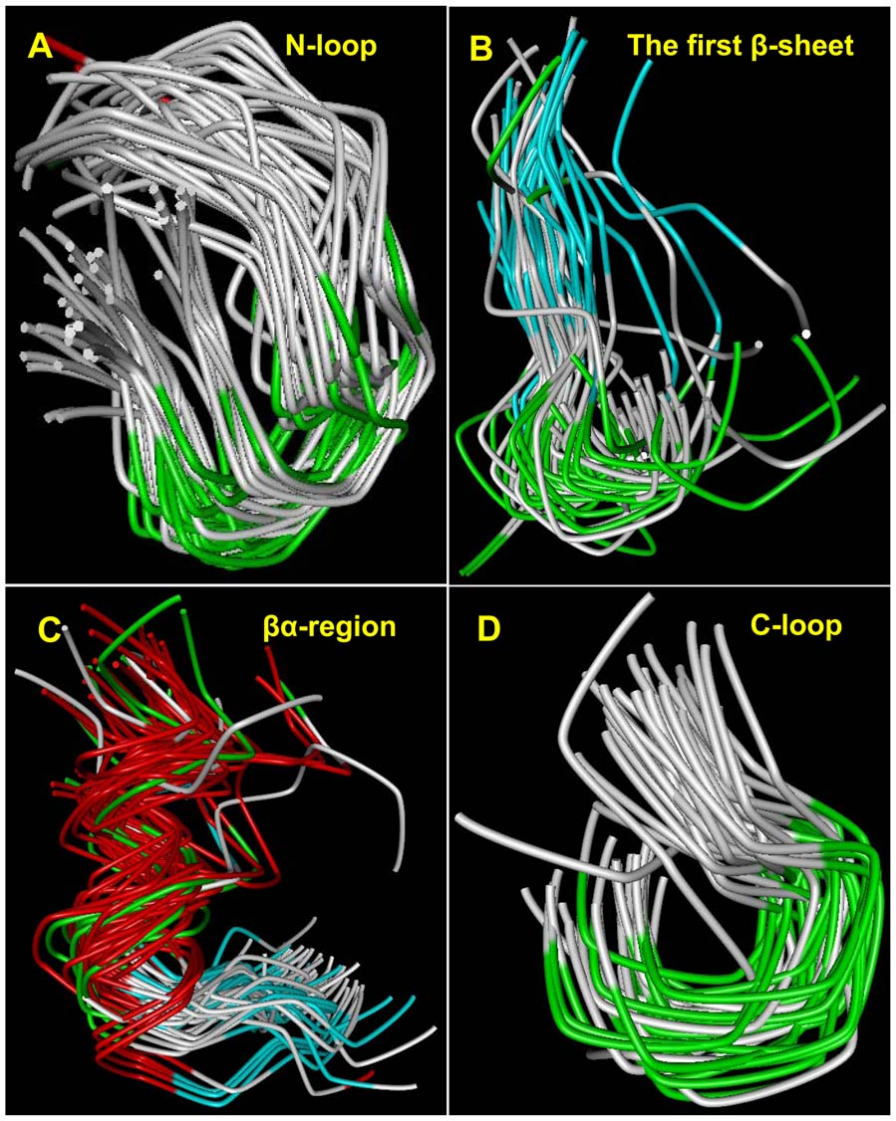
Analysis of main-chain conformation of the solved RING domains. (**A**) N-terminal loop superposed by 9 residues. (**B**) The first β-sheet region superposed by 7 residues. (**C**) βα-region superposed by 13 residues. (**D**) C-terminal loop superposed by 6 residues. N: amino terminal; C: carboxyl terminal; β-sheet: antiparallel β-strands. The backbones of RING domains were superposed by C-Alpha atom in each residue. For details, please refer to Figure S5.

Evidently, both Figure S1 and Figure S3 showed that 4 regions (N-loop, the first β-sheet region, βα-region, and C-loop) close to the conserved *Cys/His* residues are filled in a more orderly fashion than the rest of the RING domains in spite of the noncorrelation of sequences between some of them. To better understand evolutionary-variable and -conserved regions of the RING domains, we analyzed whole and local main-chain conformation using all the solved 3D structures of the RING domains. As a straightforward methodology for detecting structural similarity, pairwise RMSD values were first calculated by the RMSD metric to evaluate their whole structure similarity. With reliable RMSD values (average ≤3.0 Å) for pairwise C-alpha atom superposed by sequence alignments, most of these structures possessed general similarity (Tables 5, S4). All the solved RING domains superposed crystallographic structures are shown in Figure S5A (RING/non-U-box) and Figure S5F (RING/U-box). We then analyzed local conformation and focused on protein segments that conserve a similar main-chain conformation in all the 3D structures. With reliable RMSD values (average ≤2.0 Å), 4 regions (N-loop, the first β-sheet region, βα-region, and C-loop) without insertions and deletions in all solved RING domains were detected to possess similar main-chain conformation [44], are responsible for the homologous folding of RING domains at the superfamily level, and belong to SCRs (Figures 2A–2D, S3, S5; Tables 5, S5). The 4 SCRs mainly responsible for the constitution of this common core of RING domains should be subjected to similar constraints during divergent evolution from a common ancestor. To better understand the determinant roles that the 4 SCRs play in RING domain structure and function, we further analyzed RING domains from sequence and 3D structural perspectives.

**Table 5:** Average of RMSD values for analysis of main-chain conformation.

Type	Average of RMSD values				
	C-alph	N-loop	β region	βα region	C-loop
RING/non-U-box	2.9	0.9	1.64	1.56	0.8
RING/U-box	2.39	0.69	0.88	1.17	0.9

Note: C-alph (RMSD values for pairwise C-Alpha atom superposed by sequence alingments); N-loop (N-terminal loop superposed by 9 residues); β region (the first β-sheet region superposed by 7 residues); βα region (βα-region superposed by 13 residue); C-loop (C-terminal loop superposed by 6 residues).

### Consensus and Conservation of Residues in RING Domains

Different members of the RING domain superfamily display protein folding homolog and functional similarity in spite of low-sequence identity, which may imply that not all of the residues of a RING protein sequence are equally involved in the determination of its structure and function. Sequence information has been extensively used in identifying structural fold, function, and hotspots, and its conservation patterns in homologous proteins are usually functionally important residues [45]. To what extent can we relate RING domain sequence conservation at the superfamily level with structure and function? To get more general information on consensus and conservation of residues in RING domains, we aimed to systematically examine the functionally important residues by evolutionary trace at the superfamily level. Unfortunately, we could not obtain significant results (data not shown), which might be in part due to the low degree of amino acid sequence identity in RING domains. Alternatively, using sequence consensus levels equal to or greater than 0.8 and sequence conservation indices greater than 4 as a scoring rule, we performed a direct sequence comparison to define the extent of consensus and conservation of amino acid sequences of RING domains.

**Figure 3:**
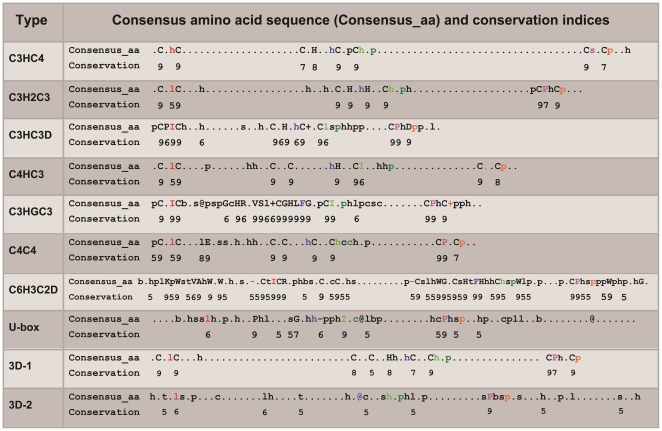
Consensus and conservation of residues identified in sequence alignments of RING domains. Consensus amino acid sequences for positions with a consensus level above 0.8 and conservation indices for positions with a conservation index above 4 were shown (3D-1: the solved RING/non-U-box domains; 3D-2: the solved C6H3C2D and RING/U-box domains). Consensus amino acids of equivalent positions across different types were indicated by the same color letters. Namely, consensus and conservation of 1, 2, 3, 4, 5 and 6 residues in RING domains were respectively indicated by red, blue, light green, deep green, pink and orange letters. The colors of residues in this figure were used throughout our analysis. Consensus amino acid symbols are: conserved amino acids are in bold and uppercase letters; aliphatic (I, V, L): l; aromatic (Y, H, W, F): @; hydrophobic (W, F, Y, M, L, I, V, A, C, T, H): h; alcohol (S, T): o; polar residues (D, E, H, K, N, Q, R, S, T): p; tiny (A, G, C, S): t; small (A, G, C, S, V, N, D, T, P): s; bulky residues (E, F, I, K, L, M, Q, R, W, Y): b; positively charged (K, R, H): +; negatively charged (D, E): −; charged (D, E, K, R, H): c. Four regions (N-loop, the first β-sheet region, βα-region and C-loop) with a mean positional RMSD≤2.0 Å, lacking insertions and deletions were indicated by green dot line frame for easy identification. 1st: N-loop region; 2nd: the first β-sheet region; 3rd: βα-region and 4th: C-loop region.

To obtain general profiles of equivalent residues and positions across different types of RING domains, all alignments (10) were merged into a comprehensive multiple alignment of 196 RING domains. A preliminary comparison of all superfamily members showed that additional residues (except the conserved *Cys/His* residues in RING domains) are unlikely to be a general feature of its superfamily. In other words, the *Cys/His* residues involved in the stability of RING domain structure are the most conserved, which ensures RING domain folding more conservation than its residues' sequences. To infer to what extent consensus and conservation of additional amino acids occurs but not the conserved *Cys/His* residues, we used the above alignments which revealed that 6 residues near the conserved *Cys/His* pairs in the SCRs of the N-loop, βα-region and C-loop have a higher degree of consensus and conservation than others in RING domains. These agree well with residues of conservation in RING Domains from *Arabidopsis thaliana* [15]. Specifically, 4 hydrophobic and 2 polar residues possess good-consensus and -conservation in RING domains; the 6 consensus and conservation of residues at equivalent positions are indicated in color (Figures 3, S1 and S3). The 4 hydrophobic amino acids are respectively located at the N-loop, βα-region and C-loop of the SCRs. The first hydrophobic residues of *Ile* (located at the N-loop SCRs) in the C3HC3D, C3HGC3, and C6H3C2D types; the second hydrophobic residues of *Phe* (located at the βα-region SCRs) in the C3HGC3 and C6H3C2D types; and the third hydrophobic residues of *Ile* (located at the βα-region SCRs) in the C3HGC3 and U-box types are completely conserved. And the fourth hydrophobic residues, located at the C-loop SCRs, are *Pro* in all members from the C3H2C3, C3HC3D, C4C4, C3HGC3, C6H3C2D, and U-box types. The first polar residues, located at the α-helix of the βα-region SCRs, completely differ from each other. They are in positively charged residues such as *Lys* and *Arg* in some members, while replaced by negatively charged residues such as *Glu* or *Asp* in others. The second polar residues, located at the C-loop SCRs, are primarily occupied by *Arg*. Evidently, RING/U-box and RING/non-U-box types not only share consensus and conservation of residues but the residues in the U-box are also located at the proximity of those that stabilize the RING/U-box domain (Figures 3, S1 and S3). Therefore, despite the lack of the conserved *Cys/His* residues of structural importance for the maintenance of the RING domain fold, the RING/U-box were provided with similar basic principles responsible for the maintenance of fold as in the RING/non-U-box.

In addition, most members from C3HC4, C3H2C3, C3HC3D, C4HC3, C4C4, and C6H3C2D types have one *Trp* at the α-helix of the SCRs, which has been shown to be involved in intermolecular interfaces of RING-E3 and E2 pairs for the c-Cbl and UbcH7 complexes [46]. However, as *Glu*49 of 1UR6B (Cont4) at the α-helix has also been shown to directly participate in the intermolecular interfaces for Cont4 and UbcH5B (1UR6) by electrostatic interactions [16], it has a completely different picture in that most members lack the corresponding equivalent residues at the equivalent position. Certain equivalent functional residues exhibit a large difference in the degree of conservation across different types. Similarly, residues at intermolecular interfaces of the different E2 and E3 complexes are also completely different from each other (section “Intermolecular Interface Features of RING-E3 and E2 Complexes”) [47]. On the one hand, due to the joint effects of residue properties and positions, individual equivalent residues and single equivalent positions may not always be as crucial for the interactions of E2/RING-E3 pairs, may rely on the specific environment located by the equivalent residues, and may vary depending on the extent to which E2 is bound by the RING-E3. On the other hand, the plasticity of the protein backbone to some extent influences the differences between equivalent residues and equivalent positions in RING domains while not affecting their functional role as RING-E3s. Simultaneously, the diversity of equivalent residues and positions may contribute to the same RING-E3 association with different E2s in a different context. Undoubtedly, combination of all of these factors greatly increase the difficulty of accurately and efficiently identifying crucial residues from sequences as well as in analyzing the possible interaction between residues of RING-E3 and E2 pairs.

### Common Structural Constraints within the Core of RING Domains

Main-chain conformation and sequence profile highlight the importance of the 4 SCRs and 6 equivalent residues. Do these observations truly reflect common structural constraints found within the core of RING domains at the tertiary structure level during evolution? Are the 4 SCRs and the consensus and conservation of residues in RING domains the critical signal, or just noise for RING domain structure and function? To address these questions, using all the solved RING domains, we first analyzed and compared spatial distribution of the 6 equivalent residues at the SCRs by mapping them onto their 3D structures. The calculation of spatial distance between residues indicated that the hydrophobic residues were clustered in space in close proximity by centering around the second hydrophobic residues at the second β-sheet of the SCRs. Specifically, the minimum spatial distance between any one of them and the second hydrophobic residues is less than 4 Å. Analysis of spatial distribution of the equivalent residues in 3D structures demonstrated that the hydrophobic residues (located at the N- and C-loops), distant from each other in their primary and secondary structures, can finally achieve spatial proximity in 3D structures and convene within a large hydrophobic patch, which is flanked by the 2 polar residues of consensus and conservation.

**Figure 4:**
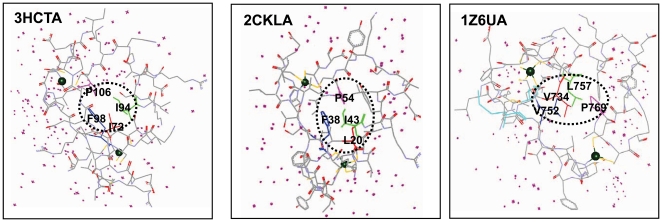
Cartoons of the four hydrophobic residues mediating the formation of the conserved hydrophobic core of solvent inaccessibility randomly chosen from the study samples. RING domains were displayed by linear atom and colord by elements. The conserved hydrophobic core of solvent inaccessibility was indicated by black dot circle. Water was indicated by purple star. 2 zinc ions coordinated by the conserved *cys/his* were indicated by green ball. The four hydrophobic residues formed the core of RING domains were indicated by one-letter amino acid code. Name of RING domains were indicated by PDB ID. Residue numbering referred to their structural data from PDB database.

At the heart of a stable protein domain, are the solvent unexposed residues in its core. Solvent unexposed core residues in its core are known to be key factors that promote the emergence of solvent inaccessibility in interior core and maintain the thermodynamic stability of structural core [48]. Using conserved solvent inaccessibility as a metric [49], we analyzed its conserved solvent inaccessible region and identified core residues constituting hydrophobic core of solvent inaccessibility in RING domains. Data from all the solved RING domains showed that, accompanying the fold of RING domain mediated by the conserved *Cys/His*, the four hydrophobic residues mediate a conserved hydrophobic core packing of solvent inaccessibility. By expulsing water, the four hydrophobic residues are key to the formation of the conserved core of solvent inaccessibility, which appears to be an inherent properties of RING domain 3D structures (randomly selected and represented in Figure 4). The second and the third hydrophobic residues tended to be largely buried in the interior of the conserved hydrophobic core, and contribute to constructing the conserved hydrophobic core of the solvent inaccessibility. This observation is consistent with section “Intermolecular Interface Features of RING-E3 and E2 Complexes”, which indicated that the 2 residues are not involved in the direct binding and interaction with E2s. Therefore, the 2 residues, not essential for direct interaction with E2s, may be important non-functional conserved residues that maintain the active site geometry of the conserved hydrophobic core and the kinetic and equilibrium binding of RING-E3 and E2 complexes.

**Figure 5:**
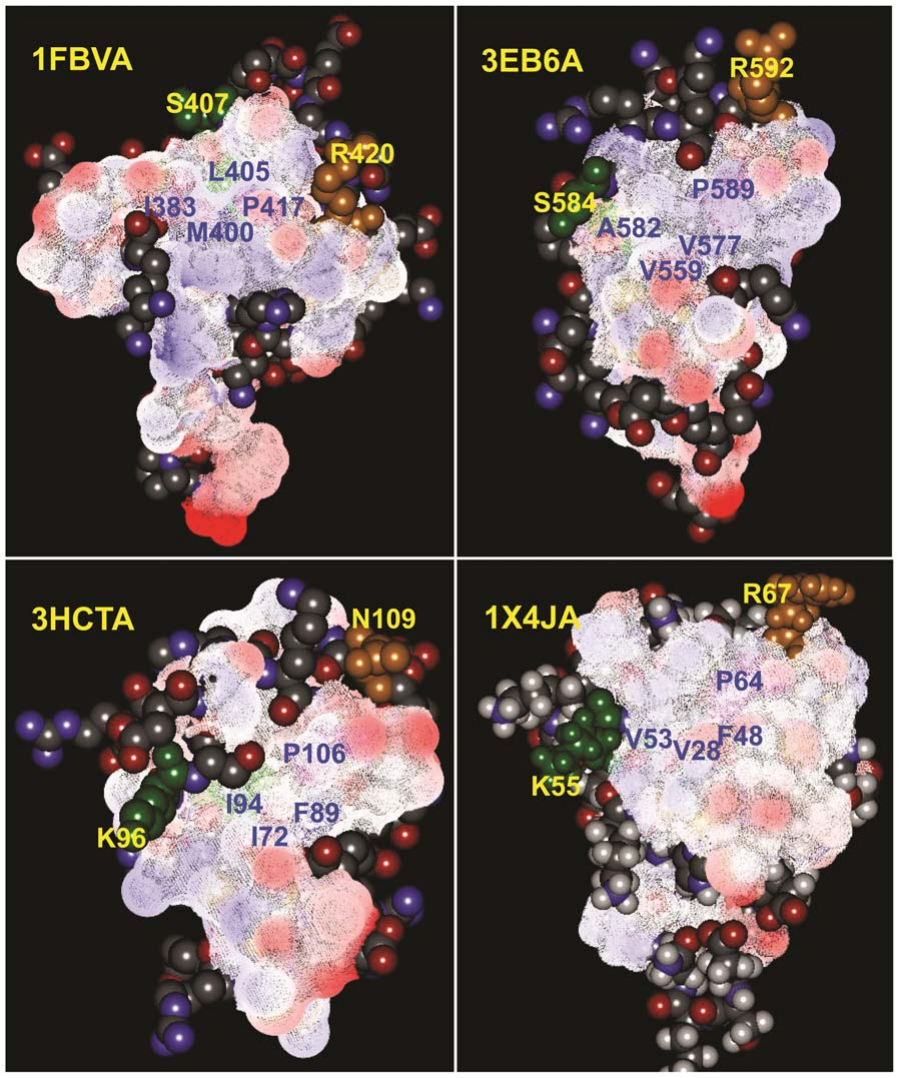
Cartoons of VDW surfaces created for all hydrophobic residues of RING domains. Based on the van der Waals radius (VDW) of each atom in the molecule, VDW surface were created for all hydrophobic residues of RING domains, which were colored by Electrostatic potential. The spatial distribution of consensus and conservation of residues in RING domains were respectively indicated by blue (hydrophobic residues) and yellow letters (polar residues). 3D structures of RING domains were displayed by atom of ball and stick, which were colored by elements. For details, please refer to Figure S6.

In order to visualize the spatial arrangement, we first created a van der Waals (VDW) surface for all hydrophobic residues of the solved RING domains based on the VDW radius of each atom in the molecule, colored by their electrostatic potential (randomly selected and represented in Figures 5 and S6). The unaltered spatial distribution, including sequential relative orientation and position of the residues of consensus and conservation and their stereo-specific assignments may indicate their structural and functional importance for RING-E3 catalysis. Based on the following general notions: 1) protein folding is usually guided by residue interactions that form clusters in the protein core; 2) serving as potential nucleation sites in the folding process, interactions between residues and clusters are governed by the hydrophobic propensities that the residues possess; 3) surface hydrophobicity can be utilized to identify regions on the protein surface most likely to interact with a binding ligand. We then examined all possible hydrophobic residues in spatial proximity to the 4 hydrophobic residues within 4 Å to gain insight into the surrounding properties of the hydrophobic patch using all the solved RING domains (Table S6). We found that besides the 4 hydrophobic residues, there are other hydrophobic residues located on the solvent-accessible surface that are close in space to the second hydrophobic residues (referred to as the central hydrophobic residues, indicated by italics in Table S6) within 4 Å, and are intimately packed to form a conserved hydrophobic patch centering on the central hydrophobic residues by direct and/or indirect VDW contacts.

The central hydrophobic residues occupy a central position within the hydrophobic cluster delimited by the first β-sheet and the central helix. Of the central hydrophobic residues of the solved 57 RING domains, over half (29/57 = 50.9%) are occupied by a large bulky aromatic *Phe* residue. Similarly, of the central hydrophobic residues of the 139 RING domains from *Drosophila melanogaster*, 75 are occupied by the *Phe* residue (75/139 = 54.0%). Substitution of the internal residue *Phe*25 in *Rhodobacter sphaeroides thioredoxin* by 5 amino acids (*Ala, Val, Leu, Ile, Tyr*), of which the aliphatic amino acid substitutions (*Ala, Val, Leu, Ile*) significantly decreased the protein stability that was possible due to loss of extensive VDW contacts that *Phe*25 made with its neighboring residues. The F25Y (*Tyr*) substitution did not evidently affect protein stability, which may be attributed to the similar property of the *Tyr* residue to *Phe* in possessing a large bulky aromatic side chain, that can adopt similar VDW contacts with its neighboring residues as *Phe*25 does [50]. This is supported by the presence of large numbers of *Tyr* residues at the positions of central hydrophobic residues in the solved RING/U-box domains (Figure S3, Table S6). By VDW interactions between the central hydrophobic residue and its spatially close hydrophobic residues and pull towards each other, the central hydrophobic residue may play a key role in guiding the pack of hydrophobic residues to the protein interior. Hydrophobicity of the central residue may be requisite for the formation and maintenance of the conserved core of solvent inaccessibility and the conserved hydrophobic patch of RING domains in E2-dependent ubiquitination. This notion is strengthened by the fact that 2VJEB, with a *Thr* residue at the equivalent position of the central hydrophobic residue, is the only one of the solved RING domains that show serious packing defects in the conserved hydrophobic core (Table S6), which may result from: 1) lack of driving power of the corresponding central hydrophobic residue to spontaneously guide the formation of the hydrophobic surface patch; 2) *Thr* residue contains an aliphatic chain with a hydroxyl group, making it highly reactive and highly hydrophilic.

**Figure 6:**
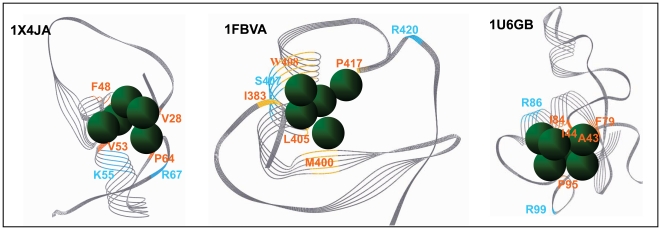
Cartoons of binding pockets of RING domains randomly chosen from the study samples. RING domains were indicated by grey strands. Ligand binding pockets were indicated by green ball. Hydrophobic residues identified in the pockets of RING domains were indicated by orange letters. Polar residues of consensus and conservation identified in the following sections were indicated by blue letters. Name of RING domains were indicated by PDB ID. Residues were indicated by one-letter amino acid code. Residue numbering referred to their structural data from PDB database.

Considering the fact that cavities on a protein surface create the physicochemical properties needed for a protein to perform its function, using the solved RING domain 3D structures, we analyzed surface accessible pockets, as well as interior inaccessible cavities by CASTp [51] (randomly selected and represented in Figure 6); this indicates that most of them have a similar binding pocket, and the 4 SCRs play important roles in the formation of surface accessible pockets as well as interior inaccessible cavities at the superfamily level. The identification of residues participating in the formation of the binding pockets showed that they agree well with the equivalent residues of consensus and conservation. Those that mapped onto the RING protein surface generate hydrophobic clusters, and constitute functional interfaces of E2/RING-E3; others are structurally important residues for the formation of hydrophobic contact to E2s. As shown in RING proteins from *Drosophila melanogaster*, the fourth hydrophobic residues at the C-loop in the solved RING domains were mainly occupied by *Pro* residues, accounting for 50/57 (87.7%). By fixing the main chain dihedral angle at approximately −65±11°, the rigid pyrrolidine ring of *Pro* may play an important role in hydrophobic contact of intermolecular interfaces of RING-E3 and E2 complexes. The second polar residues at the C-loop in the solved RING domains are also overrepresented by the positively charged *Arg* residue (24/57 = 42.1%), which may profit from the following *Arg* residue properties: 1) the largest side chains of the guanidino group attached to the residue contributes significantly to the formation of complexes of E2/RING-E3 by increasing surface contact from a distance; 2) flanking the hydrophobic patch, the positively charged *Arg* residue may contribute to the stability of 3D complexes by electrostatic interactions.

### Subcellular Localization of RING Proteins

Eukaryotic cells are elaborately subdivided into functionally distinct membrane bound compartments. Protein localization tends to be tightly bound to its function, and represents the primary functional information of RING proteins. Subcellular localization analysis of these RING proteins by WoLF PSORT [52] showed that (Table 6, S1): 1) 71 (71/139, 50.1%) RING proteins from *Drosophila melanogaster* were found to be located ≥2 subcellular compartments. As a membrane-associated RING-E3 located multiple subcellular compartments, RNF11 negatively regulate NF-kappaB and jun N-terminal kinase signaling pathways [53]. Several modification and protein interaction signals in the RNF11 sequence are shown to affect its compartmentalization. Membrane binding of RNF11 requires two acylation motifs driving the myristoylation of *Gly*2 and the S-palmitoylation of *Cys*4. RNF11 mutated in the palmitoylation signal is retained in compartments of the early secretory pathway [54]; 2) a significant percentage of RING proteins localized to either nucleus (42/139, 30.2%) or membrane-associated proteins (22/139, 15.8%) (Table S1). RING proteins localized to different nuclear compartments were classified into nuclear proteins, and membrane proteins include nuclear membrane proteins, plasma membrane proteins, and different organelle membrane proteins for easy statistics. Of C3HC4 type, 34 (34/68, 50%) members are located nucleus, while 21 of C3H2C3 type (21/29, 72.4%) are located multiple subcellular compartments (Table 2, S1). As a nuclear protein with multiple nuclear functions, Mkrn1 (NP_038474) inhibited the transcriptional activities of not only c-Jun, but also the nuclear receptors, the androgen receptor, and the retinoic acid receptors. Truncation analysis indicates that both the amino and carboxy termini ZnF_C3H1 domains are required for this transrepression activity and transactivation effects on RNA polymerase II-dependent transcription [55]. As an integral membrane protein of peroxisomes, transmembrane regions of Pex2_Pex12 domain are essential for membrane-anchored in peroxisome biogenesis. 4 transcription factor TFIIE complex (CG3639 and CG4030 in C3HC4, CG9934 and CG6179 in U-box) were predicted to be located multiple subcellular compartments, and translocated into the nucleus in response to phosphorylation. Of them, subcellular distribution of Nosip (orthologs of CG6179) are dynamically regulated by neuronal activity in vitro as well as in vivo [56].

**Table 6:** Predicted locations of RING proteins from *Drosophila melanogaster*.

Location (s)	Nucleus	≥2 localizations	Membrane protein	Mitochondrion
Number	42 (30.2%)	71 (50.1%)	22 (15.8%)	4 (2.9%)

Notes: Multiple localizations include nucleus, cytoplasm and so on. Predicted locations include extracellular space, cytoplasm, nucleus, mitochondria, Golgi apparatus, endoplasmic reticulum, peroxisome, vacuoles, cytoskeleton, nucleoplasm, nucleolus, nuclear matrix and ribosomes.

Domain architecture of orthologous pairs of RING proteins from *Drosophila melanogaster* and the human are evolutionary conserved, which may suggest they have a common core function. Experimental function data of orthologous RING proteins showed that, of 118 orthologous RING proteins, 88 RING-E3 activities have been confirmed by experimental data (Table S3). Gene functions of RING proteins are involved in a variety of biological processes, including vesicle mediated protein sorting, various signaling transduction and transcriptional regulation pathways and so on. As estrogen receptor signaling pathway of nucleus transcription regulator and modulator of DNA demethylation, Rnf4 (NP_002929) located at nucleus and nucleoplasm, has been shown to interact with, and inhibit the activity of Trps1, a transcription suppressor of GATA-mediated transcription [57]. Traf6 located multiple subcellular compartments is versatile, mediating signaling not only from the members of the TNF receptor superfamily, but also from the members of the Toll/IL-1 family. Signals from receptors such as CD40, TNFSF11/RANCE and IL-1 have been shown to be mediated by it. Traf6 also interacts with various protein kinases including IRAK1/IRAK, SRC and PKCzeta, which provides a link between distinct signaling pathways. In addition, RNF13 (NP_009213), an integral membrane-associated RING-E3, is targeted to the inner nuclear membrane through recycling endosomes, and has the potential to turn over key nuclear proteins in response to signals received at the plasma membrane [58].

### Interolog Interactions of RING-E3 and E2 Pairs

Mapping human E2/E3-RING interactions have provided us a detailed, genome-wide overview of binary E2/E3-RING interactions in human ubiquitination system [17], [59]. Based on the existing information about interolog interactions of RING-E3 and E2 pairs, we further mapped interolog interactions of RING-E3 and E2 pairs from *Drosophila melanogaster* (Table S7). Data analysis showed that: 1) of the 118 orthologous RING proteins of *Drosophila melanogaster*, 46 can be identified the putative interolog interactions of RING-E3 and E2 pairs (46/118, 39.0%). And CG15104 (Topors) and CG1134 (Mul1) in C3HC4, and CG3929 (Deltex) in C3H2C3 seem to display broader E2s binding profiles than others in *Drosophila melanogaster* ubiquitination system. Topors functions not only in vitro as a RING-dependent E3 ubiquitin ligase with the E2 enzymes UBE2D1-3 and UBE2E1 for p53 [60], but also as a RING-independent SUMO-1 E3 ligase with UBE2I for p53 [61]. In addition, by catalyzing the assembly of a lys63-linked polyubiquitin chain, Ubc13-Uev1A and Traf6 play a non-proteolytic role of ISGylation in the NF-kappaB pathway of negative regulation [62]; 2) quite a numebr of RING-E3s can bind both class I and non-class I E2s. For example, RING-E3 CG2679 (Goliath) can bind to UBE2D1-4 (class I E2s), UBE2E1, UBE2E3, UBE2N (class II E2s), and UBE2Z (Class IV E2s). By interaction with different E2s, the same RING-E3s can mediate ubiquitination/ubiquitination-like modification of different substrates. RNF81 (NP_003132) has both cytoplasmic and nuclear substrates, and mediates ubiquitination through UBE2D1 in the cytoplasm and through UBE2E1 in the nucleus [37]; 3) similar to human ubiquitination system, members of the UBE2D (class I E2s), UBE2E (class II E2s) and UBE2U (class III E2s) families seem to show much broader RING-E3s binding profiles than other E2s. UBE2D1, UBE2D2, UBE2D3 and UBE2D4 are highly similar, and usually bind to the same RING-E3s (Table S8). UBE2D are the most active class of E2 enzymes in cell extracts and are associated with regulation of a number of transcription factors; 4) except that most of RING-E3s binding to UBE2E1 and UBE2E3 are from C3H2C3 type, RING-E3s binding to the other E2s are mostly from C3HC4-type (Table S8); 5) the same RING protein can participate in ubiquitination and ubiquitination-like modification for antagonistic, synergistic or multiple outcomes. For example, Ari2 and Ubox5 RING-E3s participating in ISGylation, Mul1 RING-E3 in SUMOylation, and Rbx RING-E3s in NEDDylation also exhibit broad ubiquitination activity [63], [64]. The observations highlighted the facts that ubiquitin pathways and ubiquitin-like pathways are overlapped not only by sharing the common E2s, but also the common RING-E3s.

**Figure 7:**
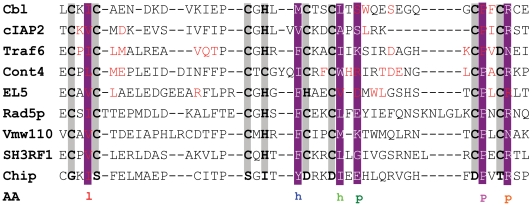
Key residues with experimental data in RING-E3 for E2/RING-E3 interactions. Consensus amino acids were showed by pansy for easy identification. The conserved metal ligand position and residues involved in coordinating Zinc ions were shadowed by grey. Key residues of RING-E3 with experimental evidence in these RING-E3s were indicated by red letters.

To illustrate how an E2 can accommodate different RING-E3s, previously, experimental analysis of key residues of RING-E3 have been conducted in Cbl, cIAP2, Traf6, Cont4, El5, Rad5p, Vmw110, Sh3rf1 and Chip RING-E3s (Figure 7). Despite limited experimental data, these offer direct evidence in favor of the current work that the first and the fourth hydrophobic residues, and the first and the second polar residues of the 6 equivalent residues identified in the above are key residues in some of them. As to the second and the third hydrophobic residues, we are unable to find experimental data for their directly participating in E2/RING-E3 interactions. The observations are consistent with the following section, which indicated that the 2 residues do not directly participate in E2/RING-E3 interactions, may be important non-functional conserved residues that maintain the active site geometry of the conserved hydrophobic core of RING-E3s. The preference of RING E3s for their cognate E2s vary in different RING-E3 and E2 pairs [47]. The nature of corresponding residues involved in E2/RING-E3 interaction varies in a correlated fashion in different E2-RING E3 pairs. For example, in c-Cbl and UbcH7 pairs, *Ile*383 and *Trp*408 of c-Cbl and the UbcH7 *Phe*63 have a central role in determining the specificity of the c-Cbl E3 for the E2 [47]. While, in the Rad6 and Rad18 E2-RING E3 pairs, the residue corresponding to the UbcH7 *Phe*63 is *Asn*65, and the residue corresponding to the c-Cbl *Trp*408 is *His*55 [47]. Similarly, in Ubc9-Pml pairs the residues corresponding to *Phe*63 of UbcH7 and *Trp*408 and *Ile*383 of c-Cbl are replaced by *Ser*70, *Gln*59, and *Ser*84 respectively [65].

### Intermolecular Interface Features of RING-E3 and E2 Complexes

Most rapid progress has been achieved in functional studies of interolog interactions of RING-E3 and E2 pairs [17], [59], while the molecular mechanisms underlying its function are still poorly understood. Do the SCRs, the sequence conservation patterns, and the common structural core truly reflect their importance in direct interactions of the intermolecular interfaces of 3D complexes during evolution? Hydrophobic residues and specific charge distributions have been shown to be characteristic of intermolecular interfaces [66]. To obtain a comprehensive knowledge about the intermolecular interfaces of E2/RING-E3 pairs and the principles governing the interactions of E2/RING-E3 pairs recognition and binding, we performed the following steps: 1) using all the cocrystal structures of E2/RING-E3 pairs collected with experimental 3D structures as templates, the 3D models of respective orthologs of RING-E3s and E2s from *Drosophila melanogaster* were respectively constructed by homology modeling approaches; 2) using the RMSD metric, we measured the mean distance between the corresponding atoms in the 2 structures after targets and templates were superimposed by sequence alignment using C-alpha in each residue; 3) with quite reliable RMSD values (0.05 to 0.984), we obtained high-quality modeled complexes of E2/RING-E3 pairs (detailed evaluation of models and RMSD calculation are listed in Table 7); 4) ultimately, seven 3D complexes modeled by homology modeling were obtained by structural replacement of the coordinates of the respective template (Figure S7). Both the evaluation of the structural model and superimposition of targets and templates indicated that targets and templates have a better fit. The close overlay between templates and targets may suggest that not only sequence homology but also ortholog structures are evolutionary conserved, and functions relevant.

**Table 7:** Information related to structural models and its evaluation.

Domain	Target		PMDB ID	Template	ID%	Energy(KJ/mol)	RMSD
	AC	Name					
C3HC4	CG7037	Cbl	PM0076299	1FBVA	74.0	−4720.055	0.318
	CG5788	UbcD10	PM0076300	1FBVC	74.0	−5714.233	0.092
	CG8293	Iap2	PM0076301	3EB6A	59.4	−2046.047	0.096
	CG7425	Eff	PM0076302	3EB6B	93.3	−6994.851	0.073
C3H2C3	CG9381	Mura	PM0076303	1X4JA	88.4	−2939.742	0.05
	CG18319	Ben	PM0076304	3HCTB	79.5	−7847.191	0.07
C3HC3D	CG10961	Traf6	PM0076305	3HCTA	37.3	−4673.560	0.984
	CG18319	Ben	PM0076304	3HCTB	79.5	−7847.191	0.07
C4C4	CG31716	Cont4	PM0076307	1UR6B	88.5	−2034.211	0.069
	CG7425	Eff	PM0076306	1UR6A	94.6	−7110.266	0.061
U-box	CG5203	Chip	PM0076309	2OXQC	79.2	−2279.699	0.089
	CG7425	Eff	PM0076308	2OXQA	92.5	−7173.200	0.104
	CG5203	Chip	PM0076311	2C2VT	73.1	−1820.439	0.64
	CG18319	Ben	PM0076310	2C2VB	78.2	−6734.408	0.33

Note: AC, Gene Accession number of FlyBase; Name, Gene name; PMDB ID, Accession number of strucrural model of PMDB database; Template, the template for homology modeling; ID%, Percentage of sequence identity between target and template; Energy, Final Total Energy; RMSD, Root Mean Square Deviation.

**Figure 8:**
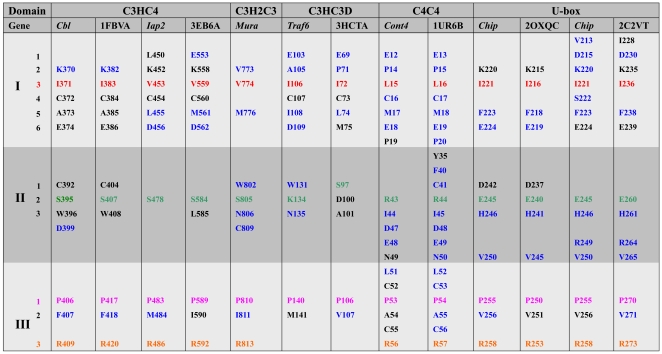
Contact resides of RING domains with E2s in the intermolecular interfaces of their 3Ds complexes. Gene name of target proteins (seven) from fruit fly were indicated by italic letters. Gene names of template proteins (six) were indicated by PDB IDs. On the whole, positions of contact resides in RING domains with E2s are equivalent to each other, which are all located at the SCRs of RING domains. I, II and III respectively respresent N-loop, the α-helix of αβ-region and C-loop of the SCRs. Black letter resides indicated interacting distance cut-off values of 4 Å between appropriate RING-E3 and E2 atoms in their 3Ds complexes. Colourful letters indicated interacting residues (close approach) distance cut-off values within 3.5 Å. 1, 4, 5 and 6 consensus and conservation of residues in RING domains identified in the above alingments were respectively indicated by red, deep green, pink and orange letters. 1 and 5 are hydrophobic residues within the large hydrophobic patchs. 4 and 6 are polar residues flanked at the patchs.

Given a detailed 3D structures of protein-protein complexes, it is possible to specifically and accurately identify the residues crucial for binding [45]. Using 13 3D complexes of E2/RING-E3 pairs, including the solved 6 and the modeled 7 complexes, we investigated the intermolecular interface features occurring within these 3D structures by deducing interaction residues of their intermolecular interfaces (Figure 8), which allow us to draw generalizations and distinctions of their intermolecular interfaces: 1) residues (black font indicating the minimum spatial distance of residues to be within 4 Å; colored font indicating the minimum spatial distance of residues to be within 3.5 Å) directly interacting are all from the N-loop, the α-helix of the αβ-region, and the C-loop of the SCRs in RING domains; 2) in the N-loop SCRs, most complexes include 6 residues (1–6) that directly participate in intermolecular interface contact of RING-E3 and E2 pairs. Of these, residues 3, 4, and 5 have good-consensus in these RING-E3s. Residue 3 is the first hydrophobic residue of consensus and conservation identified in the above alignments (red font, Figures 3 and 8), and residue 4 is the conserved *Cys* chelating zinc ion. Residues 1, 2, and 6 are radically distinct; for example, residue 2 in Cbl (1FBVA) is a bulky positively charged *Lys* (K370), and is replaced by the small hydrophobic residue *Pro* (P71) for the RING domain from Traf6 (3HCTA); 3) in the α-helix of the αβ-region SCRs, most complexes include residues 1, 2, and 3 that directly participate in E2/RING-E3 interactions. Of the 3 residues, the polar residues demonstrate consensus and conservation in the above alignments (green font, Figures 3 and 8), and its equivalent positions are allowed a little discrepancy within the α-helix among different complexes. As to the other 2 in the SCRs, their properties radically differ, such as the bulky hydrophobic amino acids of *Trp* in Cbl (1FBVA: W408), replaced by a polar residue of *His* in Chip (2OXQC: H241). These observations are taken together with the following 2 facts: a) the positions and properties of the 4th and fifth metal-chelating residues in the SCRs are changeable depending to some extent on the types of RING domains; b) RMSD values of the βα-region SCRs showed more difference than others (Table S5), emphasizing the importance of different SCRs needed by diversity and specificity of RING-E3 catalysis; and 4) in the C-loop SCRs, most complexes include residues 1, 2, and 3 in RING domains directly participating in the interactions of E2/RING-E3. Of the 3 residues, 1 and 2 are all hydrophobic amino acids, and 3 is a polar residue. Residues 1 and 3 have consensus and 8). Similarly, residue 1 is mainly occupied by hydrophobic*Pro* residues, while residue 3 is mainly occupied by positively charged *Arg* residues.

The number of E3 RING residues within 3.5 Å distance of the E2 interaction surface is about 10 in most 3D complexes of E2/RING-E3 pairs. Sometimes, the number of difference is very large, such as 1FBVA (6 residues) and 1UR6B (20 residues) (Figure 8). And this may in part explain the observations that affinity and stability of 3D complexes of different E2/RING-E3 pairs largely differ from one to another. Of the 6 consensus and conservation of residues in RING domains, residues 2 and 3 (blue and emerald font, Figures 3 and 8) are not associated with direct contact with E2s in intermolecular interfaces of E2/RING-E3 pairs, and contribute to constructing the active site or the conserved hydrophobic core docking to E2s. While residue 1 (hydrophobic, in the N-loop SCRs), 4 (polar, in the βα-region SCRs), 5 (hydrophobic), and 6 (polar, in the C-loop SCRs) are directly involved in intermolecular interface interactions of E2/RING-E3 complexes.

**Figure 9:**
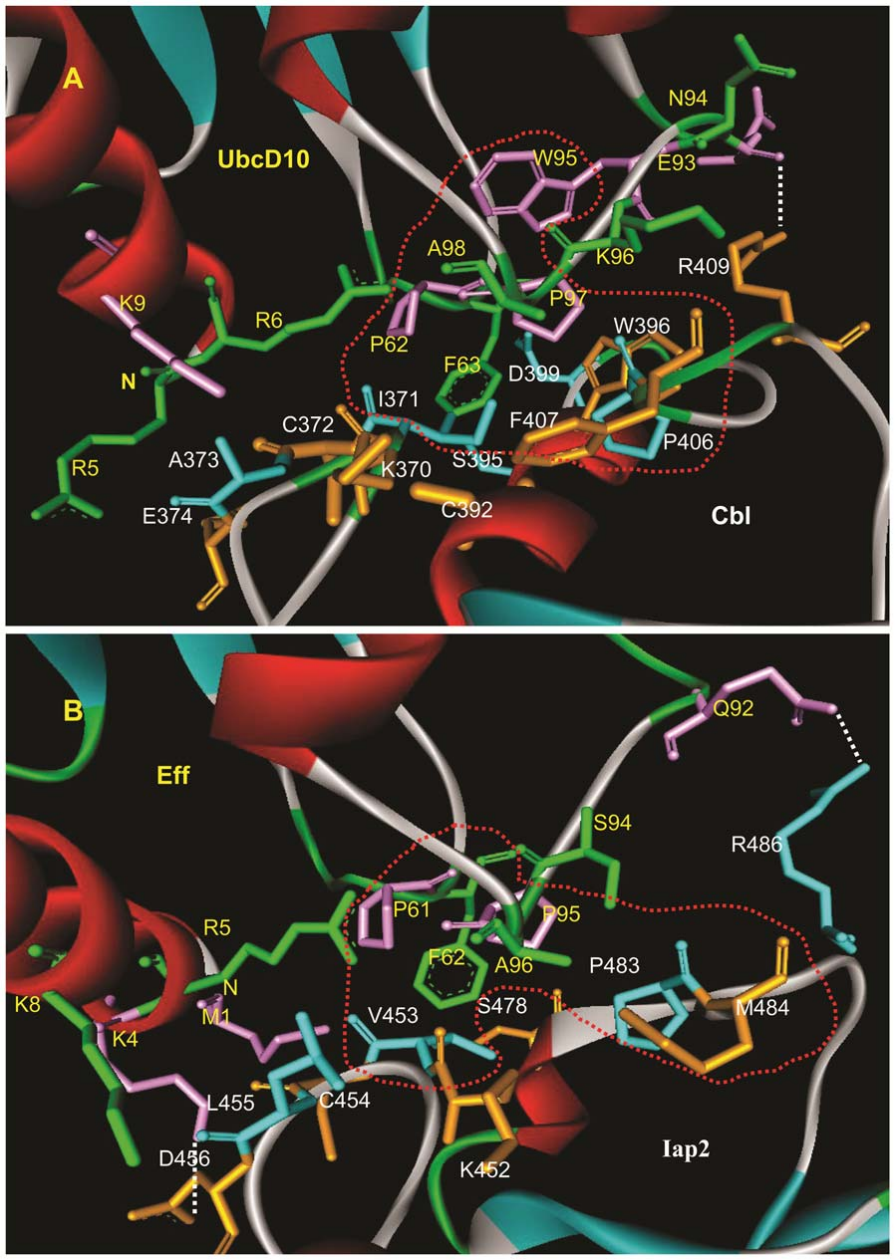
Close-up view of interaction residues in the intermolecular interfaces of 3D complexes of RING-E3 and E2 pairs. **A:** Cbl-UbcD10; **B:** Iap2-Eff. The side chains of 3D complexes of RING-E3 and E2 pairs involved in their interactions were presented by solid ribbon. Resides that make significant directly contacts observed in the modeling complexes were presented by stick model, and were numbered by precursor peptides. The numbers for all resides in the figure correspond to those in the text and the tables. The interaction residues in the intermolecular interface were respectively indicated by yellow (E2s) and white (RING-E3s) letters. The conserved hydrophobic contacts of intermolecular interfaces observed in the modeling complexes were highlighted by red dot circles. Hydrogen bonds of intermolecular interfaces formed by carbonyl-group oxygen and amino-group hydrogen were showed by white dot lines. For details, please refer to Figure S7 and Figure S8.

For visualization of spatial distribution of interacting residues in intermolecular interfaces, we mapped them onto their 3D complexes (Residues of RING domains direct contact with E2s in their 3D complexes are indicated in white font, Figures 9, S7 and S8), which indicated that the E2-binding sites are centered on the CHCs (red circles, Figures 9, S7 and S8), passing between the 2 zinc ion binding loops [46]. The SCRs are responsible for the appropriate positioning of key residues as structural determinants for binding E2s. And the invariant association of the spatial distribution of the equivalent residues of consensus and conservation may represent the result of convergent evolution and may be important elements involved in RING-E3 catalysis. The CHCs are mainly composed of hydrophobic amino acids (e.g. *pro*, *ile*, *val*) from the SCRs. Of the 6 consensus and conservation of residues, residues 2 and 3, as structural conserved residues, contribute to constructing the hydrophobic conserved core of solvent inaccessibility in the CHCs. Residues 1 and 5 directly participate in interactions within the CHCs of intermolecular dynamic surfaces. (Figures 9, S7 and S8). Polar residues 4 and 6 flanking at the CHCs also participate in the direct contact of RING-E3 and E2 pairs by electrostatic interactions. Clustered together on the surface of RING domains and exposed to solvent, the hydrophobic residues in the hydrophobic patch may be forced to pack into CHCs with E2s under the cooperation of hydrophobic interaction and other intermolecular forces such as hydrogen bonds and/or electrostatic interactions [67]. In addition, the hydrogen bonds formed by carbonyl-group oxygen and amino-group hydrogen also contribute to stabilizing dynamic interface by flanking at the hydrophobic cores (white broken lines, Figures 9, S7 and S8). These 3D complexes of E2/RING-E3 pairs are provided with similar intermolecular dynamic surfaces by hydrophobic force, hydrogen bonds, and electrostatic interaction but the differences of structures and residue properties are also evident. However, the differences are unable to influence their common structural and functional characteristics. For example, in spite of the structural differences between K3 and TRAF6, as RING-E3s they both interact with UBE2N [68].

Clearly, the intermolecular interfaces of 3D complexes exhibit conserved and diversified features. From an evolutionary perspective, conservation and consensus of residues may be essential for mediating the formation and maintaining the stability of the hydrophobic conserved core of solvent inaccessibility, and offering the CHCs flat for RING-E3s and E2s recognizing and binding underlying catalysis, while diversification of individual residues of their dynamic interface should be necessary for selective targeting to different E2s. From a structural perspective, the invariant equivalent positions of interacting residues across different RING-E3s suggests that: 1) the integrity and the precise relative arrangement of conserved residues and structure are important for RING-E3 function; 2) despite poor conservation of interaction residues in the various complexes of E2/RING-E3 pairs, their roles in direct structural or functional effects on E2s or potentially allowing conformational changes during catalysis by hydrophobic and electrostatic interactions or hydrogen bonds are common; 3) variable sequences of RING domains likely determine their selective binding properties because each RING domain interacts with a subset of available E2 proteins; the importance of individual residues in RING-E3s can vary from each other depending on different E2/RING-E3 pairs [69]. For example, RING/U-box protein CHIP (NP_005852) can bind to UBE2D2 and UBE2N and promote E2-dependent ubiquitination degradation [70], [71]; but the residues of CHIP interacting with UBE2D2 and UBE2N exhibit a clear difference (Figure 8). For example, the negatively charged *Asp* of position 230 (D230) from the RING/U-box domain of CHIP produces electrostatic interaction with the positively charged *Lys* of position 5 (K5) UBE2N (2C2VT) [71]; but the electrostatic interactions immediately disappear when CHIP interacts with UBE2D2 (2OXQC) [69].

### Conclusion

The present investigation was facilitated by the readily availability of the solved RING domains, the cocrystal structural complexes of E2/RING-E3 pairs and genome sequences, and the findings of previous studies [72], [73]. By extracting information of sequence, structure, and function, the current work offered a clue for better understanding molecular link between structural conservation and diversification and functional similarity and specificity of RING domains, which underlined the common structural determinant of RING-E3 in its catalysis and the general principles governing the interactions involved in recognition and binding of E2/RING-E3 pairs. By detecting evolutionary expansion events of domain architecture of orthologous RING proteins, analyzing subcellular localization of RING proteins and mapping interolog interactions of RING-E3 and E2 pairs, the observations offered a window into understanding orthologous core functions, the essence of what constitutes an active RING domain, and that the key loss-of-function mutation resides of RING-E3. Certainly, the results are derived from a relatively small 3D structural data set of RING domains and cocrystal structural complexes of E2/RING-E3 pairs, and need to be enlarged. Although only a limited number of E2/RING-E3 complexes have been reported, the key common features underlying binding of E2 are becoming apparent [69]. The nature of corresponding residues involved in E2/E3 interaction varies in a correlated fashion in different E2-RING E3 pairs [47]. With rapid growth of 3D structural data, we would like to include more 3D structures of RING domains and cocrystal complexes of E2/RING-E3 pairs, which will especially become significance when the availability of large number of genome sequences, the solved RING 3D structures and the cocrystal complexes. It is also important and urgent to prevent bias of the data set. With the technique establishment of the artificial microRNAs in *Drosophila* [74], the data presented here will certainly be a useful resource to drive future targeted investigations into E3 RING function.

## Materials and Methods

### Database Searches

RING proteins from *Drosophila melanogaster* were comprehensively retrieved from GenBank (http://www.ncbi.nlm.nih.gov/), Uniprot (http://www.uniprot.org/), Supfamily (http://supfam.cs.bris.ac.uk/), InterPro (http://www.ebi.ac.uk/Tools/) and FlyBase (http://flybase.org/) databases. Under the default profile inclusion expectation (e) value threshold, profile searches were conducted using the Position-Specific Iterated BLAST (PSI-BLAST) program with either single RING domain sequence or multiple alignments as queries, which were iterated until convergence. Under the default expected threshold parameters, several online tools, including the Simple Modular Architecture Research Tool 6 (http://smart.embl-heidelberg.de/), CD-search (http://www.ncbi.nlm.nih.gov/), the ScanProsite Proteomics Server (www.expasy.org), the Protein Families database (Pfam 24.0), and the European Bioinformatics Institute (http://www.ebi.ac), were used to analyze and confirm each potential RING domain, and proteins containing RING domains were retained for further investigation. Putative orthologs of RING proteins between *Drosophila melanogaster* and the human were further defined by Reciprocal Best Blast Hits (RBBHs) [20]. Based on uniquely shared sequence patterns of the conserved residue-binding zinc ions and distinct structural features, the complete set of RING proteins detected from *Drosophila melanogaster* were clustered and subdivided into eight types. Multiple amino acid sequence alignments of these types were performed by the Tcoffee-regular program under Expresso (3DCoffee) computation mode [75] and Promals3D program under default parameters [76], followed by manual adjustment according to the conserved *cys/his* residue positions. Simultaneity and confidence level of sequence alignments were evaluated using structural information by Expresso and Promals3D, which construct alignments using structural information from sequence database searches, secondary structure prediction, available homologs with 3D structures, and user-defined constraints.

### Structural Similarity Search

Using either single RING domain sequence or multiple alignments as queries, an initial search for all nonredundant members of RING domains with experimental structural data were iteratively carried out with the PSI-BLAST in PDB database (http://www.wwpdb.org/), until no new sequences were found under the e-value threshold. To identify additional RING proteins of the solved 3D structures with similar topology, structure similarity searches were conducted by DaliLite (version 3.0), which uses Heuristic filters to rapidly find a close neighbor of the queried structure [77]. All hits for each query were collected when similarities were found with a z-score greater than 2, and then parsed for topological congruence to the RING-E3 structural template using a custom PERL script. To assess topological congruence, coordinates of the matching regions detected by DaliLite searches were extracted and analyzed for secondary structure using APSSP. Multiple sequences and structural alignments of all solved RING domains were carried out in like manner, followed by manual adjustment based on their own secondary structure information and the conserved cys/his residue positions; secondary structures were assigned according to structural data.

### Analyzing Domain Architecture of Orthologous RING Proteins

Domain architecture of RING proteins of the 118 putative orthologous pairs from *Drosophila melanogaster* and the human were analyzed by NCBI Conserved Domain Architecture Retrieval Tool, SMART (http://smart.embl-heidelberg.de/) and ExPASy InterPro Scan [78]. Evolutionary dynamics behind domain architecture expansion of orthologous Iap2/Diap2 from *Drosophila melanogaster* and the human were evaluated. All the possible orthologs of Iap2/Diap2 were obtained from distant phylogenetic lineages. Amino acid of all these orthologous Iap2/Diap2 were further analyzed by multi-sequence alignment using Expresso, which automatically incorporate structural information in multiple sequence alignments using 3D-Coffee [75]. Phylogenetic trees were constructed for analyzing evolutionary dynamics behind domain architecture expansion of orthologous Iap2/Diap2 using the program Molecular Evolutionary Genetics Analysis (MEGA) package version 5 [79]. The evolutionary analysis was inferred using Neighbor-Joining method [80]. To assess the reliability of the phylogenetic tree, bootstrap test (3000 replicates; random seed = 50000) were conducted. The evolutionary distances were computed under the model of JTT with Freqs. and are in the units of the number of amino acid substitutions per site. All sites containing alignment gaps and missing-information were retained initially, excluding them as necessary using the complete deletion option. Substitution patterns among lineages were allowed to vary among sites using Gamma distributed with invariant sites [G+1].

The investigated species include human (*Homo sapiens*, Build 37.2), chimpanzee (*Pan troglodytes*, Build 2.1), Sumatran orangutan (*Pongo abelii*, Build 1.2), rhesus (*Macaca mulatta*, Build 1.2), mouse (*Mus musculus*, Build 37.2), rat (*Rattus norvegicus*, RGSC v3.4), cow (*Bos Taurus*, bosTau4), dog (*Canis familiaris*, Build 2.1), cattle (Bos taurus, Btau_4.0), horse (*Equus caballus*, EquCab2.0), rabbit (*Oryctolagus cuniculus*, OryCun2.0), duck-billed platypus (*Ornithorhynchus anatinus*, Build 1.1), opossum (*Monodelphis domestica*, MonDom5) chicken (*Gallus gallus*, Build 2.1), zebrafish (*Danio rerio*, Zv8), western clawed frog (*Xenopus tropicalis*, Build 1.1), fruit fly (*Drosophila melanogaster*, *Drosophila pseudoobscura*, FB2011_03), honey bee (*Apis mellifera*, Amel_4.0), jewel wasp (*Nasonia vitripennis*, Build 1.1), red flour beetle (*Tribolium castaneum*, Build 2.1), nematode (*Caenorhabditis elegans*, W198), fission yeast (Schizosaccharomyces pombe, Build 1.1), rice blast fungus (Magnaporthe oryzae, Build 3.1). In additional, WormBase, FlyBase, VectorBase, SGD, DictyBase, *M. brevicollis*, v1.0 and Gramene Homepages were also retrieved by basic local alignment search tool (BLAST) (see reference database).

### Analysis of SCRs in RING Domains

A central tenet of structural biology is that related proteins of common function share structural similarity. To identify the SCRs of RING domains, we first inspected the superposability of the solved RING domain under a threshold of RMSD [81]. Multiple structural alignments and superpositions of the solved RING domain were utilized to identify the common core and the SCRs across members of the superfamily. SCRs were defined as regions displaying similar local conformation, with a mean positional RMSD of the equivalent C-alpha atom positions of every structure superposed less than or equal to 3.0 Angstroms (Å) [82], lacking indels (insertions and deletions) in all structures considered and composed of at least 3 consecutive residues. A C-language routine was developed to extract from the 3D coordinates of the superimposed structures and the related multiple alignments of the candidate SCRs. For every structurally equivalent position of the multiple alignments, the RMSD from the center of mass of the structurally equivalent C-alpha atom was calculated. In avoidance of the occurrence of SCRs with indels, positions with gaps were not considered. A window (size *w* = 3) position was then scrolled through the alignment and used to define seed positions with a mean RMSD≤3.0 Å in JavaScript. Once a seed position was found, *w* was iteratively increased by one position consecutively until the mean score did not rise above 3.0 Å, or until the window reached the end of the multiple alignments. In addition, the solved RING domain 3D structures were used to calculate the fraction of hydrophobic residues it encapsulates along a sequence by Scooby-Domain [83]. This leads to a 2D matrix, and the matrix values are converted to probability scores by referring to the observed distribution of hydrophobic residues. In addition, Castp (http://sts.bioengr.uic.edu/castp/) were also used in the data analysis.

### Comparative Modeling

The constructions of 3D models were performed through the dedicated server SWISS-MODEL workspace (http://swissmodel.expasy.org/), whose 4 tools (template identification, sequence feature scan, structural assessment, and SwissModel template library) were utilized. In addition, the PDB database was searched for template identification. For the computational simulation of 3D complexes of RING-E3s docking to E2s from *Drosophila melanogaster*, we first obtained all the cocrystal structures of RING-E3 and E2 pairs with experimental structure data, including 6 cocrystal structures (PDB ID: 1FBV, 3EB6, 3HCT, 1UR6, 2OXQ, and 2C2V) [47], [69], [70], [71], [84], [85]. Then, using respective amino acid sequences of E2s and RING-E3s from the 6 cocrystal structures, we identified the corresponding orthologs from *Drosophila melanogaster* by RBBHs. Sequence and structural alignments were conducted as described above; once an accurate alignment was determined, 3D complex models of orthologous RING-E3 and E2 pairs from *Drosophila melanogaster* were generated with Alignment Mode by computational simulation of coordinate templates. In addition, the solved RING-E3s (2EA6A) from the human was selected as a template for modeling the corresponding orthologs (Mura) from *Drosophila melanogaster*. Using the cocrystal structure of 3HCT data, we performed molecular docking of Mura-Ben. Ultimately, seven 3D complexes of RING-E3 and E2 pairs, including Cbl-UbcD10, Iap2-Eff, Traf6-Ben, Cont4- Eff, Chip-Eff, Chip-Ben, and Mura-Ben were established by replacement of the coordinates of their respective templates based on their structural similarity [86]. To assess the local quality of the predicted structure, we calculated final total free energy of all 3D models by the combination of Verify3D, ProQres (per-residue model accuracy estimation), Anolea (Anolea atomic mean force potential), and Gromos (empirical force field). Structural superimposition and RMSD calculation were conducted using the Discovery Studio Visualizer 2.0 program and ClusPro [87]. Finally, the 3D models of the complexes of these RING-E3 and E2 pairs from *Drosophila melanogaster* were submitted to the PMDB protein model database [88]. The interacting residues in the 3D complexes of RING-E3 and E2 pairs were deduced using DS Visualizer (http://accelrys.com/). The scripts show ligands and receptor binding site atoms within 4.0 Å between appropriate atoms in 3D structures for deducing the hydrophobic and polar interactions; ligand interacting residues (close approach) distance cut-off values are calculated within 3.5 Å by finding residues close to the current selection. The inferred interacting residues were further confirmed via manual examination using the Swiss-PDB viewer [89].

## Supporting Information

Figure S1 **Multiple sequence and structure alignments of the eight type RING domains from fruit fly.** According to the shared sequence conserved patterns of the corresponding site residues binding Zinc ions, a complete set of 139 RING domains from fruit fly were subdivided into eight types (**A**: C3HC4; **B**: C3H2C3 (RING-H2); **C**: C3HC3D; **D**: C4HC3 (RINGv); **E**: C3HGC3 (RING-G); **F**: C4C4 (RING-C2); **G**: C6H3C2D; **H**: U-box). The first lines of second structural arrangements of the types were respectively represented by the corresponding type orthologs with experimental structural data (C3HC4: 1FBVA; C3H2C3: 1X4JA; C3HC3D: 3HCTA; C4HC3: 2D8SA; C4C4: 1UR6B; C6H3C2D: 1U6GB; U-box: 2OXQC). The second structural arrangements of C3HGC3-type are the results of prediction by APSSP program due to lack the corresponding orthologous RING domain with experimental structural data. Green cylinders represent α-helices, green arrows represent β-strands, and grey lines represent loops. Except for U-box type without the full complement of Zn2+-binding ligands, the others are provided with the conserved *Cys/His* pattern. And the conserved metal ligand position and residues involved in coordinating Zinc ions were shadowed by grey. Equivalent residues of the conserved *Cys/His* in several members were replaced by non-*Cys/His* residues, which were shadowed by yellow for easy identification. Based on the previous structural evidence from 2BAY [6], the residues involved in stabilizing U-box were inferred, and shadowed by grey. Consensus amino acids were showed by pansy for easy identification. RING domains of C3HC4, C3H2C3, C4HC3, C4C4 and C3HGC3 types are stabilized by two Zinc ions coordinated by the conserved *Cys/His*, while C3HC3D and C6H3C2D/C types are stabilized by three Zinc ions. Because of far from RING domain, and more variability of metal ligand position and Zinc ions coordinating amino acid pairs, the third Zinc ion of C3HC3D-type was not represented. Residues (in place of the essential Zinc ions in the RING domains) contributing to the stabilization of U-box were shadowed by grey. *Gly*/*Pro* residues in short loop between β hairpin were indicated by bold letters for easy identification. The last two lines in different types showed consensus amino acid sequence (Con) for positions with a consensus level equal to or above 0.8 and conservation indices (Cons) for positions with a conservation index above 4. Consensus amino acid symbols are: conserved amino acids are in bold and uppercase letters; aliphatic (I, V, L): l; aromatic (Y, H, W, F): @; hydrophobic (W, F, Y, M, L, I, V, A, C, T, H): h; alcohol (S, T): o; polar residues (D, E, H, K, N, Q, R, S, T): p; tiny (A, G, C, S): t; small (A, G, C, S, V, N, D, T, P): s; bulky residues (E, F, I, K, L, M, Q, R, W, Y): b; positively charged (K, R, H): +; negatively charged (D, E): −; charged (D, E, K, R, H): c.
(PDF)

Figure S2 **Exonization of RING proteins of fruit fly without orthologs from the human.** (**A**) Exon duplication of CG31053 and CG12200 exon 1; (**B**) Exonization of long interspersed element (LINE) in CG5071 exon 1 and CG4325 exon 2; (**C**) Exonization of DNA element in CG17721 exon 1. The dots represent the same nucleotides as the consensus sequence. ID%: the percentage identity for pairwise sequence comparison.
(PDF)

Figure S3 **Multiple sequence and structure alignments of the solved RING domains.** **A:** C3HC4-type, C3H2C3-type (RING-H2), C3HC3D-type, C4HC3-type (RINGv) and C4C4-type; **B:** C6H3C2D-type/C6H2C4-type and U-box. Name of sequences were indicated by PDB IDs. Secondary structures of the sequences were colored according to experimentally-determined structural data (red letters: alpha-helix, blue letters: beta-strand). Four structurally conserved regions (SCRs) (1st: N-loop; 2nd: the first β-sheet region; 3rd: βα-region and 4th: C-loop) were indicated by green rectangles. Except from 1BORA and 2CSZA, all the others have a similar second structural arrangement of the ββα motif. The top lines of the alignments indicate the consensus secondary structure (SS); Conserved *Cys/His* residues binding zinc ions were shadowed by grey. Consensus amino acids were showed by pansy for easy identification. One exception is 3I2D with one occurrence of zinc ion at 3, 5, 7 and 8 positions, its corresponding site residues do not bind atom of zinc were shadowed by yellow (1, 2, 4 and 6 positions). *Gly*/*Pro* residues in short loop between β hairpin were indicated by bold letters for easy identification. The 4th metal-chelating residue position and zinc ion coordinating amino acids tend to be changeable in distinct type RING domains. The last 2 lines in different types showed consensus amino acid sequence (Consensus_aa) and conservation indices for positions with a conservation index above 4. Consensus amino acid symbols are: conserved amino acids are in bold and uppercase letters; aliphatic (I, V, L): l; aromatic (Y, H, W, F): @; hydrophobic (W, F, Y, M, L, I, V, A, C, T, H): h; alcohol (S, T): o; polar residues (D, E, H, K, N, Q, R, S, T): p; tiny (A, G, C, S): t; small (A, G, C, S, V, N, D, T, P): s; bulky residues (E, F, I, K, L, M, Q, R, W, Y): b; positively charged (K, R, H): +; negatively charged (D, E): −; charged (D, E, K, R, H): c.
(PDF)

Figure S4 **Sequence alignment of orthologous Diap2.** All the orthologous Iap2/Diap2 from arthropods possess a tandem repeat of 3 BIR domains and 1 RING domain. Apart from a tandem repeat of 3 BIR domains and 1 RING domain, all orthologous Iap2/Diap2 from vertebrates acquired an additional CARD domain. Iap2/Diap2 from arthropods lack the corresponding CARD domain, which were indicated by broken line rectangle. Hom, *Homo sapiens*; Pan, *Pan troglodytes*; Mac, *Macaca mulatta*; Pon, *Pongo abelii*; Mus, *Mus musculus*; Rat, *Rattus norvegicus*; Orn, *Ornithorhynchus anatinus*; Mon, *Monodelphis domestica*; Bos, *Bos taurus*; Equ, *Equus caballus*; Can, *Canis lupus familiaris*; Ory, *Oryctolagus cuniculus*; Dan, *Danio rerio*; Gal, *Gallus gallus*; Xen, *Xenopus (Silurana) tropicalis*; Dro, *Drosophila melanogaste*r; Drp, *Drosophila pseudoobscura*; Tri, *Tribolium castaneum*; Api, *Apis mellifera*; Nas, *Nasonia vitripennis*.
(PDF)

Figure S5 **Analysis of main-chain conformation of the solved RING domains.** (**A**) Superimposition of all the solved RING/non-U-box domains by sequence alignments using c-Alpha in each residue. Four regions (N-loop, the first β-sheet region, βα-region and C-loop) with reliable RMSD (average≤2.0 Å), lacking insertions and deletions were detected. (**B**) N-terminal loop superposed by 9 residues. (**C**) The first β-sheet region superposed by 7 residues. (**D**) βα-region superposed by 13 residues. (**E**) C-terminal loop superposed by 6 residues. (**F**) Superimposition of all the solved RING/U-box domains by sequence alingments. (**G**) Superimposition of 1FBVA (RING/non-U-box type) and 2OXQ (RING/U-box type) domains by sequence alingments. 2OXQ (RING/U-box type) are provided with structural extension at C-terminal (1FBVA: schematic style colored by yellow; 2OXQ: solid ribbon style colored by secondary structure elements). N: amino terminal; C: carboxyl terminal; β-sheet: antiparallel β-strands. The backbones of RING domains were superposed by C-Alpha atom in each residue.
(PDF)

Figure S6 **Cartoons of VDW surfaces created for all hydrophobic residues of RING domains.** Based on the van der Waals radius (VDW) of each atom in the molecule, VDW surface were created for all hydrophobic residues of RING domains, which were colored by Electrostatic potential (It calculates Gasteiger charges for the atoms that comprise the surface and maps the electrostatic potentials representing the charges to the surface). The spatial distribution of consensus and conservation of residues in RING domains were respectively indicated by blue (hydrophobic residues) and yellow letters (polar residues). 3D structures of RING domains were displayed by atom of ball and stick, which were colored by elements.
(PDF)

Figure S7 **Cartoons of 3D complexes of RING-E3 and E2 pairs.** **A:** Cbl-UbcD10; **B:** Iap2-Eff; **C:** Mura-Ben; **D:** Traf6-Eff; **E:** Cont4-Eff; **F:** Chip-Eff; **G:** Chip-Ben. **A-I to G-I:** Targets and templates were respectively represented by the purple ribbon models and the light blue schematic models. Structural similarity between them was shown by superimposition of their structures (N, amino terminal; C, carboxyl terminal). Cantact interfaces of RING-E3 and E2 complexes were illuminated by purple dot circles. I and II represent the first and the second Zn-binding sites. The 2 sites are respectively located on 2 sides of RING-E3 domain, by which form a characteristic “cross-brace” zinc-binding topology of RING domains. **A-II to G-II:** Close-up view of interaction residues in the intermolecular interfaces of their 3D complexes. The side chains of E2 and RING-E3 3D complexes involved in E2/E3 interactions were presented by solid ribbon. Resides that make significant directly contacts observed in the modeling complexes were presented by stick model, and were numbered by precursor peptides. The numbers for all resides in the figure correspond to those in the text and the tables. The interaction residues in the interface were respectively indicated by yellow (E2s) and white (RING-E3s) letters. The conserved hydrophobic contacts of intermolecular interfaces observed in the modeling complexes were highlighted by red dot circles. Hydrogen bonds of intermolecular interfaces of complexes formed by carbonyl-group oxygen and amino-group hydrogen were showed by white dot lines.
(PDF)

Figure S8 **Close-up view of interaction residues in the intermolecular interfaces of 3D complexes of RING-E3 and E2 pairs.** **A:** Cbl-UbcD10; **B:** Iap2-Eff; **C:** Mura-Ben; **D:** Traf6-Eff; **E:** Cont4-Eff; **F:** Chip-Eff; **G:** Chip-Ben. The side chains of 3D complexes of RING-E3 and E2 pairs involved in their interactions were presented by solid ribbon. Resides that make significant directly contacts observed in the modeling complexes were presented by stick model, and were numbered by precursor peptides. The numbers for all resides in the figure correspond to those in the text and the tables. The interaction residues in the intermolecular interface were respectively indicated by yellow (E2s) and white (RING-E3s) letters. The conserved hydrophobic contacts of intermolecular interfaces observed in the modeling complexes were highlighted by red dot circles. Hydrogen bonds of intermolecular interfaces formed by carbonyl-group oxygen and amino-group hydrogen were showed by white dot lines.
(PDF)

Table S1 Information related to RING proteins from *Drosophila melanogaster* and its corresponding putative orthologs from human.
(XLS)

Table S2 Identity and similarity percentages for all sequence pairs within different types.
(XLS)

Table S3 Domain architecture comparison of orthologous RING proteins from *Drosophila melanogaster* and the human.
(XLS)

Table S4 RMSD values for pairwise C-Alpha atom superposed by sequence alignments.
(XLS)

Table S5 RMSD values for the 4 SCRs for pairwise superposed by sequence alignments using c-Alpha in each residue.
(XLS)

Table S6 Hydrophobic residues identified close to the central hydrophobic residues within 4 Å.
(DOC)

Table S7 Information relevant to interolog interactions of RING-E3 and E2 pairs from *Drosophila melanogaster* and the human.
(XLS)

Table S8 Summary of interolog interactions of E2s and RING-E3s from fruit fly.
(PDF)
