# Supplementary material for: Comprehensively Surveying Structure and Function of RING Domains from Drosophila melanogaster
Source: PLoS One. 2011 Sep 2;6(9):e23863. doi: 10.1371/journal.pone.0023863 (PMC3166285; doi:10.1371/journal.pone.0023863)
Supplement: Figure S1 — Multiple sequence and structure alignments of the eight type RING domains from fruit fly. According to the shared sequence conserved patterns of the corresponding site residues binding Zinc ions, a complete set of 139 RING domains from fruit fly were subdivided into eight types (A: C3HC4; B: C3H2C3 (RING-H2); C: C3HC3D; D: C4HC3 (RINGv); E: C3HGC3 (RING-G); F: C4C4 (RING-C2); G: C6H3C2D; H: U-box). The first lines of second structural arrangements of the types were respectively represented by the corresponding type orthologs with experimental structural data (C3HC4: 1FBVA; C3H2C3: 1X4JA; C3HC3D: 3HCTA; C4HC3: 2D8SA; C4C4: 1UR6B; C6H3C2D: 1U6GB; U-box: 2OXQC). The second structural arrangements of C3HGC3-type are the results of prediction by APSSP program due to lack the corresponding orthologous RING domain with experimental structural data. Green cylinders represent α-helices, green arrows represent β-strands, and grey lines represent loops. Except for U-box type without the full complement of Zn2+-binding ligands, the others are provided with the conserved Cys/His pattern. And the conserved metal ligand position and residues involved in coordinating Zinc ions were shadowed by grey. Equivalent residues of the conserved Cys/His in several members were replaced by non-Cys/His residues, which were shadowed by yellow for easy identification. Based on the previous structural evidence from 2BAY [6], the residues involved in stabilizing U-box were inferred, and shadowed by grey. Consensus amino acids were showed by pansy for easy identification. RING domains of C3HC4, C3H2C3, C4HC3, C4C4 and C3HGC3 types are stabilized by two Zinc ions coordinated by the conserved Cys/His, while C3HC3D and C6H3C2D/C types are stabilized by three Zinc ions. Because of far from RING domain, and more variability of metal ligand position and Zinc ions coordinating amino acid pairs, the third Zinc ion of C3HC3D-type was not represented. Residues (in place of the essential Zinc ions in the RING [file pone.0023863.s001.pdf]

Ying et al. Figure S1

A C3HC4-type

N-loop — Helix — C-loop

Zn^{2+} metal ligand pairs: 1, 2 / 3, 4 / 5, 6 / 7, 8 (Metal ligand)

```
SS
Motif     CXXC....................CXHX2-3CXXC......................................CXXC....
1FBVA[1] LCKICAENDK---------DVKIEPCGH-LMCTSCLTSWQESE---------------------------GQGCPFCRCEI
CG7037   LCKICAENDK---------DIRIEPCGH-LLCTPCLTSWQVDSE--------------------------GQGCPFCRAEI
CG10981  KCPICMDSVSKR-------EPVSTKCGH-VFCRECIETAIRATH----------------------------KCPICNKKL
CG5595   MCPICLDMLKK--------TMTTKECLH-RFCSDCIVTALRSGN---------------------------KECPTCRKKL
CG5841   MCPVCFDRI----------KNMVFLCGH-GTCQMCGDQIE--------------------------------GCPICRKTV
CG12284  LCKICYGAEY---------NTAFLPCGHVVACAKCASSVT--------------------------------KCPLCRKPF
CG1134   LCVVCSTNPK---------EIILLPCGHVCLCEDCAQKISV-------------------------------TCPVCRGSI
CG15104  NCAICLSRCRR--------KCFTDSCMH-QFCFKCLCEWSKIKP----------------------------ECPLCKQPF
CG11988  ECTICYENPI---------DSVLYMCGHMCMCYDCAIEQWRGVG--------------------------GGQCPLCRAVI
CG10542  TCPSCKVKRK---------DAVLSKCFH-VFCYDCLRTRYETRQ---------------------------RKCPKCNCAF
CG4909   ECSVCLERLDT--------TSKVLPCQH-TFCRKCLQDIVASQ------HKL--------------------RCPECRILV
CG3886   ICHLCQGYLIN--------ATTIVECLH-SFCHSCLINHLRKER----------------------------FCPRCEMVI
CG3905   TCRLCRGYMID--------PTTVDYCYH-TYCRSCILKHLLRAV----------------------------YCPECKASG
CG3231   ICGICRDIFVD--------AVMIPCCGS-SFCDDCVRTSLLESE--------------------------DSECPDCKEKN
CG8293   LCKVCLDEEV---------GVVFLPCGHLATCNQCAPSVA--------------------------------NCPMCRADI
CG6752   LCPICCAKPI---------TAVFTPCKH-QSCSDCIMQHMMNSK----------------------------VCFYCKTTI
CG31721  RCPTCKQLYA---------NPVLLPCFH-ALCLGCALDIQTPYS---PGSALPGAVNGAGAA[101]AAYCLTCPLCRKLV
CG12218  LCKLCSLRLV---------MPRILSCLH-VFCEDCLQAVVSKDTMSASSPNSCSSSSFDGSEHQNRLSAVFIECPVCKQET
CG9941   ECVICMSETR---------DTLILPCRHLCLCNSCADSLRYQA----------------------------NNCPICRAPF
CG17019  LCKICMDAPI---------ECVFLECGHMATCTSCGKVLN--------------------------------ECPICRQYI
CG12489  QCKICMDRAI---------NTVFNPCCHVIACAQCAARCS--------------------------------NCPNCRVKI
CG15105  TCCVCLDRYR---------IPKLLPCQH-SFCMEPCMEGLVDYV----RRQV--------------------KCPECRAEH
CG5206   KCVYCAQLLGSND------RPKLLECLH-VACAQCVSTKFSELD---RSLPPLI------------------HCPVCDNAS
CG8419   KCGICLDVYT---------DPRTLHCLH-SFCLQCLVSENFKD[59]KSDGKRSTSSIGTRFTGSFASEVSTRCIRCHTCN
CG8786   ECPICLQTCI---------HPARLPCGH-IFCFLCVKGVAYKNR----------------------------RCAMCRREI
CG7614   ACPRCKTTKY----RNPSLKLMVNVCGH-TLCESCVDLLFLKGS---------------------------GACPECMVPL
CG9949   ECPVCFDYVLP--------PILQCSSGH-LVCVSCRSKLT--------------------------------CCPTCRGPL
CG13030  ECPVCFGYIMP--------PIMQCPRGH-LICSTCRSKLT--------------------------------ICPVCRVFM
CG2681   RCPGCAGAMKA--------PILLCKSGH-SVCEQCTRILL--------------------------------MCPLCKEPF
CG7864   QCILCLEPRS---------DSSLTPCGH-IFCWSCLLEWLEERD----------------------------ECPLCRESL
CG3639   ECPVCLLSIQT--------PTACSVSGY-VFCWKCIVSHMKEHG----------------------------TCPVTHYPI
CG7376   PCPICQTQDDV--------RYVMMVCGH-FVCQHCLDSMRRKNG---RAGVT--------------------KCPLCRQDS
CG5334   MCGICLETVVKK-RGRECRFGILPKCKH-IFCLTCIRRWRQAEY---IEDNVKR------------------GCPECRVFS
CG7184   TCGICFDTIMEK-AGREKRFGILPNCNH-IFCLECIRTWRQAKQ---FENKITR------------------ACPECRVCS
CG5347   MCGICFDTVVEK-KGRERRFGILSKCKH-IFCLTCIRTWRQAHQ---FEATVTR------------------GCPECRVFS
CG12477  KCGICFETIMEK-EGGDKRFGILPSCNH-VFCFQCICTWRHATQ---YAYQVTR------------------ACPECRVWS
CG7081   TCTFCGERPTL---------PHHMGCGH-IYCYYCLNANVLTDA--------------------------SFCCPNCGSAC
CG5659   ECEICFSQLPP-------DSMAGLECGH-RFCMPCWHEYLSTKI---VAEGLGQ----------------TISCAAHGCDI
CG12362  LCGICFCSCD---------ELIGLGCGH-NFCAACWKQYLANKT---CSEGLAN----------------TIKCPAANCEI
CG5709   KCHICI--------------EKNGGCNH-MQCFNCKHDF----------------------------------CWMCLGDW
CG11360  ECFVCNENTVT---------TALVPCGHNMFCMECANHICLSMD---------------------------AVCPVCNSIV
CG17492  ECIVCNEILPM---------VRFEPCQHQIACEECGIRMK--------------------------------KCLRCAVAI
CG11321  ECELCMNSYPMNQ------MVSMLKCLH-KCCKQCAKSYFTVQI---TDRSIND-----------------CSCPFCKLPE
CG8651   MCLFCRKSGE----GLSGEEARLLYCGHDCWVHTNCAM[20]ARG-----RMI-------------------KCTVCGNRG
CG4030   NCPTCNAYFT-----VMVRKIHCRHCGH-IYCDKCLTKTVPSGP---RKRVAR-------------------VCDICHTLL
CG4620   KCMKCEENNR---------TVTLEPCNHLSICNTCAESVT--------------------------------ECPYCQVPV
CG12099  QCPICLYPPV---------AAKLTRCGH-AYCWPCLLHYLSLSD----KTWR--------------------KCPICYDAI
CG11414  ACVVCFKNVE---------IFSIGDCDH-PVCYECSTRMRVLCQ--------------------------QNECPICRHVL
CG2617   RCVVCMAQSR---------NVVVMPCRHLCLCKECSLQLVLLLE---------------------------DRCPVCRHNI
CG13605  LCPICHDAFN---------TPTVLECGH-IFCDECVQTWFKREQ----------------------------TCPMCRAKV
CG8974   ECNICLDTAK---------DAVVSMCGH-LFCWPCLHQWLLTRP-----NRK--------------------LCPVCKAAV
CG32581  ECNICLDTAK---------DAVVSMCGH-LFCWPCLHQWLLTRP-----NRK--------------------LCPVCKAAV
CG32847  ECNICLDTAQ---------NAVVSMCGH-LFCWPCLYQWILTKP-----DHT--------------------VCPVCKSGV
CG34308  TCLVCMQTAE---------SPRVSFCGH-HFCSQCIYNWIRSQK-----YQA--------------------KCPYCQSLI
CG32369  MCPLCSDILR---------CPVTTNCGH-TFCRQCCETITQ--------------------------------CNICQVRF
CG4195   TCKICGGYFI--------DATTVTECLH-TFCKSCLVKHLEEKK----------------------------TCPTCDNII
CG4973   KCHICRQSFV---------NPVVTKCKH-YFCEKCALAQYKKSQ----------------------------RCIICSQQT
CG4813   KCLICIDDYK--------NPAISVSCWH-VHCEQCWLQTLGARK----------------------------LCPQCNSIT
CG10263  CCDQCDKPIL--------VYGRMIPCKH-VFCLKCARAEPIK------------------------------SCPRCTDKV
CG12200  HCNKCFRHRK----TDPAVPFHLTQCRH-VICGPCLGQSSLEK-----------------------------NCPLCGQVL
CG17048  MCAFCLDRIQ---------NPEKLHCNH-AFCKSCLALYREARN----WVAK--------------------RCPICRSSL
CG34375  ECPVCLEVIKP--------PGWQCCNGH-VLCNNCRSRSV--------------------------------KCPVCRVPL
```

```
CG15150 NCPVCLEDVREK-------LPVSTNCGH-VFCKACIKRAVDTGR---------------------------VCPLCGVDE
CG34289 PCIFCLDEQR---------YPHHISCGH-SFCSACLKKYLELGR---------------------------SRCPLCRRDF
CG31053 HCNKCFRRRN----VEPTLIFHMTQCQH-VLCASCLSESSTDKD---------------------------KCPLCKRDL
CG8141  VCNECNQYVR---------GGVITICGH-LFCWTCLWPKLSGT------AQP-------------------RCPCCQRHL
CG14306 KCSKCFTYLYNTNSEEDNHRPFLLGCGH-TLCESCLWNDRLDP----------------------------KCAVCHSPA
CG5071  SCGLCHRPYD----LATGLLPQELACRH-SFCKKCVQRNTDHNS-----SEC---------------------ICNLCGYRT
CG8910  ECVICINARA---------TMQTSPCGHRVVCRRCFVKTIQSAV---AQRLLPL------------------RCVICRARV
Con      .C.hC...................C.H..hC.pCh.p...................................Cs.Cp..h
Cons     9  9                     7 8   9   9                                     9  7
Section
```

B C3H2C3-type

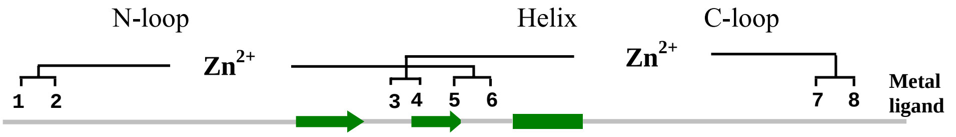


```
SS
Motif    CXXC........................CXHXXHXXC.........................CXXC....
1X4J_A  LCVVCMCDFESRQ-----------LLRV-LPCNHEFHAKCVDKWLKA---------------NRTCPICRADS
CG9381  SCVVCMCDFELR------------QLLRVLPCSHEFHAKCVDKWLRSNR---------------TCPICRGNA
CG5140  NCVICAELFGQAD-----------E-VFATVCGHMFHHNCLNQWLDR---------------SKTCPQCRNKC
CG1909  NCGACGELLGLR-----------PENLEALPCAHILHARCAYEILRRREK----------NAPRSCPACNKLI
CG5555  TCPVCLERMDESV-----------DGVLTILCNHAFHASCLMKWGD-----------------STCPVCRHVQ
CG6923  KCAICLNLFEIE------------NEVRRLPCMHLFHTDCVDQWLVT---------------NKHCPICRVDI
CG7694  ECSVCKEPAEEG------------QKYRILPCKHEFHEECILLWLKKT---------------NSCPLCRYEL
CG11982 QCSICWDDFKID------------ETVRKLPCSHLYHENCIVPWLNLH---------------STCPICRKSL
CG2304  VCAICYQEMY---------------SAKITRCRHFFHGVCLRKWLYV---------------QDRCPLCHEIM
CG32850 ECVICMAEFCVN------------EAVRYLPCMHIYHVNCIDDWLLR---------------SLTCPSCLEPV
CG5382  NCTLCGTTLEQGD-------------CVRLVCYHVFHWDCLNARQAALPANTA-------PRGHQCPACSVEI
CG3093  TCEICEMMLLVKP-------------FFIFICGHKFHSDCLEKHVVPLLTKEQC[44]EDILAADCLFCGLLI
CG15814 VCAVCGNQLLVSEKEE-----GVIENTYKLSCNHVFHEFCIRGWCIVGK-------------KQTCPYCKEKV
CG13344 QCVVCLYGFADG------------DEFTRTECFHYLHSYCLARHLNALRRNYQEEFDK[12]QALCPVCREHI
CG3647  ECLVCVEAIKSHQ-----------PTWSCRNCYHMLHLKCTITWASSSKS----------EVGWRCPACQNVL
CG10277 TCVICLEDFIED------------DKLRVLPCSHPYHTHCIDPWLTENR--------------RVCPICKRKV
CG16947 HCPVCLGDIHTS-----------RIPCHIPDCGHLLHKMCFDQLLASG--------------HYTCPTCQTSL
CG2679  CCAICIEAYKPT------------DTIRILPCKHEFHKNCIDPWLIEH---------------RTCPMCKLDV
CG42593 DCIICNCTTPSTESNPI[66]AYDNGVHVQSCGHHVHLSCLEAYLKTLYTTQRQPVQD--RGEFYCPVCRQLS
CG32350 KCDICREMLSMQ--------------SIYFLCQHSFHEECLNYKSTKRQE------------KFLCIICKTRN
CG18028 LCSLCHRPVLMAG--------THLYCIIRLECGHVYHKPCIQGELLK-----------------NCNECNLWK
CG10144 KCSMCRQRLYDHS-----------QVLIFGGCGHGIHEQCMEESETQ----------------FEECPRCFTAI
CG10916 LCAICNEFFRAND-----------IIFSTSRCGHVFHKDCLTRWLNR---------------SRTCPQCRDPC
CG4325  ICTICSERFRTS------------DNIQAGSCGHAFHEDCLDHWRKQS---------------RTCPICRSQD
CG17721 LCTICLDPLSVY-------------TMVYLRCSHALHEKCLHQYQANG--------------GRHCPVCRMGI
CG17260 RCPICDSTVLPEYPEDIETDDNADFFIERVYCGHLFHQGCLKKYLSEPPFP---------KGGKLCPAARRHP
CG1937  ICIICREDMVNH---------------SKKLPCGHIFHTTCLRSWFQRQ---------------QTCPTCRLNI
CG3929  PCPMCMEELVH---------SAQNPAISLSRCQHLMHLQCLNGMIIAQQ---NEMNKNL---FIECPVCGIVY
CG9086  KCILCFENCAISGGRQL [20]SQSALHISCCGHVMHYSCWLEYFTNEEF[11]ALAQAANVEFQCPYCRTLS
CG32592 MCMICFVEALSCAP-----------SIHLEECGHVFHYHCCKAVLEKRWSGPRITFG-----FSLCPICKADI
Con      .C.lC...h.................h..h.C.H.hH..Ch.ph...................pCPhCp...
Cons     9 59                         9 9  9  9                          97 9
```

C C3HC3D-type (3Zn+)

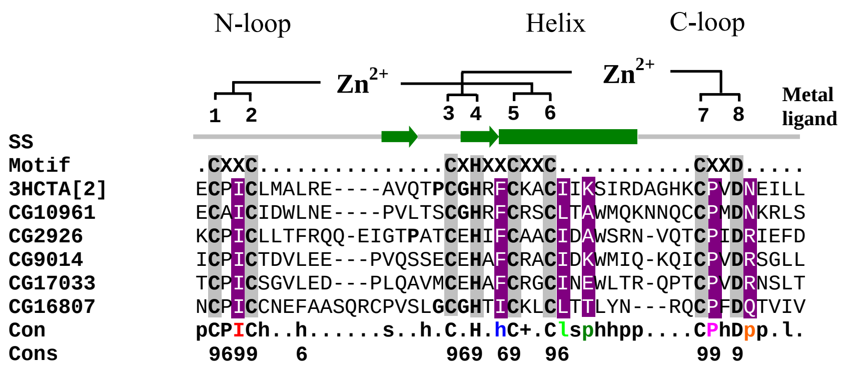

D C4HC3-type

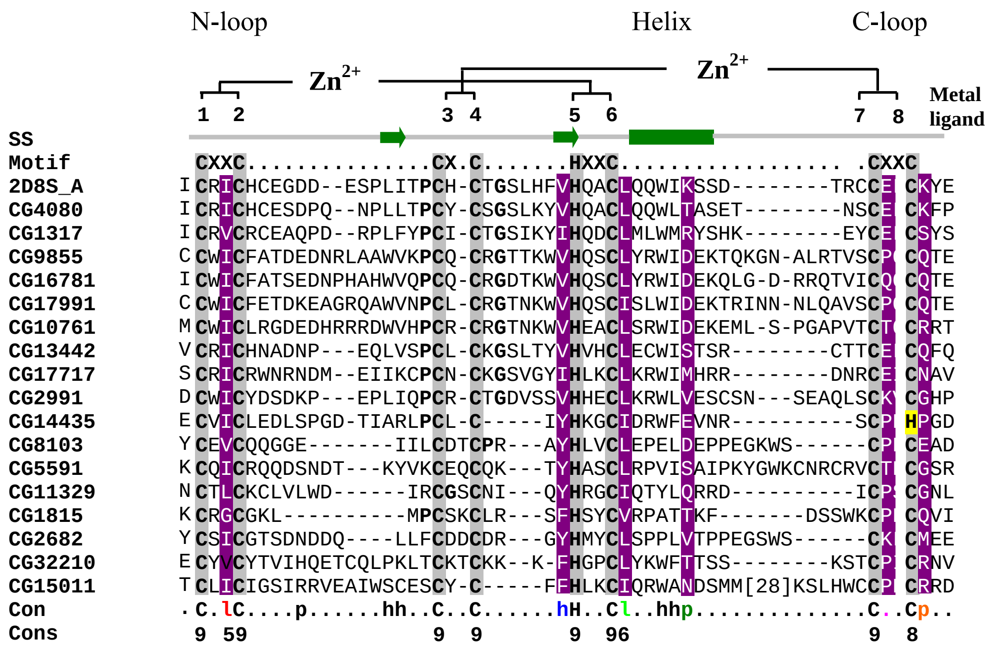

E C3HGC3-type

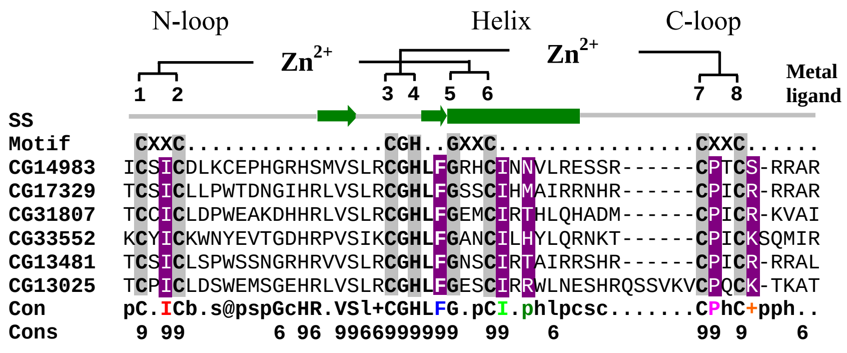

F C4C4-type

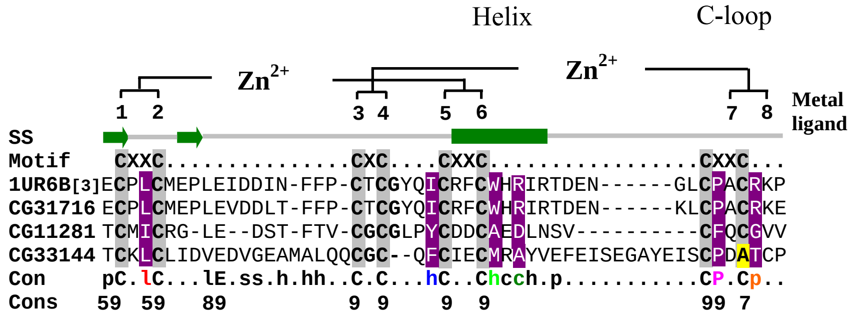

G C6H3C2D-type (3Zn+)

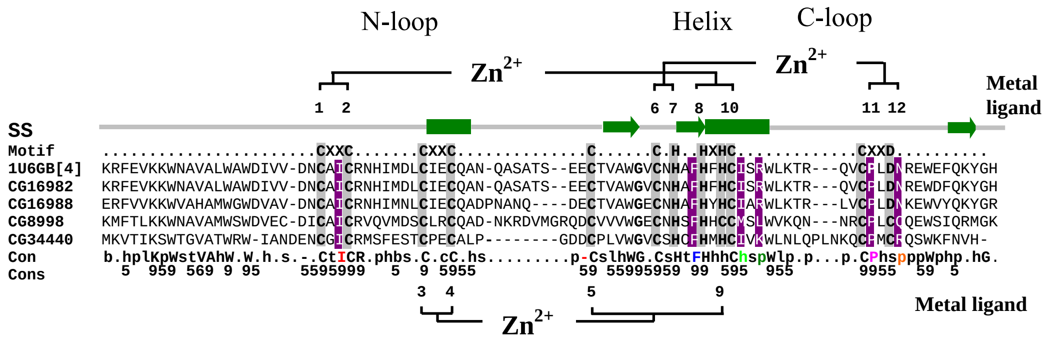

H U-box

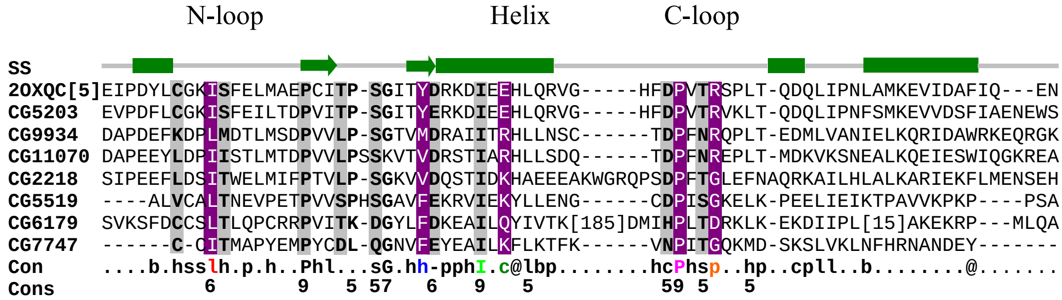

Figure S1. Multiple sequence and structure alignments of the eight type RING domains from fruit fly. According to the shared sequence conserved patterns of the corresponding site residues binding Zinc ions, a complete set of 139 RING domains from fruit fly were subdivided into eight types (**A**: C3HC4; **B**: C3H2C3 (RING-H2); **C**: C3HC3D; **D**: C4HC3 (RINGv); **E**: C3HGC3 (RING-G); **F**: C4C4 (RING-C2); **G**: C6H3C2D; **H**: U-box). The first lines of second structural arrangements of the types were respectively represented by the corresponding type orthologs with experimental structural data (C3HC4: 1FBVA; C3H2C3: 1X4JA; C3HC3D: 3HCTA; C4HC3: 2D8SA; C4C4: 1UR6B; C6H3C2D: 1U6GB; U-box: 2OXQC). The second structural arrangements of C3HGC3-type are the results of prediction by APSSP program due to lack the corresponding orthologous RING domain with

experimental structural data. Green cylinders represent α-helices, green arrows represent β-strands, and grey lines represent loops. Except for U-box type without the full complement of Zn2+-binding ligands, the others are provided with the conserved *Cys/His* pattern. And the conserved metal ligand position and residues involved in coordinating Zinc ions were shadowed by grey. Equivalent residues of the conserved *Cys/His* in several members were replaced by non-*Cys/His* residues, which were shadowed by yellow for easy identification. Based on the previous structural evidence from 2BAY [6], the residues involved in stabilizing U-box were inferred, and shadowed by grey. Consensus amino acids were showed by pansy for easy identification. RING domains of C3HC4, C3H2C3, C4HC3, C4C4 and C3HGC3 types are stabilized by two Zinc ions coordinated by the conserved *Cys/His*, while C3HC3D and C6H3C2D/C types are stabilized by three Zinc ions. Because of far from RING domain, and more variability of metal ligand position and Zinc ions coordinating amino acid pairs, the third Zinc ion of C3HC3D-type was not represented. Residues (in place of the essential Zinc ions in the RING domains) contributing to the stabilization of U-box were shadowed by grey. *Gly*/*Pro* residues in short loop between β hairpin were indicated by bold letters for easy identification. The last two lines in different types showed consensus amino acid sequence (Con) for positions with a consensus level equal to or above 0.8 and conservation indices (Cons) for positions with a conservation index above 4. Consensus amino acid symbols are: conserved amino acids are in bold and uppercase letters; aliphatic (I, V, L): l; aromatic (Y, H, W, F): @; hydrophobic (W, F, Y, M, L, I, V, A, C, T, H): h; alcohol (S, T): o; polar residues (D, E, H, K, N, Q, R, S, T): p; tiny (A, G, C, S): t; small (A, G, C, S, V, N, D, T, P): s; bulky residues (E, F, I, K, L, M, Q, R, W, Y): b; positively charged (K, R, H): +; negatively charged (D, E): -; charged (D, E, K, R, H): c.

Reference for Figure S1:

1. Zheng N, Wang P, Jeffrey PD, Pavletich NP (2000) Structure of a c-Cbl-UbcH7 complex: RING domain function in ubiquitin-protein ligases. Cell 102: 533-539.
2. Yin Q, Lin SC, Lamothe B, Lu M, Lo YC, et al. (2009) E2 interaction and dimerization in the crystal structure of TRAF6. Nat Struct Mol Biol 16: 658-666.
3. Dominguez C, Bonvin AM, Winkler GS, van Schaik FM, Timmers HT, et al. (2004) Structural model of the UbcH5B/CNOT4 complex revealed by combining NMR, mutagenesis, and docking approaches. Structure 12: 633-644.
4. Goldenberg SJ, Cascio TC, Shumway SD, Garbutt KC, Liu J, et al. (2004) Structure of the Cand1-Cul1-Roc1 complex reveals regulatory mechanisms for the assembly of the multisubunit cullin-dependent ubiquitin ligases. Cell 119: 517-528.
5. Xu Z, Kohli E, Devlin KI, Bold M, Nix JC, et al. (2008) Interactions between the quality control ubiquitin ligase CHIP and ubiquitin conjugating enzymes. BMC Struct Biol 8: 26.
