# Supplementary material for: Comprehensively Surveying Structure and Function of RING Domains from Drosophila melanogaster
Source: PLoS One. 2011 Sep 2;6(9):e23863. doi: 10.1371/journal.pone.0023863 (PMC3166285; doi:10.1371/journal.pone.0023863)
Supplement: Figure S2 — Exonization of RING proteins of fruit fly without orthologs from the human. (A) Exon duplication of CG31053 and CG12200 exon 1; (B) Exonization of long interspersed element (LINE) in CG5071 exon 1 and CG4325 exon 2; (C) Exonization of DNA element in CG17721 exon 1. The dots represent the same nucleotides as the consensus sequence. ID%: the percentage identity for pairwise sequence comparison. (PDF) [file pone.0023863.s002.pdf]

A

```
CG31053    CGTGGCCCAATACTTTGAGGATCCGCTTCGATTCCAGCAGCTCTACCGCAAGATCAGCAAGTTCCAGGCCG
CG12200    .......A.C.....C.C......C..G............A.............TC..........A..G.

CG31053    ACCAGCGGGCATCGGACAACCTGGGATTCTATCGCCAGATGCAGGAGCACGAGAAGAACGAGAGCCGCTT
CG12200    .......A.................T.....C..T...T.....C...TG...C.A...A..C...AGC.

                                                  Location        ID%
CG31053    AAAGGGCTTCTGCAAGATGGAGGCGCAGTTCAACCAG exon1: 207-384   100
CG12200    GG.......T..................C.G...... exon1: 229-406   82.6
```

B

```
                                                                                Location         ID%
CG5071   CGAGAATTGGTGCGC--CAGTTGCAACAGCAGCCGCAGCAAGTGCAACAGGAACAGCAGCAGCAGCAGCAA exon1: 1374-1445  100
Line     ..GC.....A.AG..AA...CA...C..A..A.A.......CA......AC....................  TART: 6943-7016   77.0


                                                                           Location       ID%
CG4325   CCAAGGTCACAGCAGCAGCAGCAGCAGCTGCAGCAGCAACAACAACAACAGTAACGACAACAGCAGCA exon2: 124-191   100
Line     ....A...-.....A..A..A.....T.GA..............T..C...C.G.AG.....A.....  TART: 6160-6226  77.9
```

C

```
                                                         Location        ID%
CG17721  ATATATATTCTTGATCATGATCAATAGGCCAGTTGATGTGGCCA exon1: 88-131     100
DNAREP1  .................G.........C.G...C...C...... DNArep1:106-149   88.6
```

Figure S2. Exonization of RING proteins of fruit fly without orthologs from the human. **(A)** Exon duplication of CG31053 and CG12200 exon 1; **(B)** Exonization of long interspersed element (LINE) in CG5071 exon 1 and CG4325 exon 2; **(C)** Exonization of DNA element in CG17721 exon 1. The dots represent the same nucleotides as the consensus sequence. ID%: the percentage identity for pairwise sequence comparison.
