# Supplementary material for: Comprehensively Surveying Structure and Function of RING Domains from Drosophila melanogaster
Source: PLoS One. 2011 Sep 2;6(9):e23863. doi: 10.1371/journal.pone.0023863 (PMC3166285; doi:10.1371/journal.pone.0023863)
Supplement: Figure S3 — Multiple sequence and structure alignments of the solved RING domains. A: C3HC4-type, C3H2C3-type (RING-H2), C3HC3D-type, C4HC3-type (RINGv) and C4C4-type; B: C6H3C2D-type/C6H2C4-type and U-box. Name of sequences were indicated by PDB IDs. Secondary structures of the sequences were colored according to experimentally-determined structural data (red letters: alpha-helix, blue letters: beta-strand). Four structurally conserved regions (SCRs) (1st: N-loop; 2nd: the first β-sheet region; 3rd: βα-region and 4th: C-loop) were indicated by green rectangles. Except from 1BORA and 2CSZA, all the others have a similar second structural arrangement of the ββα motif. The top lines of the alignments indicate the consensus secondary structure (SS); Conserved Cys/His residues binding zinc ions were shadowed by grey. Consensus amino acids were showed by pansy for easy identification. One exception is 3I2D with one occurrence of zinc ion at 3, 5, 7 and 8 positions, its corresponding site residues do not bind atom of zinc were shadowed by yellow (1, 2, 4 and 6 positions). Gly/Pro residues in short loop between β hairpin were indicated by bold letters for easy identification. The 4th metal-chelating residue position and zinc ion coordinating amino acids tend to be changeable in distinct type RING domains. The last 2 lines in different types showed consensus amino acid sequence (Consensus_aa) and conservation indices for positions with a conservation index above 4. Consensus amino acid symbols are: conserved amino acids are in bold and uppercase letters; aliphatic (I, V, L): l; aromatic (Y, H, W, F): @; hydrophobic (W, F, Y, M, L, I, V, A, C, T, H): h; alcohol (S, T): o; polar residues (D, E, H, K, N, Q, R, S, T): p; tiny (A, G, C, S): t; small (A, G, C, S, V, N, D, T, P): s; bulky residues (E, F, I, K, L, M, Q, R, W, Y): b; positively charged (K, R, H): +; negatively charged (D, E): −; charged (D, E, K, R, H): c. (PDF) [file pone.0023863.s003.pdf]

Ying et al. Figure S3

**A: C3HC4, C3H2C3, C3HC3D, C4HC3 and C4C4 types**

```
                     1st                               2nd              3rd                      4th
SS
1FBVA   1  LCKICAENDKD------------------VKIEPCG----H-LMCTSCLTSWQESEGQ-------GCPF--CRCE 43
3HCTA   1  ECPICLMALRE------------------AVQTPCG----H-RFCKACIIKSIRDAGH-------KCPV--DNEI 43
1RMDA   1  SCQICEHILAD------------------PVETSCK----H-LFCRICILRCLKVMG-------SYCPS--CRYP 43
2CKLB   1  MCPICLDMLKN-----------------TMTTKECL----H-RFCADCIITALRSGN-------KECPT--CRKK 44
2CKLA   1  MCVLCGGYFID-----------------ATTIIECL----H-SFCKTCIVRYLETSK--------YCPI--CDVQ 43
2ECJA   1  SCSVCLEYLKE------------------PVIIECG----H-NFCKACITRWWEDLER-----DFPCPV--CRKT 45
2ECWA   1  TCPICLELLKE------------------PVSADCN----H-SFCRACITLNYESNRNT--DGKGNCPV--CRVP 48
1JM7A   1  ECPICLELIKE------------------PVSTKCD----H-IFCKFCMLKLLNQKKG-----PSQCPL--CKND 45
2ECNA   1  ECCICMDGR-------------------ADLILPCA----H-SFCQKCIDKWSDRHR--------NCPI--CRLQ 41
1JM7B   1  RCSRCTNILRE-----------------PVCLGGCE----H-IFCSNCVSDCIGT----------GCPV--CYTP 41
2CSYA   1  RCFICRQAFQN------------------PVVTKCR----H-YFCESCALEHFRATP--------RCYI--CDQP 42
1CHCA   1  RCPICLEDPSN-----------------YSMALPCL----H-AFCYVCITRWIRQNP--------TCPL--CKVP 43
1Z6UA   1  MCVCCQELVYQ------------------PVTTECF----H-NVCKDCLQRSFKAQV-------FSCPA--CRHD 43
2CT2A   1  ECPICMESFTEEQ--------------LRPKLLHCG----H-TICRQCLEKLLASSIN-----GVRCPF--CSKI 49
3FL2A   1  QCICCQELVFR------------------PITTVCQ----H-NVCKDCLDRSFRAQV-------FSCPA--CRYD 43
2EGPA   1  TCPICLELLTE------------------PLSLDCG----H-SLCRACITVSNKEAVTSM-GGKSSCPV--CGIS 49
2ECVA   1  TCPICLELLTQ------------------PLSLDCG----H-SFCQACLTANHKKSMLD--KGESSCPV--CRIS 48
2EA6A   1  SCPICMDGYSEIVQ-----------NGRLIVSTECG----H-VFCSQCLRDSLKNAN--------TCPT--CRKK 49
1BORA   1  RCQQCQAEAKC------------------PKLLPCL----H-TLCSGCLEASGM-----------QCPI--CQAP 39
2YSLA   1  ICPICLDILQK------------------PVTIDCG----H-NFCLKCITQIGETSCG-----FFKCPL--CKTS 45
2D8TA   1  ECAICLQTCVH------------------PVSLPCK----H-VFCYLCVKGASWLGK--------RCAL--CRQE 42
3KNVA   1  LCSACRNVLRR------------------PFQAQCG----H-RYCSFCLASILSSGP-------QNCAA--CVHE 43
```

C3HC4-type

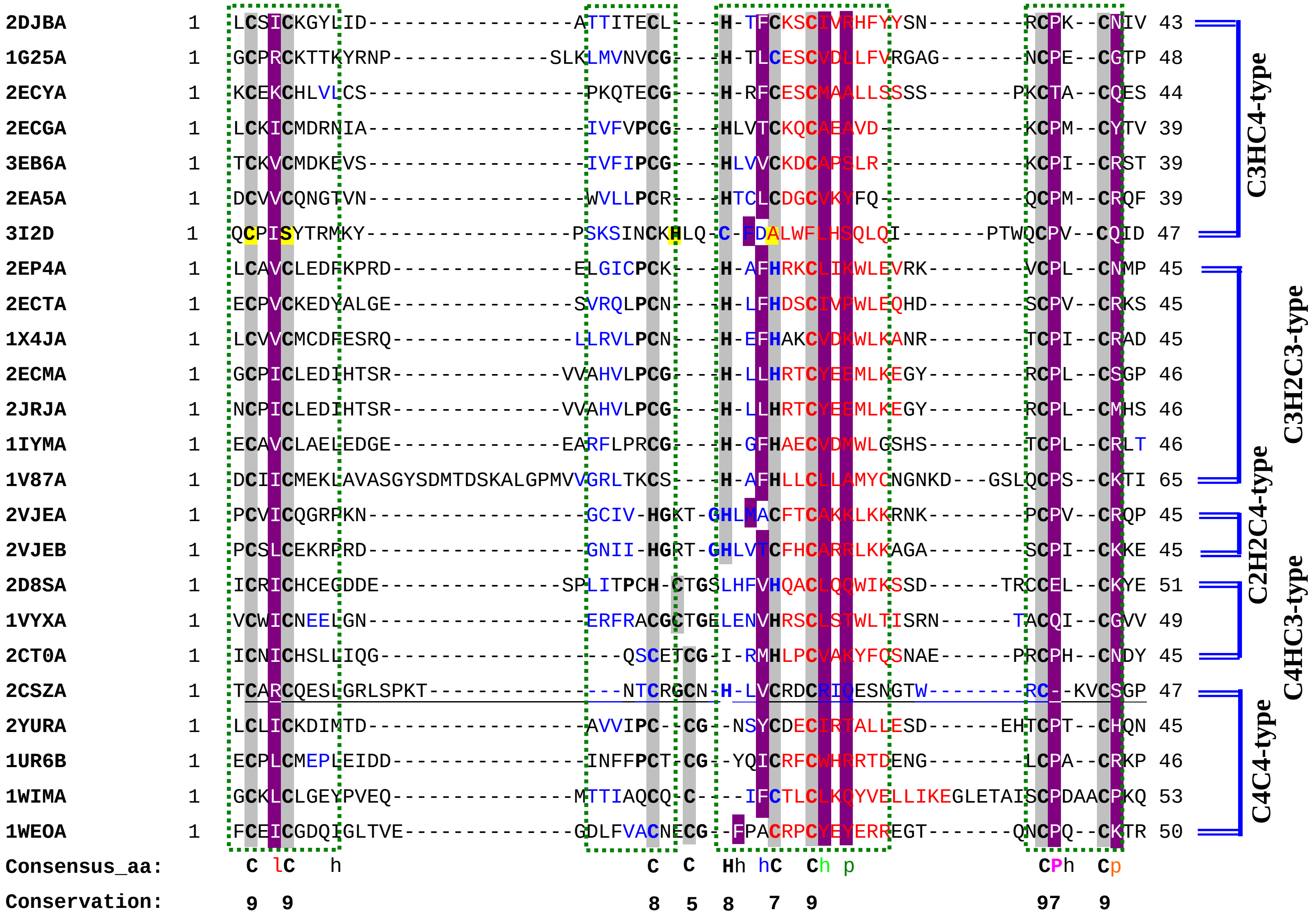
2DJBA 1 LCSICKGYLID-----------------ATTITECL----H-TFCKSCIVRHFYYSN--------RCPK--CNIV 43
1G25A 1 GCPRCKTTKYRNP-------------SLKLMVNVCG----H-TLCESCVDLLFVRGAG-------NCPE--CGTP 48
2ECYA 1 KCEKCHLVLCS------------------PKQTECG----H-RFCESCMAALLSSSS-------PKCTA--CQES 44
2ECGA 1 LCKICMDRNIA------------------IVFVPCG----HLVTCKQCAEAVD------------KCPM--CYTV 39
3EB6A 1 TCKVCMDKEVS------------------IVFIPCG----HLVVCKDCAPSLR------------KCPI--CRST 39
2EA5A 1 DCVVCQNGTVN------------------WVLLPCR----HTCLCDGCVKYFQ------------QCPM--CRQF 39
3I2D 1 QCPISYTRMKY-----------------PSKSINCKHLQ-C-FDALWFLHSQLQI-------PTWQCPV--CQID 47
2EP4A 1 LCAVCLEDFKPRD---------------ELGICPCK----H-AFHRKCLIKWLEVRK--------VCPL--CNMP 45
2ECTA 1 ECPVCKEDYALGE---------------SVRQLPCN----H-LFHDSCIVPWLEQHD--------SCPV--CRKS 45
1X4JA 1 LCVVCMCDFESRQ---------------LLRVLPCN----H-EFHAKCVDKWLKANR--------TCPI--CRAD 45
2ECMA 1 GCPICLEDIHTSR--------------VVAHVLPCG----H-LLHRTCYEEMLKEGY--------RCPL--CSGP 46
2JRJA 1 NCPICLEDIHTSR--------------VVAHVLPCG----H-LLHRTCYEEMLKEGY--------RCPL--CMHS 46
1IYMA 1 ECAVCLAELEDGE--------------EARFLPRCG----H-GFHAECVDMWLGSHS--------TCPL--CRLT 46
1V87A 1 DCIICMEKLAVASGYSDMTDSKALGPMVVGRLTKCS----H-AFHLLCLLAMYCNGNKD---GSLQCPS--CKTI 65
2VJEA 1 PCVICQGRPKN------------------GCIV-HGKT-GHLMACFTCAKKLKKRNK--------PCPV--CRQP 45
2VJEB 1 PCSLCEKRPRD------------------GNII-HGRT-GHLVTCFHCARRLKKAGA--------SCPI--CKKE 45
2D8SA 1 ICRICHCEGDDE---------------SPLITPCH-CTGSLHFVHQACLQQWIKSSD------TRCCEL--CKYE 51
1VYXA 1 VCWICNEELGN------------------ERFRACGCTGELENVHRSCLSTWLTISRN------TACQI--CGVV 49
2CT0A 1 ICNICHSLLIQG--------------------QSCETCG-I-RMHLPCVAKYFQSNAE------PRCPH--CNDY 45
2CSZA 1 TCARCQESLGRLSPKT----------------NTCRGCN-H-LVCRDCRIQESNGTW--------RC--KVCSGP 47
2YURA 1 LCLICKDIMTD------------------AVVIPC--CG--NSYCDECIRTALLESD------EHTCPT--CHQN 45
1UR6B 1 ECPLCMEPLEIDD----------------INFFPCT-CG--YQICRFCWHRRTDENG--------LCPA--CRKP 46
1WIMA 1 GCKLCLGEYPVEQ---------------MTTIAQCQ-C----IFCTLCLKQYVELLIKEGLETAISCPDAACPKQ 53
1WEOA 1 FCEICGDQIGLTVE--------------GDLFVACNECG--FPACRPCYEYERREGT-------QNCPQ--CKTR 50
Consensus_aa: C lC h C C Hh hC Ch p CPh Cp
Conservation: 9 9 8 5 8 7 9 97 9
C3HC4-type
C3H2C3-type
C2H2C4-type
C4HC3-type
C4C4-type

**B: C6H3C2D-type (C6H2C4-type) and RING/U-box**

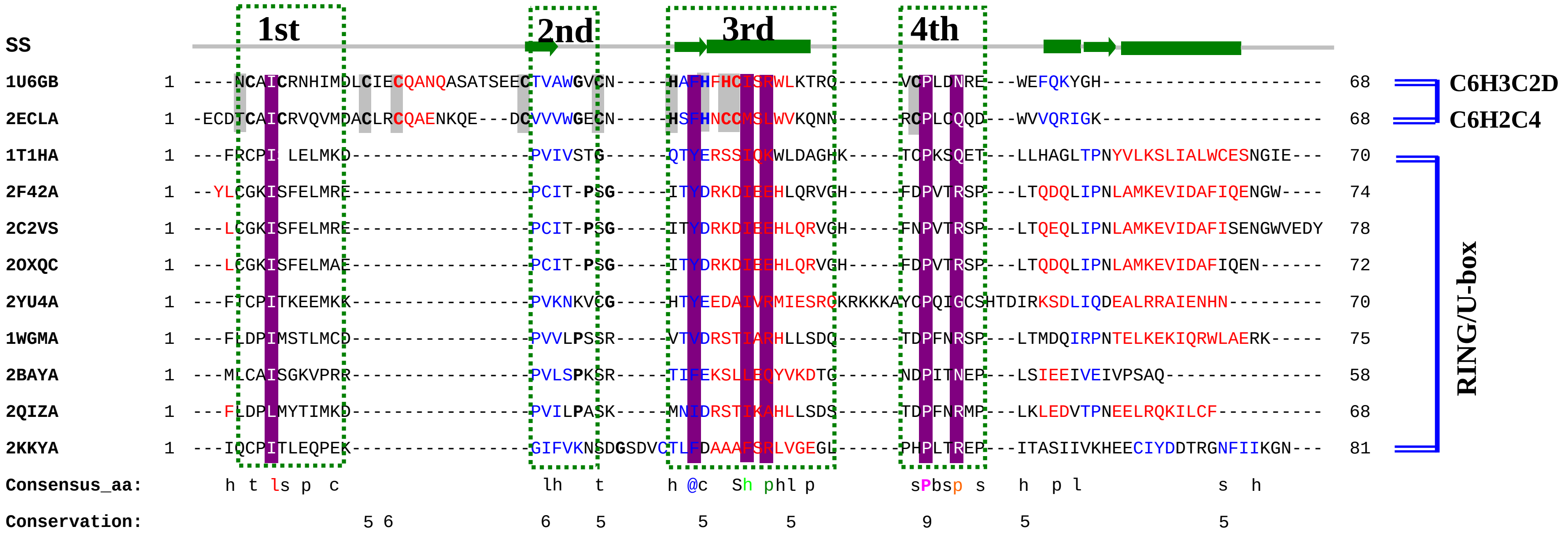


**Figure S3. Multiple sequence and structure alignments of the solved RING domains. A:** C3HC4-type, C3H2C3-type (RING-H2), C3HC3D-type, C4HC3-type (RINGv) and C4C4-type; **B:** C6H3C2D-type/ C6H2C4-type and U-box. Name of sequences were indicated by PDB IDs. Secondary structures of the sequences were colored according to experimentally-determined structural data (red letters: alpha-helix, blue letters: beta-strand). Four structurally conserved regions (SCRs) (1st: N-loop; 2nd: the first β-sheet region; 3rd: βα-region and 4th: C-loop) were indicated by green rectangles. Except from 1BORA and 2CSZA, all the others have a similar second structural arrangement of the ββα motif. The top lines of the alignments indicate the consensus secondary structure (SS); Conserved *Cys/His* residues binding zinc ions were shadowed by grey. Consensus amino acids were showed by pansy for easy identification. One exception is 3I2D with one occurrence of zinc ion at 3, 5, 7 and 8 positions, its corresponding site residues do not bind atom of zinc were shadowed by yellow (1, 2, 4 and 6 positions). *Gly/Pro* residues in short loop between β hairpin were indicated by bold letters for easy identification. The 4th metal-chelating residue position and zinc ion coordinating amino acids tend to be changeable in distinct type RING domains. The last 2 lines in different types showed consensus amino

acid sequence (Consensus_aa) and conservation indices for positions with a conservation index above 4. Consensus amino acid symbols are: conserved amino acids are in bold and uppercase letters; aliphatic (I, V, L): l; aromatic (Y, H, W, F): @; hydrophobic (W, F, Y, M, L, I, V, A, C, T, H): h; alcohol (S, T): o; polar residues (D, E, H, K, N, Q, R, S, T): p; tiny (A, G, C, S): t; small (A, G, C, S, V, N, D, T, P): s; bulky residues (E, F, I, K, L, M, Q, R, W, Y): b; positively charged (K, R, H): +; negatively charged (D, E): -; charged (D, E, K, R, H): c.

**Reference database for Figure S3:**

1 APSSP: http://imtech.res.in/raghava/apssp/

2 NCBI: http://www.ncbi.nlm.nih.gov/

3 PDB: http://www.pdb.org/

4 Promals3D: http://prodata.swmed.edu/promals3d/
