# Supplementary material for: Comprehensively Surveying Structure and Function of RING Domains from Drosophila melanogaster
Source: PLoS One. 2011 Sep 2;6(9):e23863. doi: 10.1371/journal.pone.0023863 (PMC3166285; doi:10.1371/journal.pone.0023863)
Supplement: Figure S4 — Sequence alignment of orthologous Diap2. All the orthologous Iap2/Diap2 from arthropods possess a tandem repeat of 3 BIR domains and 1 RING domain. Apart from a tandem repeat of 3 BIR domains and 1 RING domain, all orthologous Iap2/Diap2 from vertebrates acquired an additional CARD domain. Iap2/Diap2 from arthropods lack the corresponding CARD domain, which were indicated by broken line rectangle. Hom, Homo sapiens; Pan, Pan troglodytes; Mac, Macaca mulatta; Pon, Pongo abelii; Mus, Mus musculus; Rat, Rattus norvegicus; Orn, Ornithorhynchus anatinus; Mon, Monodelphis domestica; Bos, Bos taurus; Equ, Equus caballus; Can, Canis lupus familiaris; Ory, Oryctolagus cuniculus; Dan, Danio rerio; Gal, Gallus gallus; Xen, Xenopus (Silurana) tropicalis; Dro, Drosophila melanogaster; Drp, Drosophila pseudoobscura; Tri, Tribolium castaneum; Api, Apis mellifera; Nas, Nasonia vitripennis. (PDF) [file pone.0023863.s004.pdf]

Ying et al. Figure S4

The first BIR domain(BIR1)

```
NP_001156 Hom      MNIVENSIFLSNLMKSANTFELKYDLSCELYRMSTYSTFPAGVPVSERSLARAGFYYTGVNDKVKCFCCGLMLDNWKRGD
XP_508719 Pan      MNIVENSIFLSNLMKSANTFELKYDLSCELYRMSTYSTFPAGVPVSERSLARAGFYYTGVNDKVKCFCCGLMLDNWKRGD
XP_001095970 Mac   MNIVENSIFLSNLMKSANTFELKYDLSCELYRMSTYSAFPAGVPVSERSLARAGFYYTGVNDKVKCFCCGLMLDNWKRGD
XP_002822439 Pon   MNIVENSIFLSNLMKSANTFELKYDLSCELYRMSTYSTFPAGVPVSERSLARAGFYYTGVNDKVKCFCCGLMLDNWKRGD
NP_031490 Mus      MNMVQDSAFLAKLMKSADTFELKYDFSCELYRLSTYSAFPRGVPVSERSLARAGFYYTGANDKVKCFCCGLMLDNWKQGD
NP_076477 Rat      MNMVQGSAFLAKLMKSADTFELKYDFSCELYRLSTYSTFPAGVPVSERSLARAGFYYTGVNDKVKCFSCGLMLDNWKQGD
XP_001509526 Orn   MNVVANSMFLSNLMNGNSGYELKYDFSCELFRMSTYSTFPTNVPVSERSLARAGFYYTGASDKVKCFSCGLMLDNWKPGD
XP_001362624 Mon   MSVVEDGVLLSHLMSS-DTHEMKYDLSCELYRMSTYSAFPVNVPISERSLARAGFYYTGVNDRVKCFCCGLMLDNWKQGD
NP_001030370 Bos   MNIVENSVFLSNLMKSANMFELKYDFSCELYRMSTYSTFPAGVPVSERSLARAGFYYTGVKDKVKCFCCGLMLDNWKQGD
XP_001499925 Equ   MNIVENSVFLSNLMKSANMFELKYDFSCELYRISTYSTFPPGVPVSERSLARAGFYYTGINDRVKCFCCGLMLDNWKQGD
NP_001074194 Can   MNIVQNSIFLSNLLRSSPVFELKYDHSCELYRMSTYSTFPAGVPVSERSLARAGFYYTGAKDRVRCFCCGLMLDNWKAGD
XP_002708629 Ory   MNIVENSIFLANLMKSANTFELKYDFSCELYRMSTYSTFPAGVPVSERSLARAGFYYTGVNDKVKCFCCGLMLDNWKQGD
XP_002666015 Dan   MEILQNSAFLRGLCRTSGPADLQYDNSSELFRISTYAKFPTTAAVTERSLARAGFYYTGLGDRVQCFRCNVTADNWQSGD
NP_001007823 Gal   NIMDSSPLLASVMKQNAHCGELKYDLSCELYRMSTFSTFPVNVPVSERRLARAGFYYTGVQDKVKCFSCGLVLDNWQPGD
NP_001005449 Xen   PSMCKITL----------------EFSCELYRLSTFSTFPSNTHVSERNLAKAGFYYTGQDDKVKCFTCGLMLDNWKKGD
NP_477127 Dro      -------L----------------GMELESVRLATFGEWPLNAPVSAEDLVANGFFATGNWLEAECHFCHVRIDRWEYGD
XP_001361948 Drp   -------L----------------RLELESVRTATFVQWPLNAPVSAEDLVTNGFFATGNWLEAECNWCHVRIDRWEYGD
XP_975027 Tri      -------------------------MNVEQNRLDTFEEWPQDAAVSPPRIAKAGFFYTKHDVTVECFSCHLTISEWNYGD
XP_396819 Api      -------------------------MNIEKNRLQTFTDWPANAAVDAVRIAKAGFYYSGHGLEVQCFLCGVKISDWNYGD
XP_001606042 Nas   ---------------------LRKEFVEEVNRLRTFLDWPANCPVSTARIAKAGFYYTGTAQIAQCFLCGTRVSEWNFGD
```

The first BIR domain(BIR1)

```
NP_001156 Hom       SPTEKHKKLYPSCRFVQSLNSVNNLEATSQPTF---PSSVTNSTH---SLLPGTENSGYFRGSYSNS-----------PS
XP_508719 Pan       SPIEKHKKLYPSCRFVQSLNSVNNLEATSQPTF---PSSVTNSTH---SLLPGTENSGYFGGSYSNS-----------PS
XP_001095970 Mac    SPVEKHKKLYPSCRFVQSLNSVNNSEATSQPAF---PSSVTNSTH---SLLPGTENSGYFSGSYSSF-----------PS
XP_002822439 Pon    SPIEKHKKLYPSCRFVQSLNSVNNLEATSQPTF---PSSVTNSTH---SLLPGTENSGYFSGSYSSF-----------PS
NP_031490 Mus       SPMEKHRKLYPSCNFVQTLNPANSLEASPRPSL---PSTAMSTMP---LSFASSENTGYFSGSYSSF-----------PS
NP_076477 Rat       SPIEKHRKLYPSCSFVQTLNPANSLEASPQPPL---PSTATSTMP---SGFAASESTGYFSGSYSSF-----------PS
XP_001509526 Orn    SAIEKHKQLYPSCSFIQNLLQTHNPGASSYSAFCPPPLGSLSPTA---TISPSLEPSGYFSGSFSSF-----------PL
XP_001362624 Mon    NAIDKHKQLYPSCAFIQNLISVNLG--SSQSTF---SSSVNNATR---SLSLSSEQSGYFSGSYSSF-----------PL
NP_001030370 Bos    NPIEKHKKLYPSCSFVQNLNSINVSGASSQPTF---HSSLTNSTH---SLLPSLENSGYFSGSYSSF-----------PS
XP_001499925 Equ    NPTEKHKKLYPSCSFVQNLNSVNISGATSQLAF---SSSVTNSTH---SLLPSLENSGYFSGSYSSF-----------PS
NP_001074194 Can    SPTGKHRNLYPSCSFIQNLSAVSTVGAASQPPA---PLSEARSTH---AQSPGLERSSYFSASYSSF-----------PV
XP_002708629 Ory    KPIEKHRKLYPSCSFVQSLNSVNNWEANSRSTF---PSSVTNSTH---SFLPSWEHSGYYSGSYSSL-----------PS
XP_002666015 Dan    CPAERHKQLSPNCSFIQSLPATANLLSSSHSAFSPLRNVAVLQLSAPATAAPSTTAPSTSGQTEEQVGYLNMGFTNLAPS
NP_001007823 Gal    NAMEKHKQVYPSCSFVQNMLSLNNLGLSTHSAFSPLVASNLSPSLRSMTLSPSFEQVGYFSGSFSSF-----------PQ
NP_001005449 Xen    NAFEKHKKLYPSCSFIQNVPSVNLG--ASLYSAFSPPASNSTPMP---AASAENDKVEAITLKYSSI-----------PQ
NP_477127 Dro       QVAERHRRSSPICSMVLAPNHCGNV-------------------------------------------------------
XP_001361948 Drp    QVAERHRRCSPICSMVLAPSHCGNI-------------------------------------------------------
XP_975027 Tri       QVMAKHKTLNPSCPFVLNPTTSGNV-------------------------------------------------------
XP_396819 Api       QAIVRHRLAEPNCSFVQNPSSTCNI-------------------------------------------------------
XP_001606042 Nas    QAMALHRIANPECPFVLDPIATCNV-------------------------------------------------------
```

The second BIR domain(BIR2)

```
NP_001156 Hom      NPVNSRANQDFSALMRSS-------------------------YHCA MNNENARLLTFQTWPL-TFLSPTDLAKAGFYYI
XP_508719 Pan      NPVNSRANQDFSALMRSS-------------------------YHCA MNNENARLLTFQTWPL-TFLSPTDLAKAGFYYI
XP_001095970 Mac   NPVNSRANQDFSALMRSS-------------------------YHCA MNNEKARLLTFQTWPL-TFLSPTDLAKAGFYYV
XP_002822439 Pon   NPVNSRVNQDFSALMRSS-------------------------YHCA MNNENARLLTFQTWPL-TFLSPTDLAKAGFYYI
NP_031490 Mus      DPVNFRANQDCPALSTSP-------------------------YHFA MNTEKARLLTYETWPL-SFLSPAKLAKAGFYYI
NP_076477 Rat      DPVNFRANQDCPAFSTSP-------------------------YHFA MNTEKARLLTYQTWPL-SFLSPAELAKAGFYYT
XP_001509526 Orn   DPVTSRAIEDLSQPR---------------------Phvd----NSA MSSEEARICTFQSWPL-TFLSPSALAKAGFYYT
XP_001362624 Mon   EPVTSRGVEDLSPLNTSP-------------------------YSYS MSTEEARILTYQAWPL-TFLSPLDLARAGFYYI
NP_001030370 Bos   NLLNSGPNQDFSALRISS-------------------------YPCA MNTEKARLLTFQMWPL-TFLSPTDLAKAGFYYI
XP_001499925 Equ   NPVNSGPNHDFSALRTSP-------------------------YHCA MNTEKARLLTFQMWPL-TFLSPTDLAKAGFYYI
NP_001074194 Can   DPVDFRPSPAMSPWRAGP-------------------------SCVT MKSEEDRLCTFQGWPL-AFPLPSALARAGFYYV
XP_002708629 Ory   NPVNSRANQDFSAFRVSP-------------------------YHSA MSTEKARLLTFQSWPL-TFLSPMDLAKAGFYYV
XP_002666015 Dan   SPISSRGVEDMSHQR---------------------PPA---CHNPG MRREQERLDTFQNWTL-ATVTPAELAKAGLYYL
NP_001007823 Gal   DPVTTRAAEDLSHLRSKL-------------------------HNPS MSTEEARLRTFHAWPL-MFLSPAELAKAGLYYL
NP_001005449 Xen   DPVTLRGIEDLSHVRISE-------------------------Y--- MYTEEARLNSFQEWSN-MFLTPAELAKAGFYFV
NP_477127 Dro      --------PRS------------QESDNEGNSVVDSPES---CSCPD LLLEANRLVTFKDWPN-PNITPQALAKAGFYYL
XP_001361948 Drp   --------TLS------------QENDSEGNTMVDGGGTASPCACPD LHLEANRLETFKDWPN-PNVTPQALAKAGFYYL
XP_975027 Tri      --------PIISPTNVPS-------------------------TSDS MKDEAVRLKTFAKWPKPHIVAPERLARAGFYYL
XP_396819 Api      --------PLIPINNNELPSSSTEISQNNIIEYQSTNSYQYKESQKK YRIMSHRLQSFTNWPLSSVILPENLAKAGFYYL
XP_001606042 Nas   --------PLILMADESINPNSIDNNHNENSESSAGGVTQLENAITD LARYSHRLNTFRNWPIPAIVSPERLARSGFYYL
```

The second BIR domain(BIR2)

```
NP_001156 Hom     GPGDRVACFACGGKLSNWEPKDNAMSEHLRHFPKCPFIENQLQDTSRY------TVSNLSMQ------------------
XP_508719 Pan     GPGDRVACFACGGKLSNWEPKDNAMSEHLRHFPKCPFIENQLQDTSRY------TVSNLSMQ------------------
XP_001095970 Mac  GPGDRVACFACGGKLSNWEPKDNAMSEHLRHFPKCPFIENQLQDTSRY------TVSNLSMQ------------------
XP_002822439 Pon  GPGDRVACFACGGKLSNWEPKDNAMSEHLRHFPKCPFIENQLQDTSRY------TVSNLSMQ------------------
NP_031490 Mus     GPGDRVACFACDGKLSNWERKDDAMSEHQRHFPSCPFLKDLGQSASRY------TVSNLSMQ------------------
NP_076477 Rat     GPGDRVACFACGGKLSNWDRKDDPLSEHRRHFPSCPFLKDVGQFTSQY------TVSNLSMQ------------------
XP_001509526 Orn  GPGDRVACFTCGGKLSNWEPKDDAMSEHRRHFPGCPFLERQTRDASRF------NVSNASMQ------------------
XP_001362624 Mon  GPGDMVACFACGGKLSNWEPKDDAMSEHRRHFPHCPFLESQIQETSRF------NVSNLSMQ------------------
NP_001030370 Bos  GPGDRVACFACGGTLSNWEPKDDAMSEHLRHFPNCPFLGNQLQGTSRY------TASNLSMQ------------------
XP_001499925 Equ  GPGDRVACFACGGKLSNWEPKDDAMSEHLRHFPNCPFVENQFEDTSRY------TVSNLSMQ------------------
NP_001074194 Can  GPGDRVACFACGGKLSNWEPDDDALSEHLRHFPDCPFVEGQLQATVRY------TASNLSMQ------------------
XP_002708629 Ory  GPGDRVACFACGGKLSNWEPKDDAMSEHLRHFPNCPFVENQLQDASRY------TISNLSMQ------------------
XP_002666015 Dan  GQGDRVACFSCGGQLGSWEPGDRAVSEHQRHYPNCRFVRGDRADNIPLSGGGLSNVSNSAMQ------------------
NP_001007823 Gal  GTADKVACFTCGGQLSNWEPKDNAMSEHRRHFPNCPFVENLMRDQPSF------NVSNVTMQ------------------
NP_001005449 Xen  GPGDKVACFTCDGKLNNWEPNDNAMSEHRRHFPNCPFVKSSTRVSSRF------SVSNVSMQ------------------
NP_477127 Dro     NRLDHVKCVWCNGVIAKWEKNDNAFEEHKRFFPQCPRVQMGPL--------------------IEFATGKNLDELGIQPT
XP_001361948 Drp  NRLDHVKCVWCNGVIAKWEKNDNAFDEHRRFFPNCPRVQMGPL--------------------IEFAAGKNLEELGIQPT
XP_975027 Tri     NTGDNTKCAFCKGVVRAWEPGDDPDQEHKRHFEDCPFVLTEIVPRLSQNTNGQVKTDSSSFTNLHLVANENLHCLGVQTH
XP_396819 Api     QRDDEVQCIYCGGILKKWELGDDPNKKHRKYFPDCNFYVYQDKDDNLY-----LSNVKLISG-----PTSNLSDLGIQTH
XP_001606042 Nas  QQADMVECAYCQGVILKWEPGDDPDREHRIHFPNCDFYMRDGAAYDVSAEKVELGNVKLMPG-----TTSNFTELGIQHH
```

The third BIR domain(BIR3)

```
NP_001156 Hom      ---------THAARFKTFFNWPSSVLVNPEQLASAGFYYVGNSDDVKCFCCDGGLRCWESGDDPWVQHAKWFPRCEYLIR
XP_508719 Pan      ---------THAARFKTFFNWPSSVLVNPEQLASAGFYYVGNSDDVKCFCCDGGLRCWESGDDPWVQHAKWFPRCEYLIR
XP_001095970 Mac   ---------THAARFKTFFNWPSSVLVNPEQLASAGFYYVGNSDDVKCFCCDGGLRCWESGDDPWVEHAKWFPRCEYLIR
XP_002822439 Pon   ---------THAARFKTFFNWPSSVLVNPEQLASAGFYYVGNSDDVKCFCCDGGLRCWESGDDPWVQHAKWFPRCEYLIR
NP_031490 Mus      ---------THAARIRTFSNWPSSALVHSQELASAGFYYTGHSDDVKCFCCDGGLRCWESGDDPWVEHAKWFPRCEYLLR
NP_076477 Rat      ---------THAARVRTFSTWPSSALVHPQELASAGFYYTGHSDDVKCFCCDGGLRCWESGDDPWVEHAKWFPRCEYLIR
XP_001509526 Orn   ---------THAARVKTFLNWPARIPVQPEQLASAGFYYVGRNDDVKCFCCDGGLRCWESGDDPWIEHAKWFPRCEYMIR
XP_001362624 Mon   ---------THAARVKTFVTWPPQIPVHPEQLANAGFYYVGRNDDVKCFCCDGGLRCWESGDDPWVEHAKWFPRCEYLIR
NP_001030370 Bos   ---------TYAARIKTFCSWPSSVPVHPEQLASAGFYYVGHSDDVKCFCCDGGLRCWESGDDPWVEHAKWFPRCEYLIQ
XP_001499925 Equ   ---------THAARFRTFCNWPSSVQVPPEQLASAGFYYMGHSDDVKCFCCDGGLRCWESGDDPWVEHAKWFPRCEYLIR
NP_001074194 Can   ---------TLAARSRTFCNWPPRAPVHPEQLASAGFYYMGHSDDVKCFCCDGGLRCWESGDDPWVEHAKWFPRCEYLIR
XP_002708629 Ory   ---------THAARFKTFFNWPSSLLVHPEQLASAGFYYVGHSDDVKCFCCDGGLRCWELGDDPWVEHAKWFPRCEYLIR
XP_002666015 Dan   ---------QCEERLLTFVNWPSRIPVRPDQLAKAGFYYVGRNDDVKCFCCDGGLRCWESGDDPWVEHAKWFPRCEYLLQ
NP_001007823 Gal   ---------THEARVKTFINWPTRIPVQPEQLADAGFYYVGRNDDVKCFCCDGGLRCWESGDDPWIEHAKWFPRCEYLLR
NP_001005449 Xen   ---------ASSARLKTFASWPPRIPISPTRLAEAGFYYVGRNDDVKCFCCDGGLRCWESGDDPWVEHAKWFPRCEYLLH
NP_477127 Dro      TLPLRPKYACVDARLRTFTDWPISNIQPASALAQAGLYYQKIGDQVRCFHCNIGLRSWQKEDEPWFEHAKWSPKCQFVLL
XP_001361948 Drp   TQPKLPNFACVDSRLRTFTDWPIGNIQPPEPLAQAGLFYQKIADQVRCFHCNIGLRSWQKEDEPWHEHAKWSPKCQFVLL
XP_975027 Tri      KGPKKPNYATLESRLRSFATWPPDLIQTPDILSQAGFYYEGMGDQVRCFHCDGGLRHWDPQDDPWTEHARWFPRCSFIKL
XP_396819 Api      TTPKKQDCATYEGRLHTFNGWPENIKQTPEILASAGFYYDGYSDHVRCFHCDGGLRNWETTDDAWIEHAKWFPKCEFVNL
XP_001606042 Nas   SAPRQPKHATYEGRLRTFQGWPSNLRQTPEMLADAGFYYVGAQDQVRCFHCDGGLRNWEETDDAWIEHARWFPKCGYVAL
```

```
NP_001156 Hom    IKGQEFIRQVQASYPHLLEQLLSTSDSPGDENAESSIIHFEPGEDHSEDAI-------MMNTPVINAAVEMGFSRSLVKQ
XP_508719 Pan    IKGQEFIRQVQASYPHLLEQLLSTSDSPRDENTESSIIHFEPGEDHSEDAI-------MMNTPVVNAAVEMGFSRSLVKQ
XP_001095970 Mac IKGQEFIRQVQASYPHLLEQLLSTSDSPEDENAESSIIRFEPGEDHSEDAI-------MMNTPVVNAAVEMGFSRSLVKY
XP_002822439 Pon IKGQEFIRQVQASYPHLLEQLLSTSDSPGDENAESSIIRFEPGEDHSEDAI-------MMNTPVVNAAVEMGFSRSLVKQ
NP_031490 Mus    IKGQEFVSQVQAGYPHLLEQLLSTSDSPEDENADAAIVHFGPGES-SEDVV-------MMSTPVVKAALEMGFSRSLVRQ
NP_076477 Rat    IKGQEFVGQVQAGYPHLLEQLLSTSDSPEDETGEAAIVHLGPGEN-WEDAV-------MMNTPVVKAALDMGFSRSLVRQ
XP_001509526 Orn MKGQEFVNQIQARYPHLLEQLLSTSDTPVDESADPPIIHFGPGENPAEDAI-------MMSNPVVKAALEMGFSRRLVKQ
XP_001362624 Mon MKGQEFVDQVQARYPHLLEQLLSTSDIPGDENAELPIIHFGPGENNSEDTV-------MMNTPVIKAALEMGFSRSLVKQ
NP_001030370 Bos IKGQEFISRVQASYPHLLEQLLSTSDNPEDENTESPIIHFGPGENHSEDSV-------MMNTPVVKAALEMGFSRRLVKQ
XP_001499925 Equ IKGQEFISRVQANYPHLLEQLLSTSDNPEDENAESPIIHFGPGENHSEDAV-------MMNTPVVKAALEMGFSRRLIKQ
NP_001074194 Can IKGQEFISQIQASYPHLLEQLLSTSDNTEDENTESPIVHFGPGEYHSEDAV-------MMNTPVVMAALEMGFSRSLVRQ
XP_002708629 Ory IKGQEFISRVQASYPHLLEQLLSTSDTPEDENAEPPIVHFGPGENPSEDAV-------MMNTPVVKAALDMGFSRRLVKQ
XP_002666015 Dan EKGQEFVHQIQARFPRLFEQLLTNGDSNSREFVDPPVVHLGPGEDRSEDAV-------MMNNPVVKSALEMGFERGLVKQ
NP_001007823 Gal VKGGEFVSQVQARFPHLLEQLLSTSDTPVDENMD-PIIHFEPGESPSEDAI-------MMNTPVVKAALEMGFSRRLIKQ
NP_001005449 Xen IRGQDYVQEVQERYPHLLDQLLSSSENQNEAKNI-PIIRLGTENSQ-EDEI-------MMSTPMVQTALEIGFNRRLVKR
NP_477127 Dro    AKGPAYVSEVLATTAANASSQPATAPAPTLQADV------------------------LMDEAPAKEALALGIDGGVVRN
XP_001361948 Drp AKGPAFVREVGEAMAASSGSQIATAPAL--------------------------QLNALMDESPAKEALALGINGGVLRN
XP_975027 Tri    VKGQEFVTACSLELNVNSNLEEVDQKNYPSVQSRK-------------REVTEQEIQEHMLGEVALSALSIGLNVERVKR
XP_396819 Api    VRGQEFIKQCINNRPPLDQSIFEDKTEDQNIDKIEMSHSSSSLEITEATLKK------LLESPLVTVALEIGLHIDRVKR
XP_001606042 Nas VRGQDFIKHCIEHRPPLDPAILGVPDENTDIYTTPPVSPAVEPSQLPIRQVTDAELDSLLGSAPAVAALEIGLHVGRVKM
```

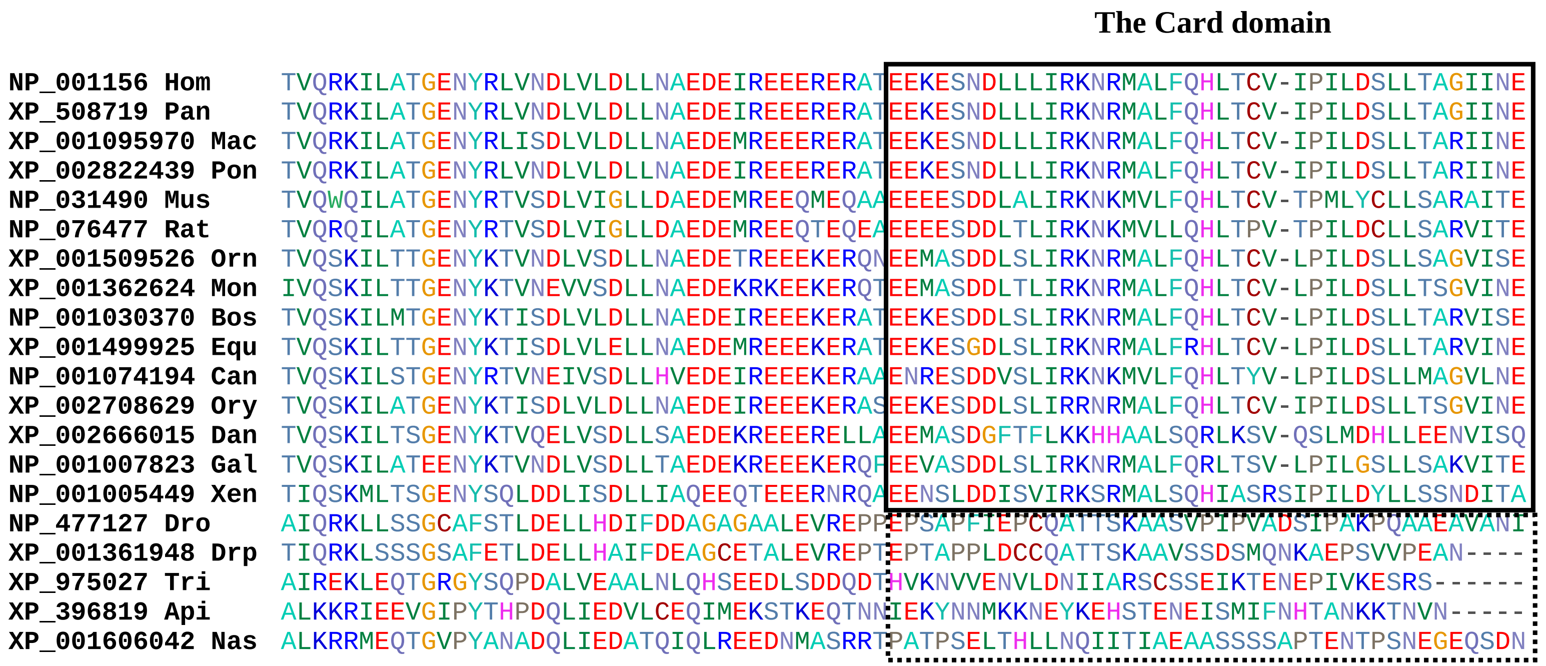

The Card domain
NP_001156 Hom TVQRKILATGENYRLVNDLVLDLLNAEDEIREEERERATEEKESNDLLLIRKNRMALFQHLTCV-IPILDSLLTAGIINE
XP_508719 Pan TVQRKILATGENYRLVNDLVLDLLNAEDEIREEERERATEEKESNDLLLIRKNRMALFQHLTCV-IPILDSLLTAGIINE
XP_001095970 Mac TVQRKILATGENYRLISDLVLDLLNAEDEMREEERERATEEKESNDLLLIRKNRMALFQHLTCV-IPILDSLLTARIINE
XP_002822439 Pon TVQRKILATGENYRLVNDLVLDLLNAEDEIREEERERATEEKESNDLLLIRKNRMALFQHLTCV-IPILDSLLTARIINE
NP_031490 Mus TVQWQILATGENYRTVSDLVIGLLDAEDEMREEQMEQAAEEEESDDLALIRKNKMVLFQHLTCV-TPMLYCLLSARAITE
NP_076477 Rat TVQRQILATGENYRTVSDLVIGLLDAEDEMREEQTEQEAEEEESDDLTLIRKNKMVLLQHLTPV-TPILDCLLSARVITE
XP_001509526 Orn TVQSKILTTGENYKTVNDLVSDLLNAEDETREEEKERQNEEMASDDLSLIRKNRMALFQHLTCV-LPILDSLLSAGVISE
XP_001362624 Mon IVQSKILTTGENYKTVNEVVSDLLNAEDEKRKEEKERQTEEMASDDLTLIRKNRMALFQHLTCV-LPILDSLLTSGVINE
NP_001030370 Bos TVQSKILMTGENYKTISDLVLDLLNAEDEIREEEKERATEEKESDDLSLIRKNRMALFQHLTCV-LPILDSLLTARVISE
XP_001499925 Equ TVQSKILTTGENYKTISDLVLELLNAEDEMREEEKERATEEKESGDLSLIRKNRMALFRHLTCV-LPILDSLLTARVINE
NP_001074194 Can TVQSKILSTGENYRTVNEIVSDLLHVEDEIREEEKERAAENRESDDVSLIRKNKMVLFQHLTYV-LPILDSLLMAGVLNE
XP_002708629 Ory TVQSKILATGENYKTISDLVLDLLNAEDEIREEEKERASEEKESDDLSLIRRNRMALFQHLTCV-IPILDSLLTSGVINE
XP_002666015 Dan TVQSKILTSGENYKTVQELVSDLLSAEDEKREEERELLAEEMASDGFTFLKKHHAALSQRLKSV-QSLMDHLLEENVISQ
NP_001007823 Gal TVQSKILATEENYKTVNDLVSDLLTAEDEKREEEKERQFEEVASDDLSLIRKNRMALFQRLTSV-LPILGSLLSAKVITE
NP_001005449 Xen TIQSKMLTSGENYSQLDDLISDLLIAQEEQTEEERNRQAEENSLDDISVIRKSRMALSQHIASRSIPILDYLLSSNDITA
NP_477127 Dro AIQRKLLSSGCAFSTLDELLHDIFDDAGAGAALEVREPPEPSAPFIEPCQATTSKAASVPIPVADSIPAKPQAAEAVANI
XP_001361948 Drp TIQRKLSSSGSAFETLDELLHAIFDEAGCETALEVREPTEPTAPPLDCCQATTSKAAVSSDSMQNKAEPSVVPEAN----
XP_975027 Tri AIREKLEQTGRGYSQPDALVEAALNLQHSEEDLSDDQDTHVKNVVENVLDNIIARSCSSEIKTENEPIVKESRS------
XP_396819 Api ALKKRIEEVGIPYTHPDQLIEDVLCEQIMEKSTKEQTNNIEKYNNMKKNEYKEHSTENEISMIFNHTANKKTNVN-----
XP_001606042 Nas ALKRRMEQTGVPYANADQLIEDATQIQLREEDNMASRRTPATPSELTHLLNQIITIAEAASSSSAPTENTPSNEGEQSDN

The Card domain

```
NP_001156 Hom      QEHDVIKQKTQTSLQARELIDTILVKGNIAATVFRNSLQEAEAVLYEHLFVQQDIKYIPTEDVSDLPVEEQLRRLQEERT
XP_508719 Pan      QEHDVIKQKTQTSLQARELIDTILVKGNIAATVFRNSLQEADAVLYEHLFVQQDIKYIPTEDVSDLPVEEQLRRLQEERT
XP_001095970 Mac   QEHDVIKQKTQTSLQARELIDTILVKGNIAATVFRNSLQEADAVLYERLFVQQDIKYIPTEDVSDLPVEEQLRRLQEERT
XP_002822439 Pon   QEHDVIKQKTQTSLQARELIDTILVKGNIAATVFRNSLQEADAVLYEHLFVQQDIKYIPTEDVSDLPVEEQLRRLQEERT
NP_031490 Mus      QECNAVKQKPHT-LQASTLIDTVLAKGNTAATSFRNSLREIDPALYRDIFVQQDIRSLPTDDIAALPMEEQLRKLQEERM
NP_076477 Rat      QEYDAVKQKPHT-LQARTLIDTVLAKGNTAATSFRNSLQEIDPGLYRDIFVRQNIRSLPTDDIAALPMEEQLRKLQEERT
XP_001509526 Orn   QERDVIKQKTQTSLQARELIDTVLVKGNAAANIFKNCLKEDDCILYKNLFVEKNMKYIPTEDVSGLPMEEQLRRLQEERT
XP_001362624 Mon   QEHDVIKQKTQTSLQARELIDTILVKGNAAASVFKNCLREIGSVLYNNLFVEKNMKYIPTEDVSGLPMEEQLRRLQEERT
NP_001030370 Bos   QEHDVIKQKTQTSLQARELIDTILVKGNFAATIFKNSLQEIDPMLYKRLFVQQDIKYIPTENVSDLPMEEQLRRLQEERT
XP_001499925 Equ   QEYDVIKQKTQTSLQSRELIDTILVKGNFAATIFKNSLQEIEPTLYKHLFVQQDIKYIPTEDVSDLPMEEQLRRLQEERT
NP_001074194 Can   QEHSSIKQKARTSLQARELIDTVLVKGSSAVTIFKNSLQEIDPMLYKRFFVQQDRKYIPTEDISDLPVEEQLRRLQEERT
XP_002708629 Ory   QEHAVIKQKTQTSLQARELIDIILVKGNIAATVFKDCLQETDPVLYKHLFVQQDIKYIPTEDVSDLPMEEQLRRLQEERT
XP_002666015 Dan   KEYDSIRNCTSVKQQTGQLIDLVLSKGNAAAEVFRNWIKKNDVYLLRELMAQTNEAASPSQDLSDLPMEEQLRRLQEERT
NP_001007823 Gal   LEHDVIKQKTQTPLQARELIDTVLVKGNAAASIFRNCLKDCDPVLYKDLFVEKSMKYVPTEDVSGLPMEEQLRRLQEERT
NP_001005449 Xen   AEYEAIKLKTQPILQAQEMIETIITKGKTAVQTLKNCLQKHDPNLFRQLFTEQSLKCISLDDYSDLSMEEQLRRLQEERT
NP_477127 Dro      SKITDEIQKMSVATPN-----------------------------------------G------NLSLEEENRQLKDARL
XP_001361948 Drp   --ISEQMQRMSVAPPN-----------------------------------------G------NISLEEENRQLKDARL
XP_975027 Tri      ---------------N-----------------------------------------K------TLSLEEENRILKEARL
XP_396819 Api      -------------------------------------------------------FKE------ITSLEEENRKLKEARL
XP_001606042 Nas   IQRRRRRRPPHEVESETQTDADERTVNAEKAGKLVILSFVHLTNLLINVVKLKKIFSK------TVALEEENRRLKEARQ
```

The RING domain

```
NP_001156 Hom     CKVCMDKEVSIVFIPCGHLVVCKDCAPSLRKCPICRSTIKGTVRTFLS
XP_508719 Pan     CKVCMDKEVSIVFIPCGHLVVCKDCAPSLRKCPICRSTIKGTVRTFLS
XP_001095970 Mac  CKVCMDKEVSIVFIPCGHLVVCKDCAPSLRKCPICRSTIKGTVRTFLS
XP_002822439 Pon  CKVCMDKEVSIVFIPCGHLVVCKDCAPSLRKCPICRSTIKGTVRTFLS
NP_031490 Mus     CKVCMDREVSIVFIPCGHLVVCKDCAPSLRKCPICRGTIKGTVRTFLS
NP_076477 Rat     CKVCMDREVSLVFIPCGHLVVCKECAPSLRKCPICRGTIKGTVRTFLS
XP_001509526 Orn  CKVCMDKEVSIVFIPCGHLVVCKECAPSLRKCPICRGTIKGTVRTFLS
XP_001362624 Mon  CKVCMDKEVSIVFIPCGHLVVCKECAPSLRKCPICRGIIKGTVRTFLS
NP_001030370 Bos  CKVCMDKEVSIVFIPCGHLVVCKDCAPSLRKCPICRGTIKGTVRTFLS
XP_001499925 Equ  CKVCMDKEVSIVFIPCGHLVVCKDCAPSLRKCPICRGTIKGTVRTFLS
NP_001074194 Can  CKVCMDKEVSIVFIPCGHLVVCRDCAPSLRKCPICRGTVRGTVRTFLS
XP_002708629 Ory  CKVCMDKEVSIVFIPCGHLVVCKDCAPSLRKCPICRGTIKGTVRTFLS
XP_002666015 Dan  CKVCMDKEVNIVFIPCGHLVVCKECAPSLRKCPICRGMVKGTVRTFLS
NP_001007823 Gal  CKVCMDKEVSIVFIPCGHLVVCKECAPSLRKCPICRGTIKGTVRTFLS
NP_001005449 Xen  CKKCMDQEVSIVFIPCGHLVVCKDCAPSLRKCPICRGTIKGTVRTFLS
NP_477127 Dro     CKVCLDEEVGVVFLPCGHLATCNQCAPSVANCPMCRADIKGFVRTFLS
XP_001361948 Drp  CKVCLDEEVGVVFLPCGHLATCNQCAPSVANCPLCRADIKGFVRTFLS
XP_975027 Tri     CKICMDAEVGIVFLPCGHLTTCVNCAPNLEDCPLCRSAIKATVRTFLS
XP_396819 Api     CKICMDREIAIVFLPCGHLATCVYCAPSLTYCLMCRQEIKAIVRTFLS
XP_001606042 Nas  CKICMDREVAVVFLPCGHLSTCVFCAPSLTHCPMCRQDIRATVRTFLA
```

Figure S4. Sequence alignment of orthologous Diap2. All the orthologous Iap2/Diap2 from arthropods possess a tandem repeat of 3 BIR domains and 1 RING domain. Apart from a tandem repeat of 3 BIR domains and 1 RING domain, all orthologous Iap2/Diap2 from vertebrates acquired an additional CARD domain. Iap2/Diap2 from arthropods lack the corresponding CARD domain, which were indicated by broken line rectangle. Hom, *Homo sapiens*; Pan, *Pan troglodytes*; Mac, *Macaca mulatta*; Pon, *Pongo abelii*; Mus, *Mus musculus*; Rat, *Rattus norvegicus*; Orn, *Ornithorhynchus anatinus*; Mon, *Monodelphis domestica*; Bos, *Bos taurus*; Equ, *Equus caballus*; Can, *Canis lupus familiaris*; Ory, *Oryctolagus cuniculus*; Dan, *Danio rerio*; Gal, *Gallus gallus*; Xen, *Xenopus (Silurana) tropicalis*; Dro, *Drosophila melanogaster*; Drp, *Drosophila pseudoobscura*; Tri, *Tribolium castaneum*; Api, *Apis mellifera*; Nas, *Nasonia vitripennis*.
