# Supplementary material for: Comprehensively Surveying Structure and Function of RING Domains from Drosophila melanogaster
Source: PLoS One. 2011 Sep 2;6(9):e23863. doi: 10.1371/journal.pone.0023863 (PMC3166285; doi:10.1371/journal.pone.0023863)
Supplement: Figure S5 — Analysis of main-chain conformation of the solved RING domains. (A) Superimposition of all the solved RING/non-U-box domains by sequence alignments using c-Alpha in each residue. Four regions (N-loop, the first β-sheet region, βα-region and C-loop) with reliable RMSD (average≤2.0 Å), lacking insertions and deletions were detected. (B) N-terminal loop superposed by 9 residues. (C) The first β-sheet region superposed by 7 residues. (D) βα-region superposed by 13 residues. (E) C-terminal loop superposed by 6 residues. (F) Superimposition of all the solved RING/U-box domains by sequence alingments. (G) Superimposition of 1FBVA (RING/non-U-box type) and 2OXQ (RING/U-box type) domains by sequence alingments. 2OXQ (RING/U-box type) are provided with structural extension at C-terminal (1FBVA: schematic style colored by yellow; 2OXQ: solid ribbon style colored by secondary structure elements). N: amino terminal; C: carboxyl terminal; β-sheet: antiparallel β-strands. The backbones of RING domains were superposed by C-Alpha atom in each residue. (PDF) [file pone.0023863.s005.pdf]

Ying et al. Figure S5

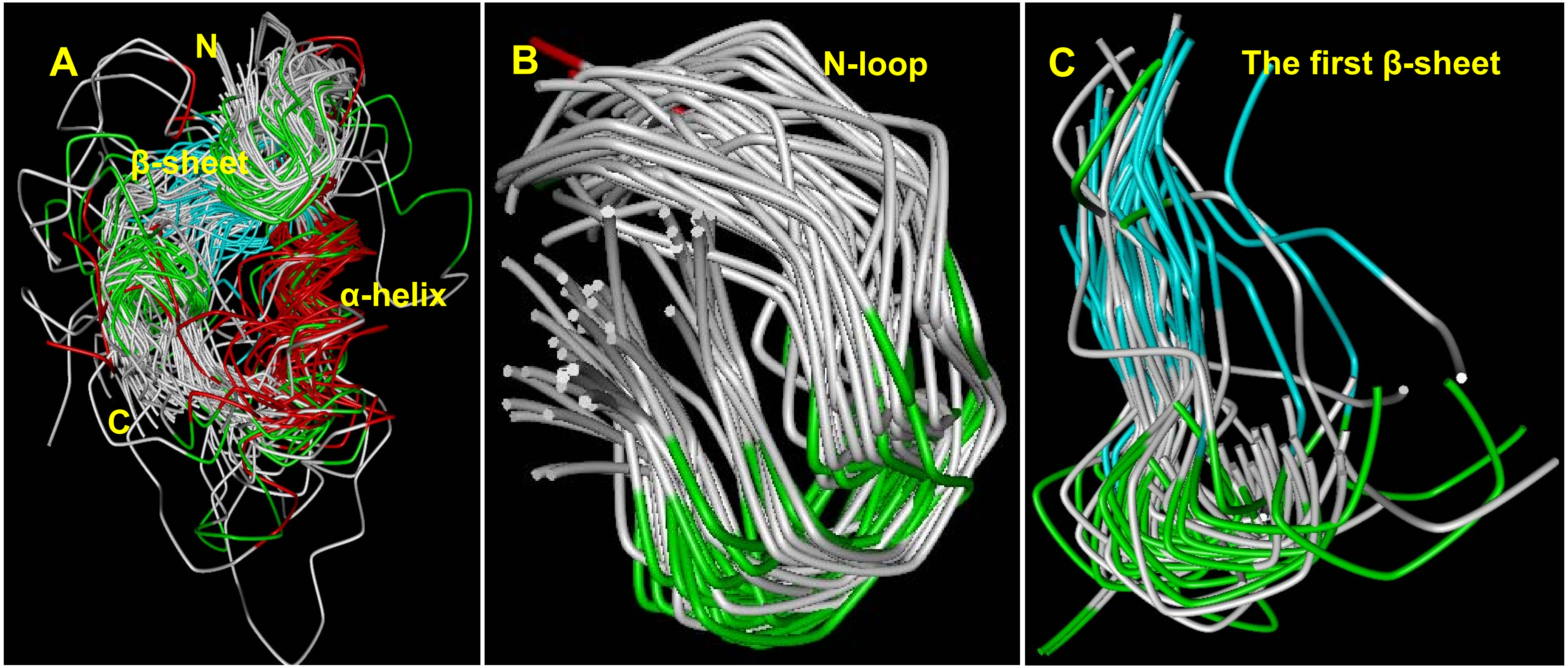

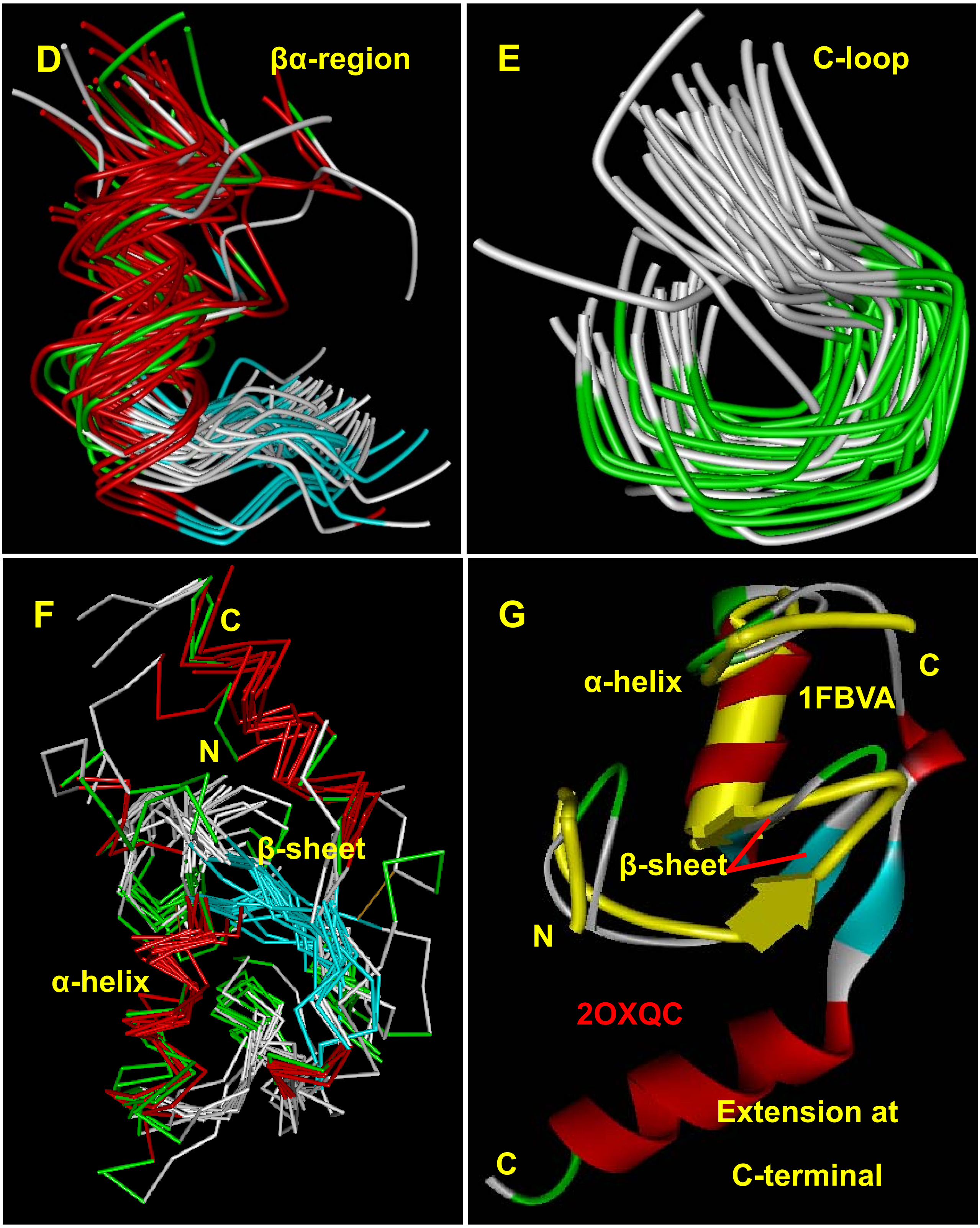

Figure S5. Analysis of main-chain conformation of the solved RING domains. (A) Superimposition of all the solved RING/non-U-box domains by sequence alignments using c-Alpha in each residue. Four regions (N-loop, the first β-sheet region, βα-region and C-loop) with reliable RMSD (average≤2.0 Å), lacking insertions and deletions were detected. **(B)** N-terminal loop superposed by 9 residues.

(C) The first β-sheet region superposed by 7 residues. **(D)** βα-region superposed by 13 residues. **(E)** C-terminal loop superposed by 6 residues. **(F)** Superimposition of all the solved RING/U-box domains by sequence alingments. **(G)** Superimposition of 1FBVA (RING/non-U-box type) and 2OXQ (RING/U-box type) domains by sequence alingments. 2OXQ (RING/U-box type) are provided with structural extension at C-terminal (1FBVA: schematic style colored by yellow; 2OXQ: solid ribbon style colored by secondary structure elements). N: amino terminal; C: carboxyl terminal; β-sheet: antiparallel β-strands. The backbones of RING domains were superposed by C-Alpha atom in each residue.
