# Supplementary material for: Comprehensively Surveying Structure and Function of RING Domains from Drosophila melanogaster
Source: PLoS One. 2011 Sep 2;6(9):e23863. doi: 10.1371/journal.pone.0023863 (PMC3166285; doi:10.1371/journal.pone.0023863)
Supplement: Figure S6 — Cartoons of VDW surfaces created for all hydrophobic residues of RING domains. Based on the van der Waals radius (VDW) of each atom in the molecule, VDW surface were created for all hydrophobic residues of RING domains, which were colored by Electrostatic potential (It calculates Gasteiger charges for the atoms that comprise the surface and maps the electrostatic potentials representing the charges to the surface). The spatial distribution of consensus and conservation of residues in RING domains were respectively indicated by blue (hydrophobic residues) and yellow letters (polar residues). 3D structures of RING domains were displayed by atom of ball and stick, which were colored by elements. (PDF) [file pone.0023863.s006.pdf]

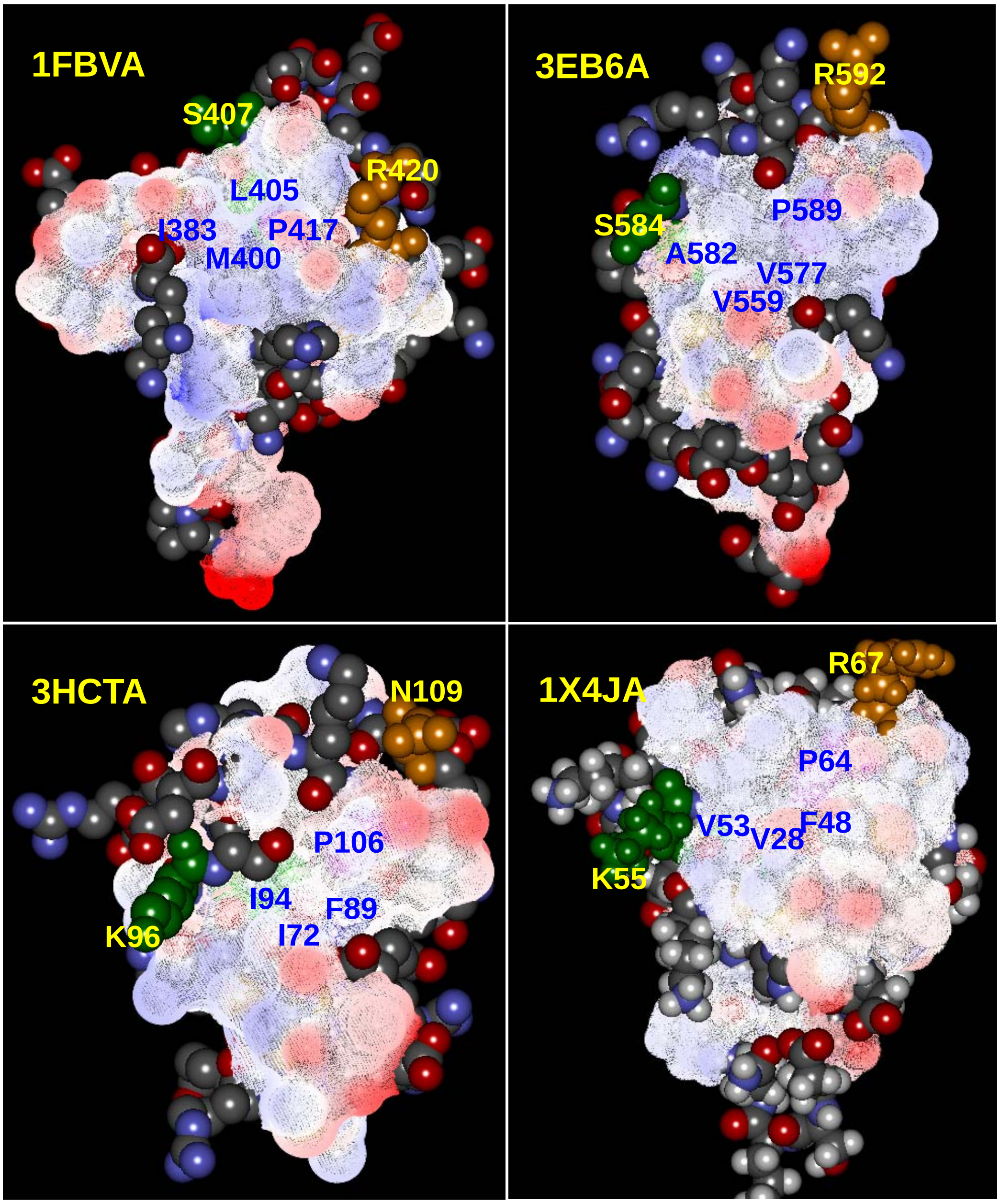
1FBVA
S407
R420
L405
I383
P417
M400
3EB6A
R592
P589
S584
A582
V577
V559
3HCTA
N109
P106
K96
I94
F89
I72
1X4JA
R67
P64
V53
V28
F48
K55

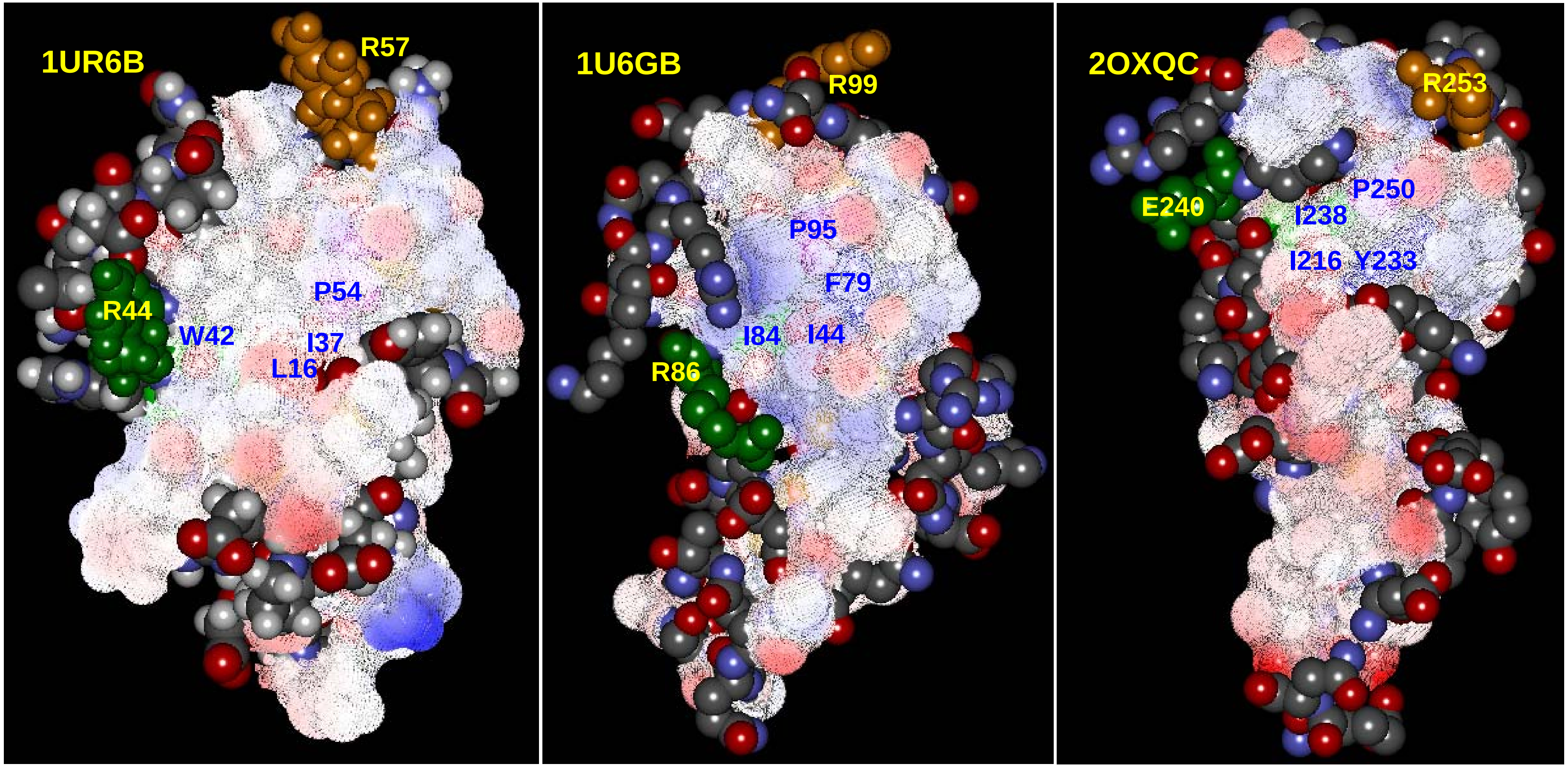

Figure S6. Cartoons of VDW surfaces created for all hydrophobic residues of RING domains. Based on the van der Waals radius (VDW) of each atom in the molecule, VDW surface were created for all hydrophobic residues of RING domains, which were colored by Electrostatic potential (It calculates Gasteiger charges for the atoms that comprise the surface and maps the electrostatic potentials representing the charges

to the surface). The spatial distribution of consensus and conservation of residues in RING domains were respectively indicated by blue (hydrophobic residues) and yellow letters (polar residues). 3D structures of RING domains were displayed by atom of ball and stick, which were colored by elements.
