# Supplementary material for: Comprehensively Surveying Structure and Function of RING Domains from Drosophila melanogaster
Source: PLoS One. 2011 Sep 2;6(9):e23863. doi: 10.1371/journal.pone.0023863 (PMC3166285; doi:10.1371/journal.pone.0023863)
Supplement: Figure S7 — Cartoons of 3D complexes of RING-E3 and E2 pairs. A: Cbl-UbcD10; B: Iap2-Eff; C: Mura-Ben; D: Traf6-Eff; E: Cont4-Eff; F: Chip-Eff; G: Chip-Ben. A-I to G-I: Targets and templates were respectively represented by the purple ribbon models and the light blue schematic models. Structural similarity between them was shown by superimposition of their structures (N, amino terminal; C, carboxyl terminal). Cantact interfaces of RING-E3 and E2 complexes were illuminated by purple dot circles. I and II represent the first and the second Zn-binding sites. The 2 sites are respectively located on 2 sides of RING-E3 domain, by which form a characteristic “cross-brace” zinc-binding topology of RING domains. A-II to G-II: Close-up view of interaction residues in the intermolecular interfaces of their 3D complexes. The side chains of E2 and RING-E3 3D complexes involved in E2/E3 interactions were presented by solid ribbon. Resides that make significant directly contacts observed in the modeling complexes were presented by stick model, and were numbered by precursor peptides. The numbers for all resides in the figure correspond to those in the text and the tables. The interaction residues in the interface were respectively indicated by yellow (E2s) and white (RING-E3s) letters. The conserved hydrophobic contacts of intermolecular interfaces observed in the modeling complexes were highlighted by red dot circles. Hydrogen bonds of intermolecular interfaces of complexes formed by carbonyl-group oxygen and amino-group hydrogen were showed by white dot lines. (PDF) [file pone.0023863.s007.pdf]

A-I (C3HC4-type)

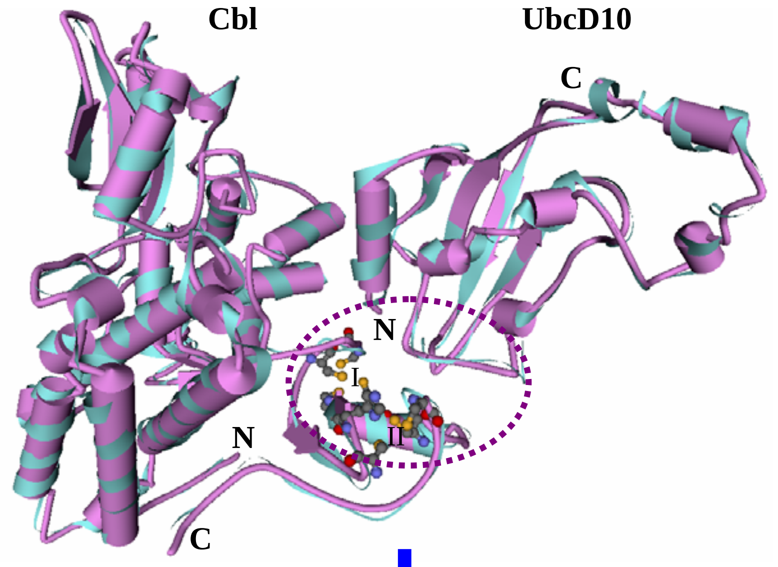

A-II

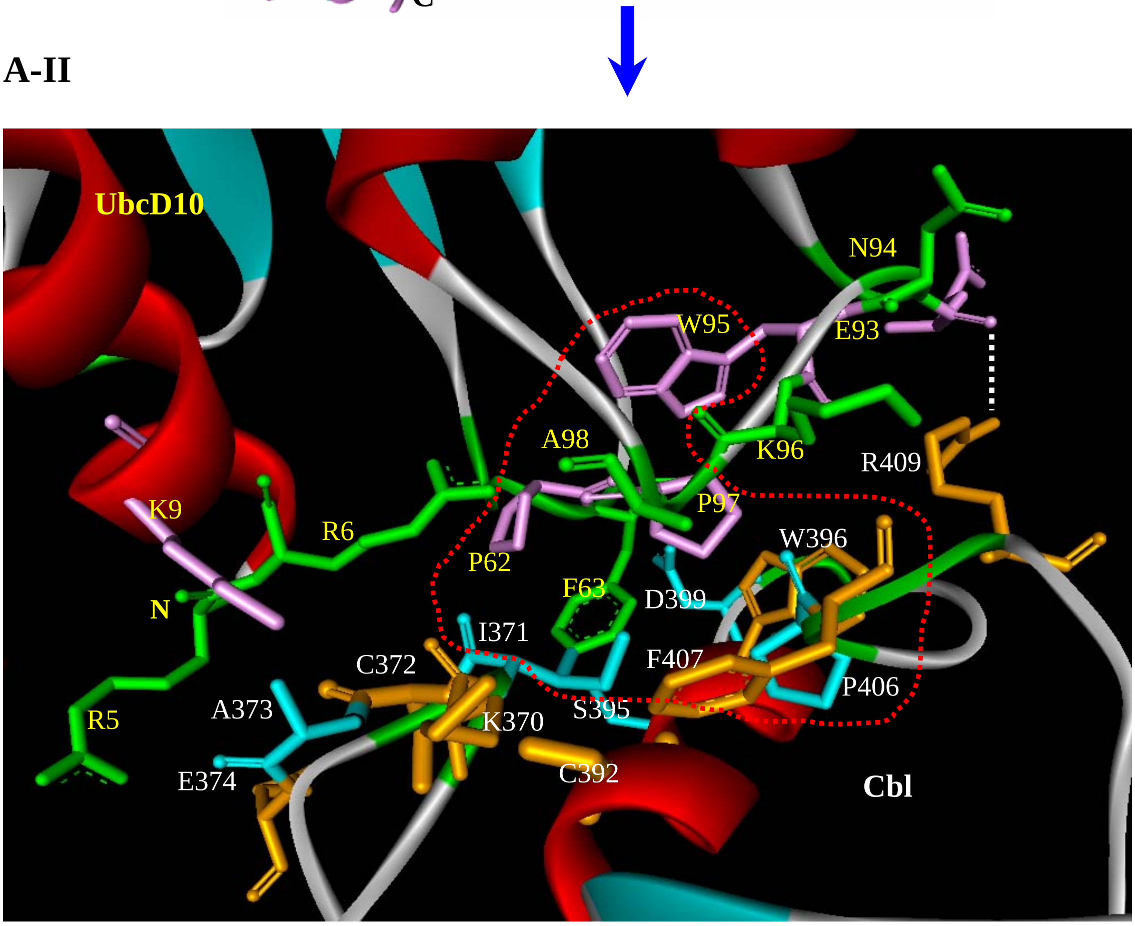

B-I (C3HC4-type)

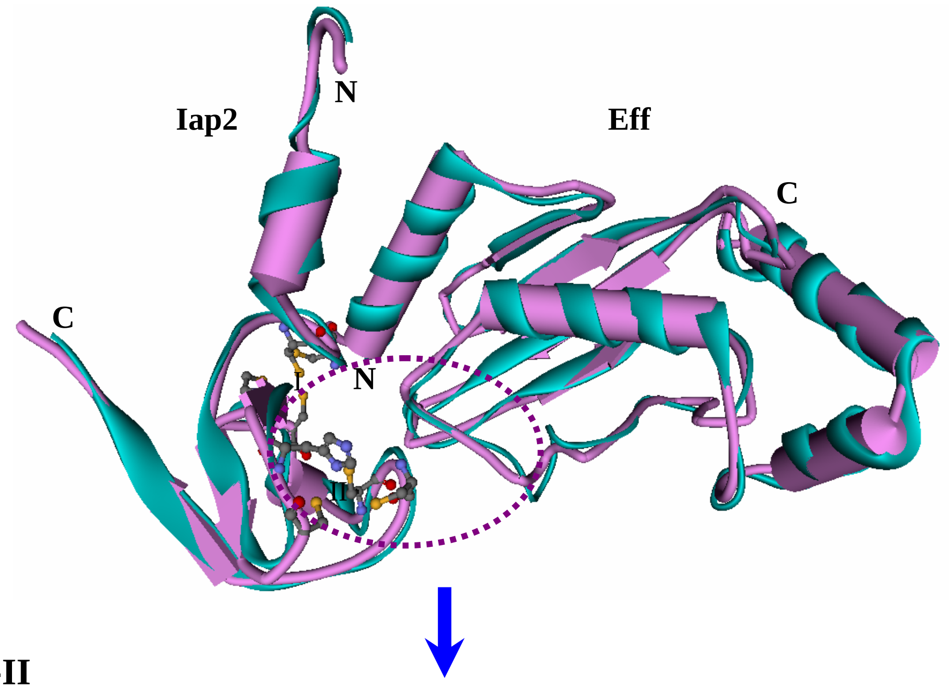

B-II

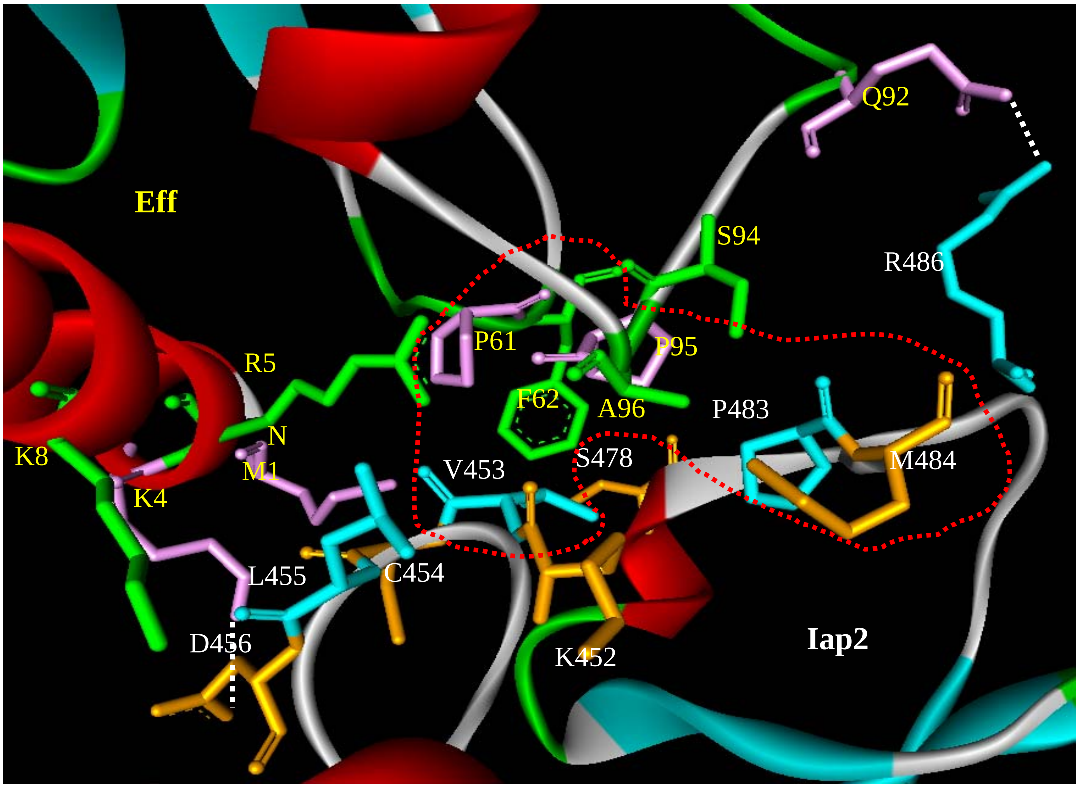

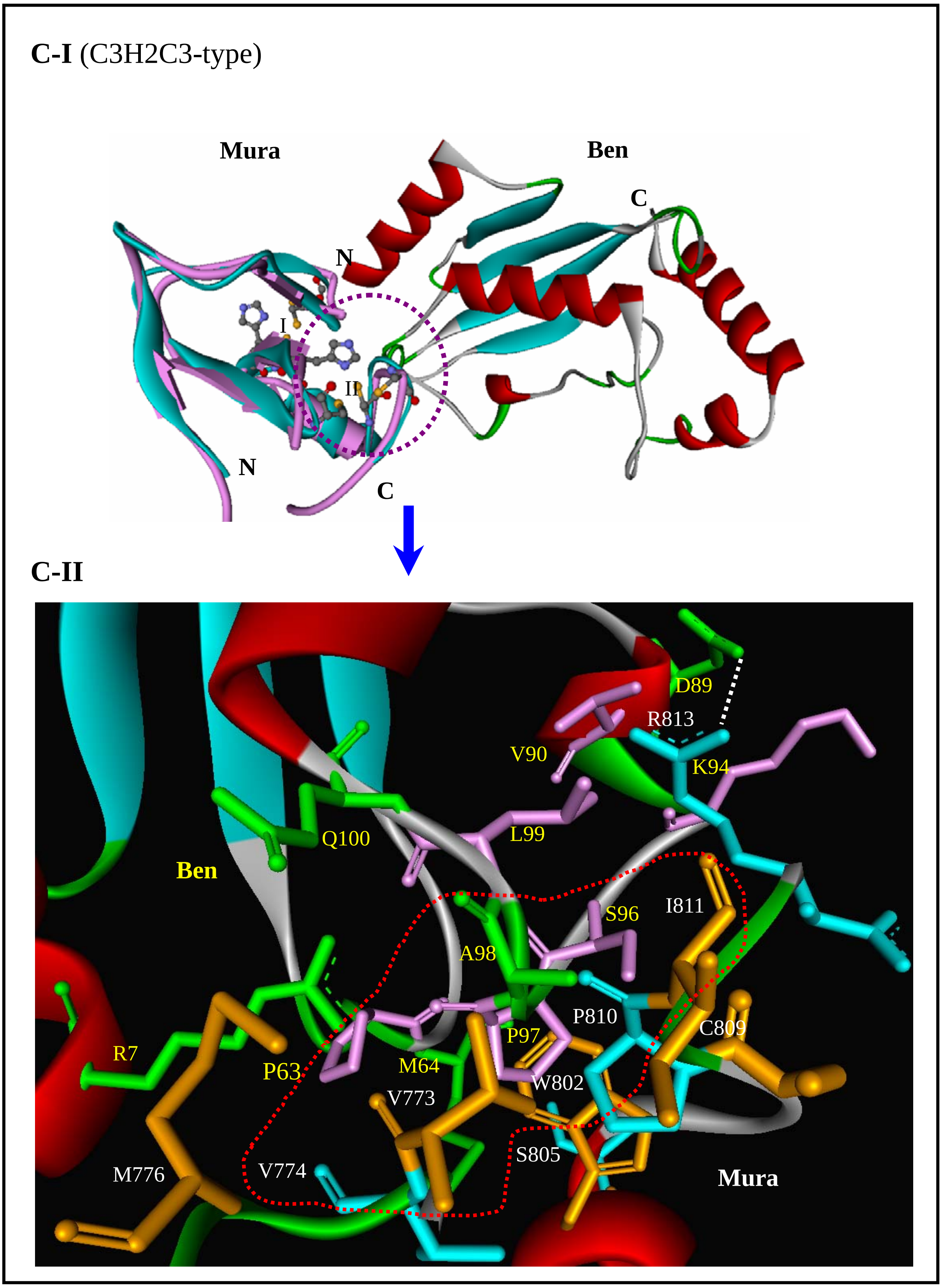

C-I (C3H2C3-type)
Mura
Ben
C
N
I
II
N
C
C-II
D89
R813
V90
K94
Q100
L99
Ben
S96
I811
A98
P810
C809
R7
P63
M64
P97
W802
V773
S805
M776
V774
Mura

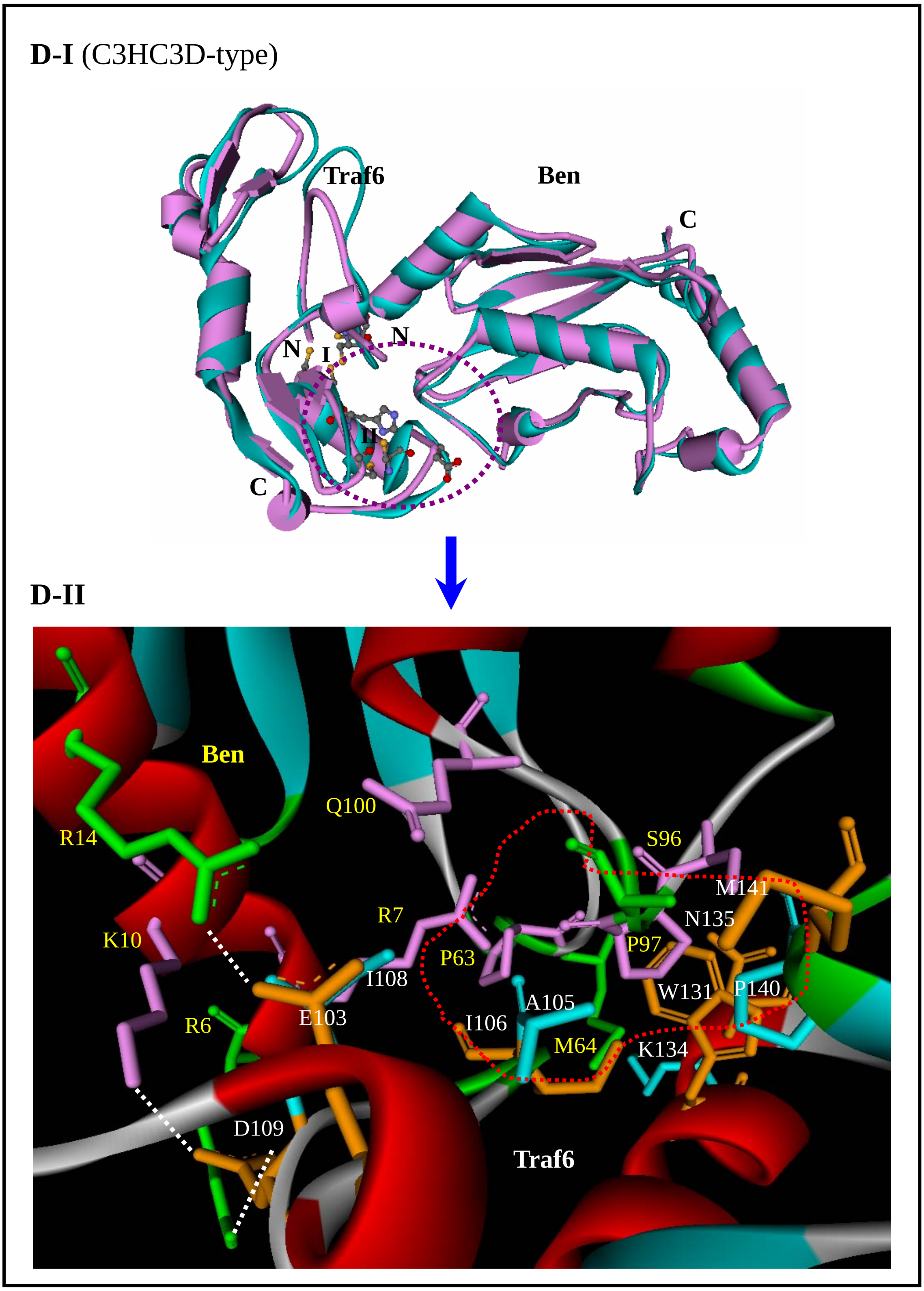
D-I (C3HC3D-type)
Traf6
Ben
C
N
I
N
II
C
D-II
Ben
Q100
R14
S96
M141
R7
N135
K10
P63
P97
I108
A105
W131
P140
R6
E103
I106
M64
K134
D109
Traf6

E-I (C4C4-type)

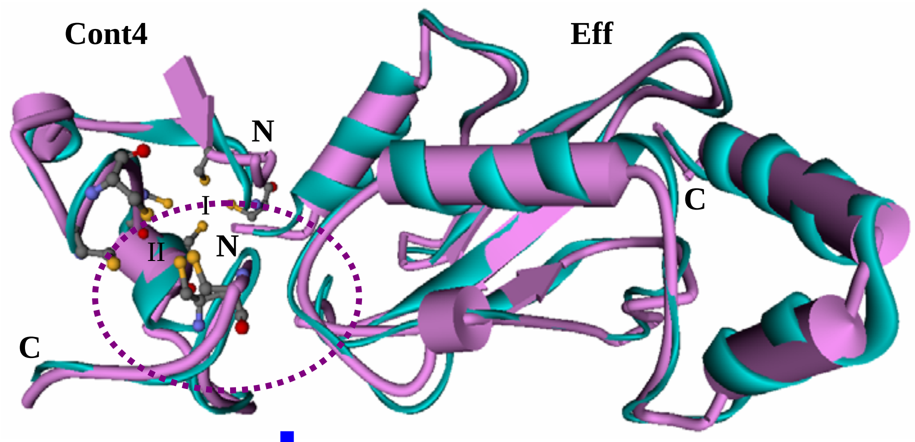

E-II

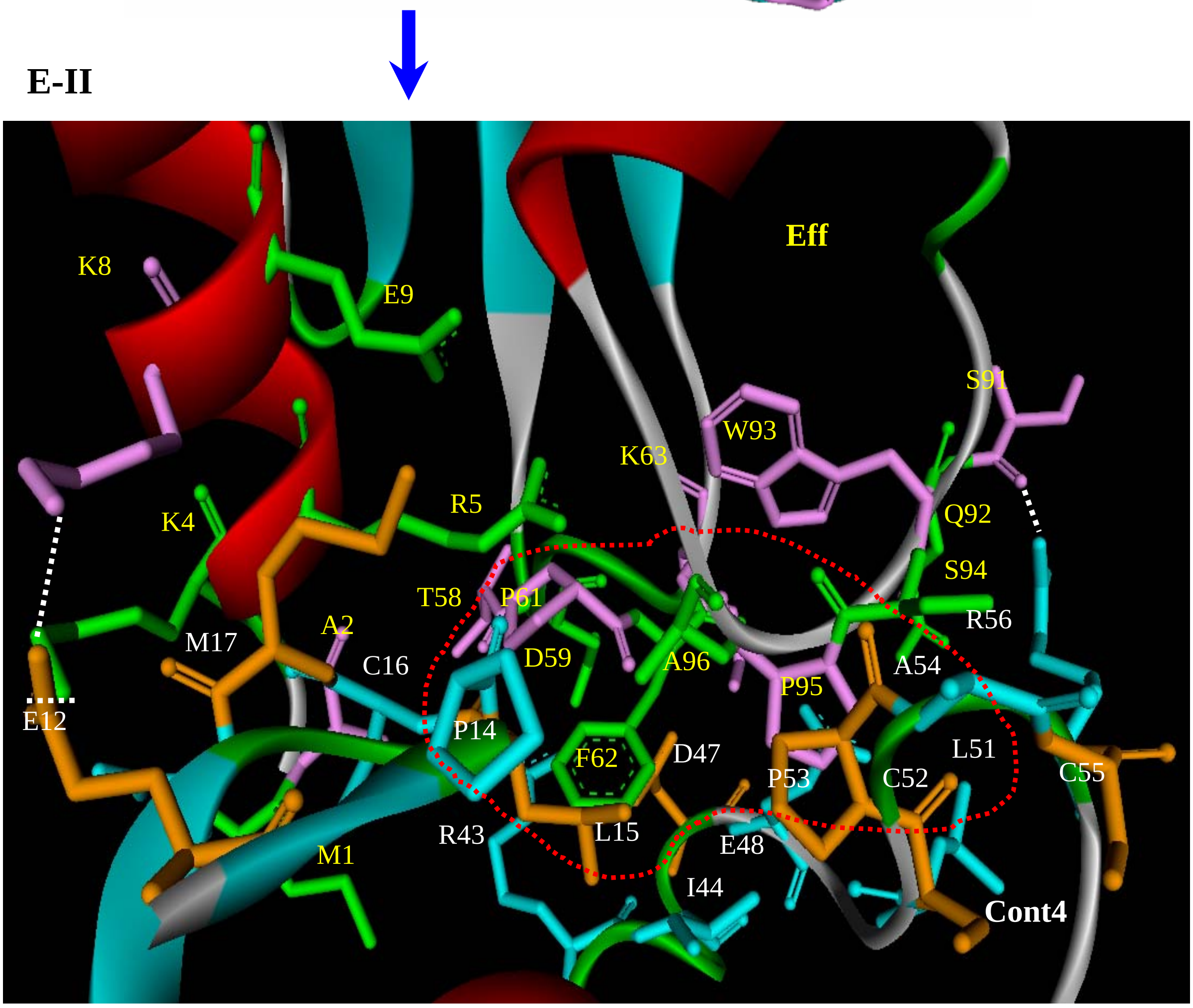

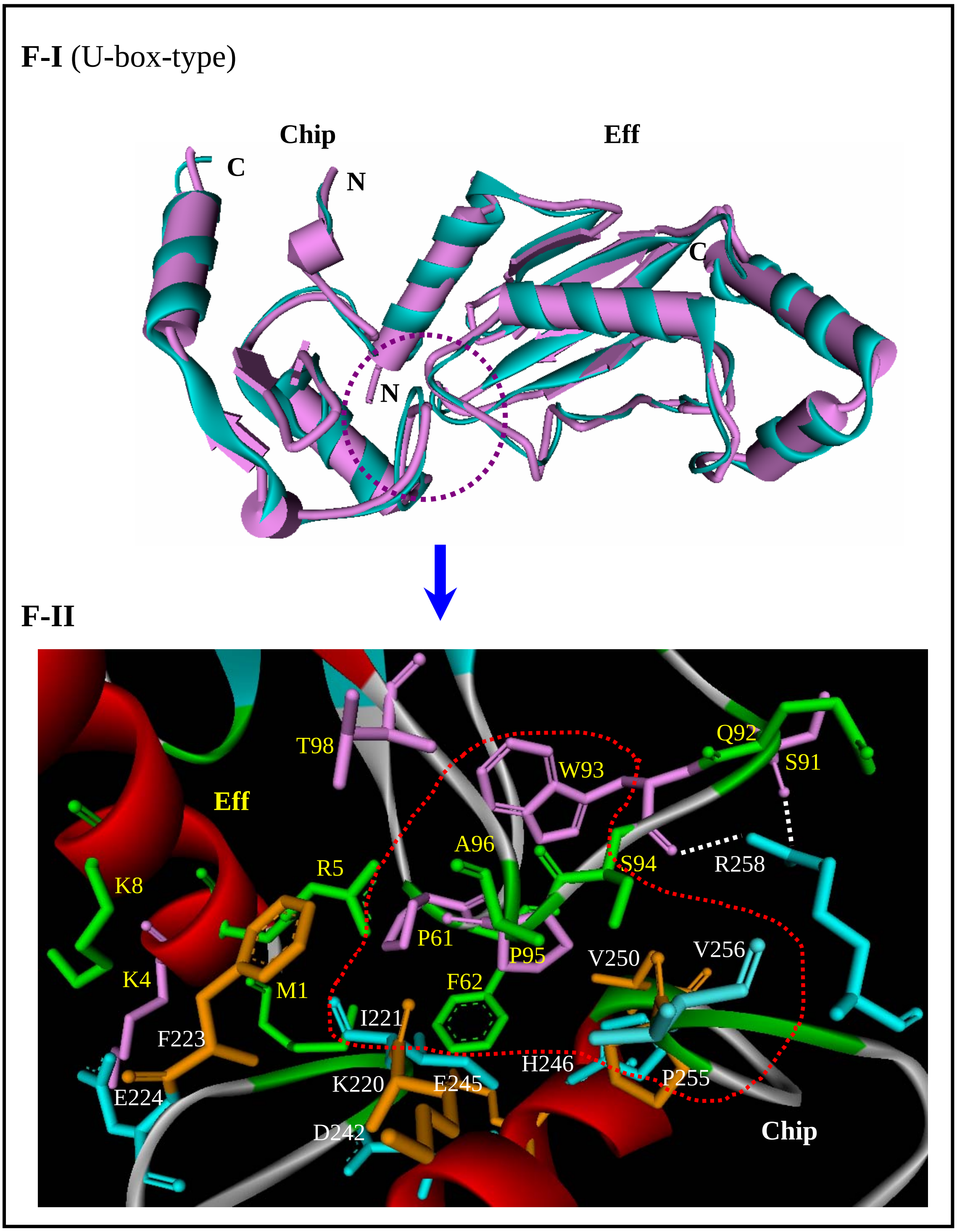
F-I (U-box-type)
Chip
Eff
C
N
N
C
F-II
Eff
T98
W93
Q92
S91
A96
S94
R258
R5
K8
P61
P95
V250
V256
K4
M1
F62
I221
F223
H246
K220
E245
P255
E224
D242
Chip

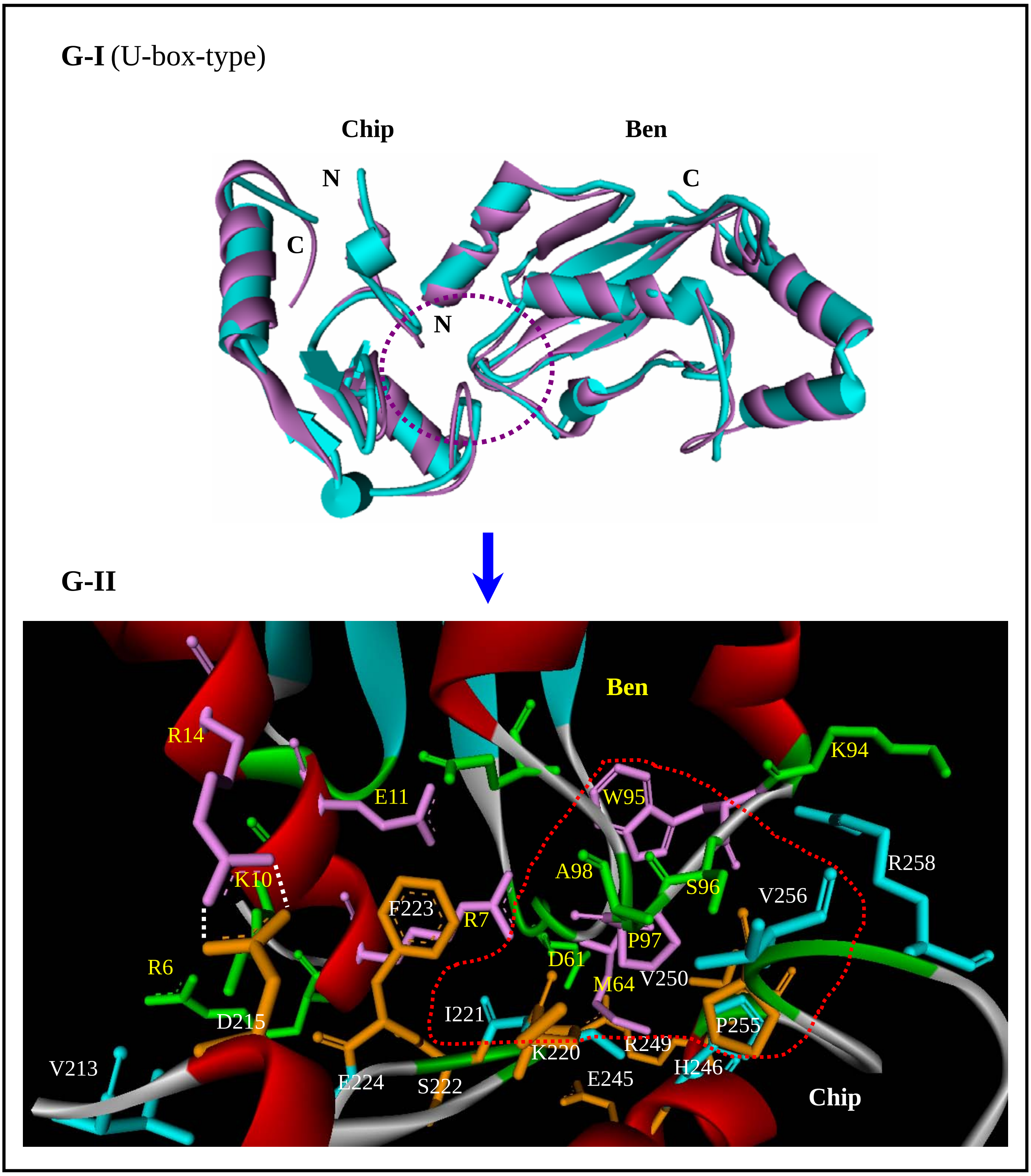

Figure S7. Cartoons of 3Ds complexes of RING-E3 and E2 pairs. A: Cbl-UbcD10; **B**: Iap2-Eff; **C:** Mura-Ben; **D:** Traf6-Eff; **E:** Cont4-Eff; **F:** Chip-Eff; **G:** Chip-Ben. **A-I to G-I:** Targets and templates were respectively represented by the purple ribbon models and the light blue schematic models. Structural similarity between them was shown by superimposition of their structures (N, amino terminal; C, carboxyl terminal). Cantact interfaces of RING-E3 and E2 complexes were illuminated by

purple dot circles. I and II represent the first and the second Zn-binding sites. The two sites are respectively located on two sides of RING-E3 domain, by which form a characteristic “cross-brace” Zinc-binding topology of RING domains. **A-II to G-II:** Close-up view of interaction residues in the intermolecular interfaces of their 3Ds complexes. The side chains of E2 and RING-E3 3Ds complexes involved in E2/E3 interaction were presented by solid ribbon. Resides that make significant directly contacts observed in the modeling complexes were presented by stick model, and were numbered by precursor peptides. The numbers for all resides in the figure correspond to those in the text and the tables. The interaction residues in the interface were respectively indicated by yellow (E2s) and white (RING-E3s) letters. The Conserved hydrophobic contacts of intermolecular interfaces observed in the modeling complexes were highlighted by red dot circles. Hydrogen bonds of intermolecular interfaces of complexes formed by carbonyl-group oxygen and amino-group hydrogen were showed by white dot lines.
