# Supplementary material for: Comprehensively Surveying Structure and Function of RING Domains from Drosophila melanogaster
Source: PLoS One. 2011 Sep 2;6(9):e23863. doi: 10.1371/journal.pone.0023863 (PMC3166285; doi:10.1371/journal.pone.0023863)
Supplement: Figure S8 — Close-up view of interaction residues in the intermolecular interfaces of 3D complexes of RING-E3 and E2 pairs. A: Cbl-UbcD10; B: Iap2-Eff; C: Mura-Ben; D: Traf6-Eff; E: Cont4-Eff; F: Chip-Eff; G: Chip-Ben. The side chains of 3D complexes of RING-E3 and E2 pairs involved in their interactions were presented by solid ribbon. Resides that make significant directly contacts observed in the modeling complexes were presented by stick model, and were numbered by precursor peptides. The numbers for all resides in the figure correspond to those in the text and the tables. The interaction residues in the intermolecular interface were respectively indicated by yellow (E2s) and white (RING-E3s) letters. The conserved hydrophobic contacts of intermolecular interfaces observed in the modeling complexes were highlighted by red dot circles. Hydrogen bonds of intermolecular interfaces formed by carbonyl-group oxygen and amino-group hydrogen were showed by white dot lines. (PDF) [file pone.0023863.s008.pdf]

Ying et al. Figure S8

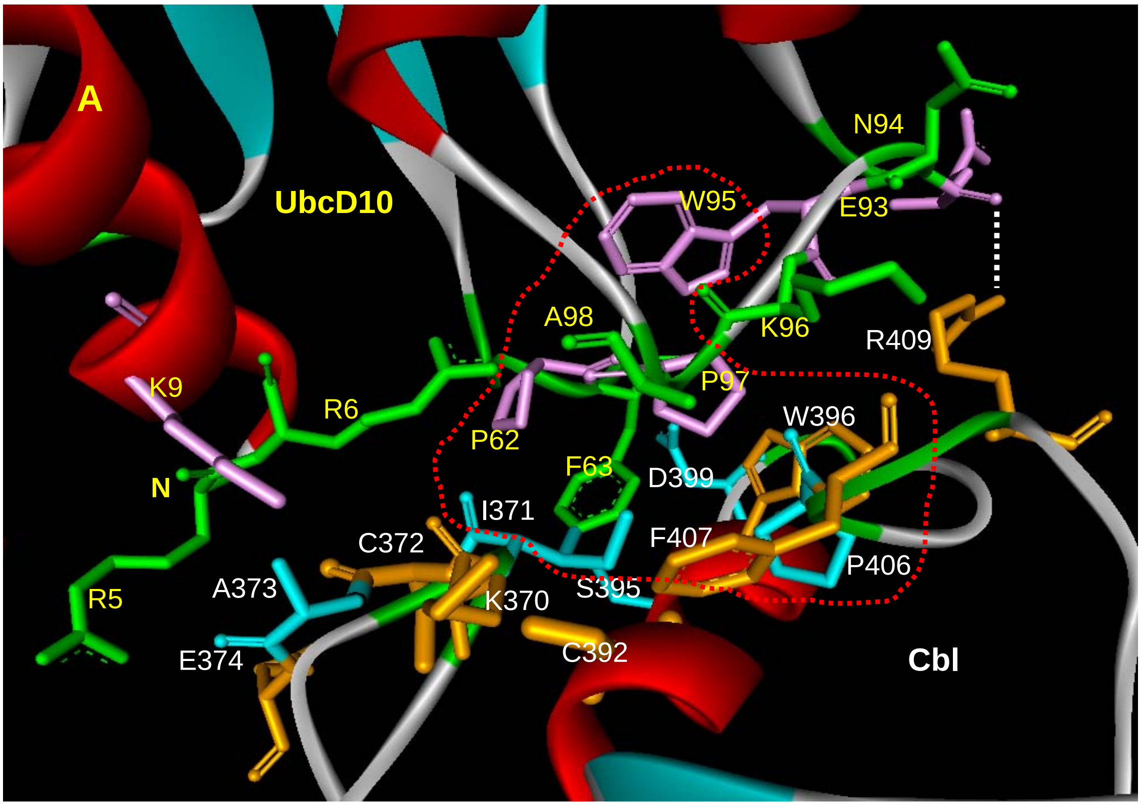

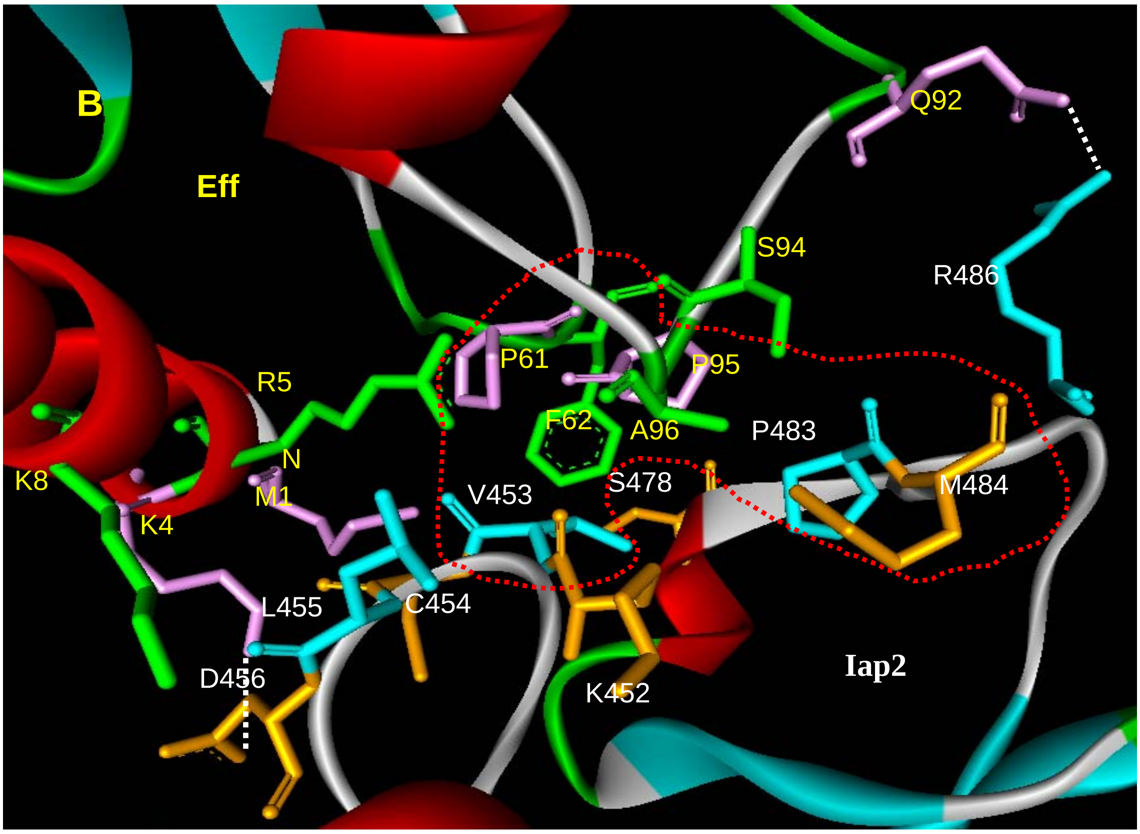

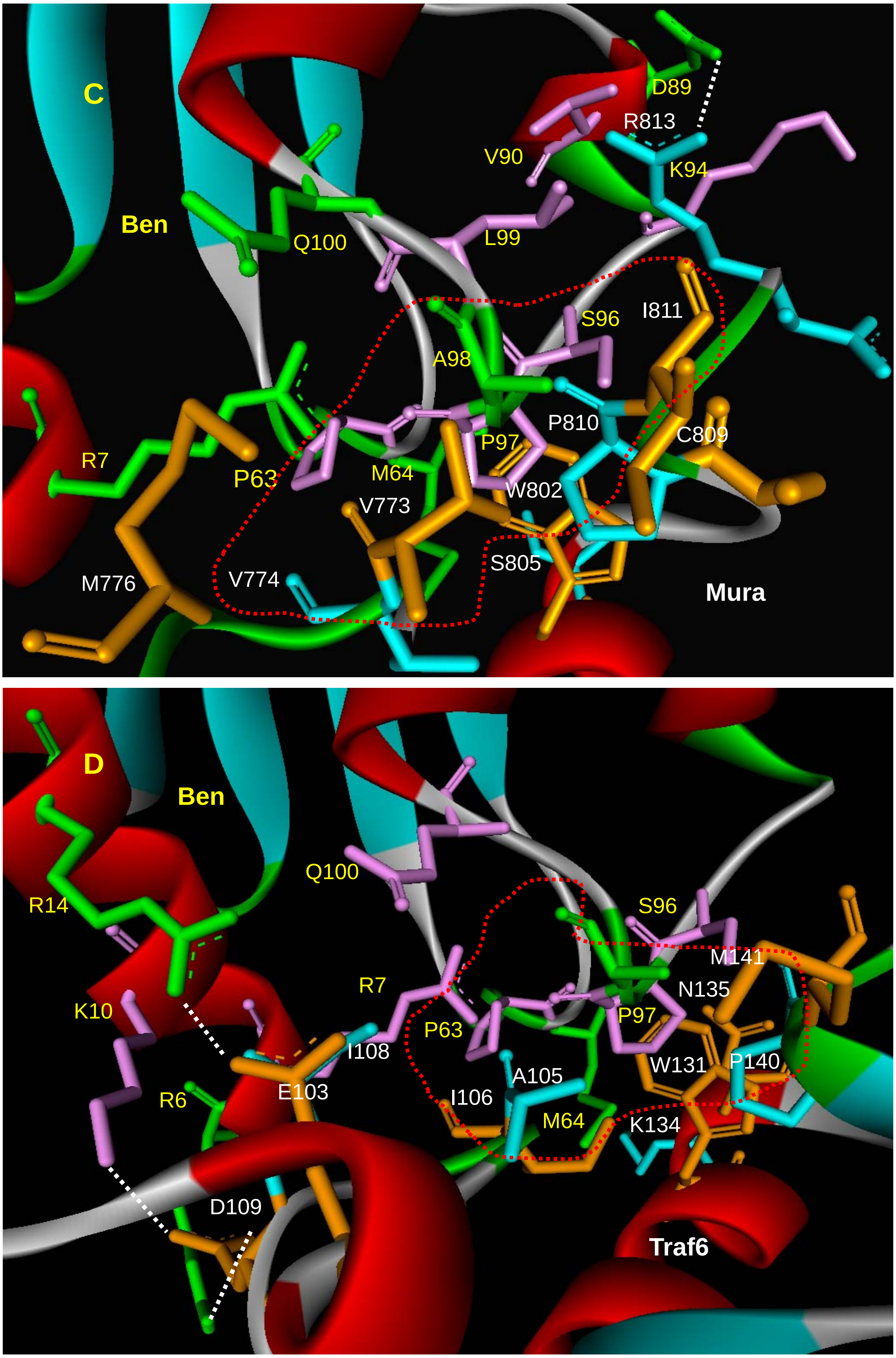
C
Ben
Q100
V90
D89
R813
K94
L99
S96
I811
A98
P810
C809
R7
P63
M64
P97
W802
V773
S805
M776
V774
Mura
D
Ben
Q100
R14
S96
M141
N135
R7
K10
P63
P97
I108
W131
P140
A105
E103
R6
I106
M64
K134
D109
Traf6

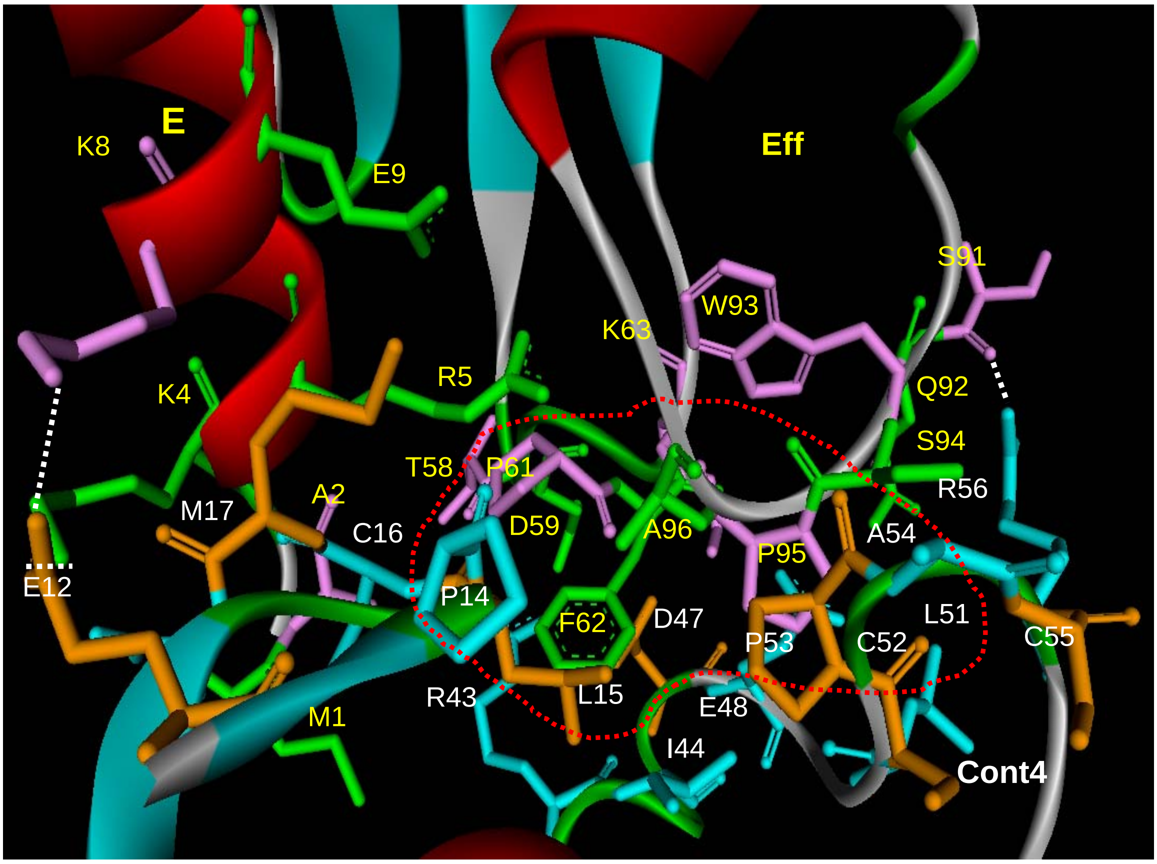
E
K8
E9
Eff
S91
W93
K63
Q92
R5
K4
S94
R56
T58
P61
A2
M17
C16
D59
A96
A54
P95
E12
P14
L51
F62
D47
C55
P53
C52
R43
L15
E48
M1
I44
Cont4

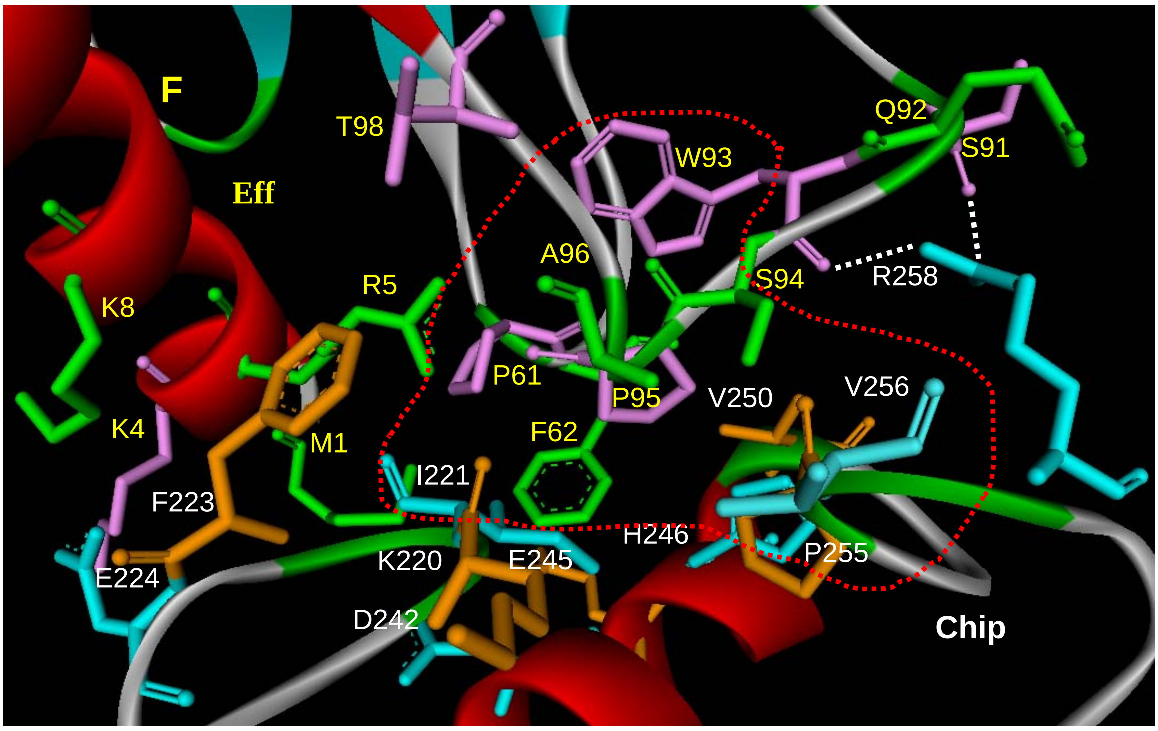
F
T98
Q92
W93
S91
Eff
A96
R258
S94
R5
K8
V256
P61
P95
V250
K4
M1
F62
I221
F223
H246
P255
K220
E245
E224
D242
Chip

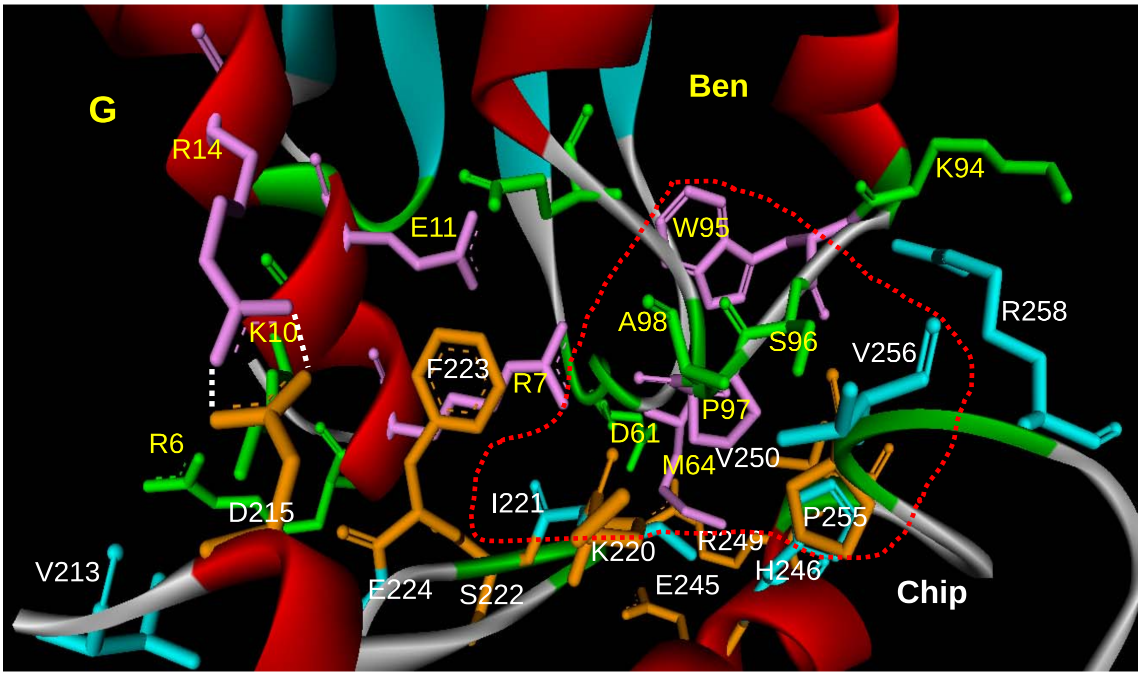

Figure S8. Close-up view of interaction residues in the intermolecular interfaces of 3D complexes of RING-E3 and E2 pairs. A: Cbl-UbcD10; **B:** Iap2-Eff; **C**: Mura-Ben; **D:** Traf6-Eff; **E:** Cont4-Eff; **F:** Chip-Eff; **G:** Chip-Ben. The side chains of 3D complexes of RING-E3 and E2 pairs involved in their interactions were presented by solid ribbon. Resides that make significant directly contacts observed in the modeling complexes were presented by stick model, and were numbered by precursor peptides. The numbers for all resides in the figure correspond to those in the text and the tables. The interaction residues in the intermolecular interface were respectively indicated by yellow (E2s) and white (RING-E3s) letters. The conserved hydrophobic contacts of intermolecular interfaces observed in the modeling complexes were highlighted by red dot circles. Hydrogen bonds of intermolecular interfaces formed by carbonyl-group oxygen and amino-group hydrogen were showed by white dot lines.
