# Supplementary material for: Comprehensively Surveying Structure and Function of RING Domains from Drosophila melanogaster
Source: PLoS One. 2011 Sep 2;6(9):e23863. doi: 10.1371/journal.pone.0023863 (PMC3166285; doi:10.1371/journal.pone.0023863)
Supplement: Table S6 — Hydrophobic residues identified close to the central hydrophobic residues within 4 Å. (DOC) [file pone.0023863.s014.doc]

**Table S6** Hydrophobic residues identified close to the central hydrophobic residue within 4Å

| **RING type** | **PDB ID** | **Residues** | **Reference** |
| --- | --- | --- | --- |
| **C3HC4** | 1FBVA | I 383; V391 I393; *M400*; L405 W408; P417 F418 | [1] |
|  | 3HCTA | P71 I72 L77; A80 V81; *F89*; I94 I95; P106 V107 | [2] |
|  | 1RMDA | I28 L33; P36 V37; L44 *F45*; I50; P62 | [3] |
|  | 2CKLB | P52 I53 L58; M62; *F71*; I76; P88; | [4] |
|  | 2CKLA | V19 L20 F25; A28 I31; *F38*; I43; P54 I55 | [4] |
|  | 2ECJA | V20; P28 V29 I31; *F37*; I42; P56 V57 |  |
|  | 2ECWA | P23 I24 L29; P32 V33 A35; *F41*; I46; P63 V64 |  |
|  | 1JM7A | P25 I26 I31; P34 V35 I42; *F43*; M48 L51; P62 L63 | [5] |
|  | 1JM7B | L57; P60 V61 L63; I69 *F70*; V75; P84 V85 | [5] |
|  | 2ECNA | I20; A26 L28 L30; *F36*; I4; P52 I53 |  |
|  | 2CSYA | F19 I20 F25; P28 V29; *F37*; A42; Y53 I54 |  |
|  | 1CHCA | P9 I10; M20 A21; A27 *F28*; L33; P44 L45 | [6] |
|  | 1Z6UA | V740; P743 V744; *V752*; L757; P769 |  |
|  | 2CT2A | P19 I20 F25; P32 L35; *I41*; L46; P60 F61 |  |
|  | 3FL2A | V731; P734 I735; *V743*; L748 |  |
|  | 2EGPA | I17 L22; P25 L26 L28; *L34*; I39; P57 V58 |  |
|  | 2ECVA | P23 I24 L29; P32 L33 L35; *F41*; L46; P63 V64 |  |
|  | 2EA6A | P19 I20; I35 V36; V43 *F44*; L49; P60 |  |
|  | 1BORA | F6; A16; L25; *L28*; L33; M38; P41; | [7] |
|  | 2YSLA | P24 I25 L30; P33 V34 I36; *F42*; I47; F57 P61 L62 |  |
|  | 2D8TA | A19 I20; P28 V29 L31; V36 *F37*; V42; A53 L54 |  |
|  | 3KNVA | A36 L41; P44 F45; *Y53*; L58 I61; L85 | [8] |
|  | 2DJBA | I20 L25; I31; *F38*; I43; P54 |  |
|  | 1G25A | P7; L21 M22 V23; *L30*; V35 L38 F39; P47 F58 | [9] |
|  | 2ECYA | L25 P28; *F37*; M42 L45; A55 |  |
|  | 2ECGA | I30 I36; F40; L46 *V47*; P60 M61 |  |
|  | 3EB6A | V559 V565; I567 F569; V576 *V577*; | [10] |
|  | 2EA5A | V20 V26; W28 L30; *L38*; V43 F46; P50 |  |
|  | 3I2D | P362 M368; P371; *F383*; A385 F388; P401 L412 | [11] |
| **C3H2C3** | 2EP4A | A79 V80 F85; L91; A99 *F100*; L105; P116 L117 |  |
|  | 2ECTA | P19 V20; V31 L34; L39 *F40*; I45; P56V57 |  |
|  | 1X4JA | V27 V28 F33; L39 V41 L42; *F48*; V53; P64 I65 |  |
|  | 2ECMA | P9 I10 I15; A22 L25; L30 *L31*; M39; P47 |  |
|  | 2JRJA | I19 I24; A31 L34; L39 *L40*; M48; L57 | [12] |
|  | 1IYMA | A135 V136 L141; A147 F149 L150; *F157*; V162; P173 L174 | [13] |
|  | 1V87A | I29 I30 L35; V55 L58; A64 *F65*; L67 L70; P86 |  |
| **C2H2C4** | 2VJEA | V439; I450; L458 *M459* A460; P476 V477 | [14] |
|  | **2VJEB** | Nothing | [14] |
| **C4HC3** | 2D8SA | I20; P30 L31 I32 P34; L41 F43 *V44*; L49; |  |
|  | 1VYXA | W10 I11; L16; F21 A23; L30 V33; L38; | [15] |
|  | 2CT0A | I20 L25; I36 *M38*; V43; P56 W62 |  |
| **C4C4** | 2CSZA | A29; L35; L51; *V52*; W65; |  |
|  | 2YURA | L19 I20 M25; A28 V29 I31; *Y38*; I43; P56 |  |
|  | 1UR6B | L16 L21; F28 F29 P30; *I37*; W42 I45; P54 | [16] |
|  | 1WIMA | L12; M20 I23 I29; *F30*; L35; P54 |  |
|  | 1WEOA | I21; A37; *F43* P44 A45; P62 |  |
| **C6H3C2D** | 1U6GB | I44; V70 A71; A78 *F79* F81; I84; P95 W101 | [17] |
|  | 2ECLA | A19 I20 V25; V43 V44; *F52*; M57; P68 L69 W74 |  |
| **U-box** | 1T1HA | P249 P255 I256 M261; P264 V265;*Y273*; I278; P290 | [18] |
|  | 2F42A | I216 M221; P224; I231 *Y233*; I238; P250 V251 | [19] |
|  | 2C2VS | M241; P244 I246; I251 *Y253*; I258; P270 V271 | [20] |
|  | 2OXQC | M221 A222; P224; I231 *Y233*; I238; P250 V251 | [21] |
|  | 2YU4A | P11 I12; M17; P20 V21; *Y30*; A34 I35; P52 I61 |  |
|  | 1WGMA | P26 I27 M32; P35 V36 L38; V43 *V45*; I50; P61 F62 |  |
|  | 2BAYA | A4 I5; P13 V14 L15; I22 *F23*; L28; P39 I40 | [22] |
|  | 2QIZA | P888 M894; P897 V898 L900; M905 *I907*; I912 | [23] |
|  | 2KKYA | P120 I121 P126; I130 V132; L143 *F144*; F149; P160 L161 |  |

Note: The consensus and conservation of four hydrophobic residues in RING domains were indicated by black letters. The central hydrophobic residues were indicated by italic letters. Positions of residues in RING domains were indicated by underlines (N- and C-terminal loops: single underline; Beta-sheet: double underline; Alpha-helix: wave line). The structral data from PDB database were refered in the number of residues. 2VJEB (green letters), with Thr residue at the equivalent position of the central hydrophobic residue, is the only one of the solved RING domain who shows serious packing defects in the conserved large hydrophobic patch.

**Reference for Table S6:**

1. Zheng N, Wang P, Jeffrey PD, Pavletich NP (2000) Structure of a c-Cbl-UbcH7 complex: RING domain function in ubiquitin-protein ligases. Cell 102: 533-539.

2. Yin Q, Lin SC, Lamothe B, Lu M, Lo YC, Hura G, et al. (2009) E2 interaction and dimerization in the crystal structure of TRAF6. Nat Struct Mol Biol 16: 658-666.

3. Bellon SF, Rodgers KK, Schatz DG, Coleman JE, Steitz TA (1997) Crystal structure of the RAG1 dimerization domain reveals multiple zinc-binding motifs including a novel zinc binuclear cluster. Nat Struct Biol 4: 586-591.

4. Buchwald G, van der Stoop P, Weichenrieder O, Perrakis A, van Lohuizen M, et al. (2006) Structure and E3-ligase activity of the Ring-Ring complex of polycomb proteins Bmi1 and Ring1b. EMBO J 25: 2465-2474.

5. Brzovic PS, Rajagopal P, Hoyt DW, King MC, Klevit RE (2001) Structure of a BRCA1-BARD1 heterodimeric RING-RING complex. Nat Struct Biol 8: 833-837.

6. Barlow PN, Luisi B, Milner A, Elliott M, Everett R (1994) Structure of the C3HC4 domain by 1H-nuclear magnetic resonance spectroscopy. A new structural class of zinc-finger. J Mol Biol 237: 201-211.

7. Borden KL, Boddy MN, Lally J, O'Reilly NJ, Martin S, et al. (1995) The solution structure of the RING finger domain from the acute promyelocytic leukaemia proto-oncoprotein PML. EMBO J 14: 1532-1541.

8. Yin Q LB, Darnay BG, Wu H (2009) Structural basis for the lack of E2 interaction in the RING domain of TRAF2. Biochemistry 48: 10558-10567.

9. Gervais V, Busso D, Wasielewski E, Poterszman A, Egly JM, et al. (2001) Solution structure of the N-terminal domain of the human TFIIH MAT1 subunit: new insights into the RING finger family. J Biol Chem 276: 7457-7464.

10. Mace PD, Linke K, Feltham R, Schumacher FR, Smith CA, et al. (2008) Structures of the cIAP2 RING domain reveal conformational changes associated with ubiquitin-conjugating enzyme (E2) recruitment. J Biol Chem 283: 31633-31640.

11. Yunus AA, Lima CD (2009) Structure of the Siz/PIAS SUMO E3 ligase Siz1 and determinants required for SUMO modification of PCNA. Mol Cell 35: 669-682.

12. Sheng Y, Laister RC, Lemak A, Wu B, Tai E, et al. (2008) Molecular basis of Pirh2-mediated p53 ubiquitylation. Nat Struct Mol Biol 15: 1334-1342.

13. Katoh S, Hong C, Tsunoda Y, Murata K, Takai R, et al. (2003) High precision NMR structure and function of the RING-H2 finger domain of EL5, a rice protein whose expression is increased upon exposure to pathogen-derived oligosaccharides. J Biol Chem 278: 15341-15348.

14. Linke K, Mace PD, Smith CA, Vaux DL, Silke J, et al. (2008) Structure of the MDM2/MDMX RING domain heterodimer reveals dimerization is required for their ubiquitylation in trans. Cell Death Differ 15: 841-848.

15. Dodd RB, Allen MD, Brown SE, Sanderson CM, Duncan LM, et al. (2004) Solution structure of the Kaposi's sarcoma-associated herpesvirus K3 N-terminal domain reveals a Novel E2-binding C4HC3-type RING domain. J Biol Chem 279: 53840-53847.

16. Dominguez C, Bonvin AM, Winkler GS, van Schaik FM, Timmers HT, et al. (2004) Structural model of the UbcH5B/CNOT4 complex revealed by combining NMR, mutagenesis, and docking approaches. Structure 12: 633-644.

17. Goldenberg SJ, Cascio TC, Shumway SD, Garbutt KC, Liu J, et al. (2004) Structure of the Cand1-Cul1-Roc1 complex reveals regulatory mechanisms for the assembly of the multisubunit cullin-dependent ubiquitin ligases. Cell 119: 517-528.

18. Andersen P, Kragelund BB, Olsen AN, Larsen FH, Chua NH, et al. (2004) Structure and biochemical function of a prototypical Arabidopsis U-box domain. J Biol Chem 279: 40053-40061.

19. Xu Z, Devlin KI, Ford MG, Nix JC, Qin J, et al. (2006) Structure and interactions of the helical and U-box domains of CHIP, the C terminus of HSP70 interacting protein. Biochemistry 45: 4749-4759.

20. Zhang M, Windheim M, Roe SM, Peggie M, Cohen P, et al. (2005) Chaperoned ubiquitylation--crystal structures of the CHIP U box E3 ubiquitin ligase and a CHIP-Ubc13-Uev1a complex. Mol Cell 20: 525-538.

21. Xu Z, Kohli E, Devlin KI, Bold M, Nix JC, et al. (2008) Interactions between the quality control ubiquitin ligase CHIP and ubiquitin conjugating enzymes. BMC Struct Biol 8: 26.

22. Vander Kooi CW, Ohi MD, Rosenberg JA, Oldham ML, Newcomer ME, et al. (2006) The Prp19 U-box crystal structure suggests a common dimeric architecture for a class of oligomeric E3 ubiquitin ligases. Biochemistry 45: 121-130.

23. Tu D, Li W, Ye Y, Brunger AT (2007) Inaugural Article: Structure and function of the yeast U-box-containing ubiquitin ligase Ufd2p. Proc Natl Acad Sci U S A 104: 15599-15606.
