# Supplementary material for: Comprehensively Surveying Structure and Function of RING Domains from Drosophila melanogaster
Source: PLoS One. 2011 Sep 2;6(9):e23863. doi: 10.1371/journal.pone.0023863 (PMC3166285; doi:10.1371/journal.pone.0023863)
Supplement: Table S8 — Summary of interolog interactions of E2s and RING-E3s from fruit fly. (PDF) [file pone.0023863.s016.pdf]

Table S8 Summary of interolog interactions of E2s and RING-E3s from fruit fly

E2s	Binding partner E3s
UBE2D1-4	CG7037 CG8293 CG9949 CG13030 CG2681 CG2617 CG9941 CG15104 CG5841 CG1134 CG8974 CG6923 CG32850 CG10277 CG2679 CG7694 CG1937 CG11982 CG13344 CG3929 CG10961 *CG14435* CG13025 CG31716 CG33144 CG34440 CG5203 CG11070
UBE2G2	CG2617 CG9941 CG15105 CG17019 CG8786 CG9941 CG8293 CG6923
UBE2I	CG15104 CG34440
UBE2L3	CG7037 CG5709 CG2218
UBE2L6	CG9949 CG13030 CG2681 CG15104 CG1134 CG13344 CG33144
UBE2N	CG8293 CG5709 CG15104 CG5841 CG11988 CG32850 CG10277 CG2679 CG3929 CG10961 *CG14435 CG15011* CG31716
UBE2W	CG2617 CG15104 CG5841 CG8293 CG1134 CG6923 CG11982 CG31716
UBE2E1	CG7037 CG8293 CG1134 CG8974 CG6923 CG32850 CG10277 CG2679 CG7694 CG11982 CG13344 CG3929 CG10961 *CG14435*
UBE2E3	CG1134 CG8974 CG6923 CG10277 CG2679 CG7694 CG11982 CG13344 CG31716
UBE2C	CG3929 CG34440
UBE2Q1	CG2617
UBE2J1	CG15104 CG8293 CG5595 CG1937
UBE2H	CG11988
UBE2R1	*CG14435*
UBE2R2	CG16982
UBE2U	CG2617 CG15105 CG5206 CG5841 CG5595 CG12218 CG4973 CG13605 CG1134 CG8974 CG32850 CG2304 CG5140 CG7694 CG1937 CG18028 CG13344 CG3929 CG17033 CG9014 CG33144 CG34440
UBE2Z	CG2304 CG2679 CG1937 CG17033 CG9014 CG33144

Notes. E2s: Class I; Class II; Class III; Class IV.

E3s: C3HC4-type; C3H2C3-type; C3HC3D-type; *C4HC3-type*; C3HGC3-type; C4C4-type; C6H3C2D-type; U-box

Ubiquitin-like E2s: (1) NEDDylation: UBE2F, UBE2M;
(2) ISGylation: UBE2L6, UBE2E1; UBE2E2, UBE2N;
(3) SUMOylation: UBE2I.

UBE2D1, UBE2D2, UBE2D3 and UBE2D4 are highly similar. Binding RING-E3s of them are usually the same.
